# Potent neutralization of SARS-CoV-2 variants by RBD nanoparticle and prefusion-stabilized spike immunogens

**DOI:** 10.1038/s41541-024-00982-1

**Published:** 2024-10-08

**Authors:** Marcos C. Miranda, Elizabeth Kepl, Mary Jane Navarro, Chengbo Chen, Max Johnson, Kaitlin R. Sprouse, Cameron Stewart, Anne Palser, Adian Valdez, Deleah Pettie, Claire Sydeman, Cassandra Ogohara, John C. Kraft, Minh Pham, Michael Murphy, Sam Wrenn, Brooke Fiala, Rashmi Ravichandran, Daniel Ellis, Lauren Carter, Davide Corti, Paul Kellam, Kelly Lee, Alexandra C. Walls, David Veesler, Neil P. King

**Affiliations:** 1 Department of Biochemistry, University of Washington, Seattle, WA USA; 2 Institute for Protein Design, University of Washington, Seattle, WA USA; 3 Department of Medicinal Chemistry, University of Washington, Seattle, WA USA; 4 Biological Physics Structure and Design Program, University of Washington, Seattle, WA USA; 5 Kymab Ltd., Babraham Research Campus, Cambridge, UK; 6 Vir Biotechnology, San Francisco, CA USA; 7 Department of Infectious Disease, Imperial College, London, UK; 8 Howard Hughes Medical Institute, University of Washington, Seattle, WA USA

**Keywords:** Protein vaccines, Vaccines

## Abstract

We previously described a two-component protein nanoparticle vaccine platform that displays 60 copies of the SARS-CoV-2 spike protein RBD (RBD-NP). The vaccine, when adjuvanted with AS03, was shown to elicit robust neutralizing antibody and CD4 T cell responses in Phase I/II clinical trials, met its primary co-endpoints in a Phase III trial, and has been licensed by multiple regulatory authorities under the brand name SKYCovione^TM^. Here we characterize the biophysical properties, stability, antigenicity, and immunogenicity of RBD-NP immunogens incorporating mutations from the B.1.351 (β) and P.1 (γ) variants of concern (VOCs) that emerged in 2020. We also show that the RBD-NP platform can be adapted to the Omicron strains BA.5 and XBB.1.5. We compare β and γ variant and E484K point mutant nanoparticle immunogens to the nanoparticle displaying the Wu-1 RBD, as well as to soluble prefusion-stabilized (HexaPro) spike trimers harboring VOC-derived mutations. We find the properties of immunogens based on different SARS-CoV-2 variants can differ substantially, which could affect the viability of variant vaccine development. Introducing stabilizing mutations in the linoleic acid binding site of the RBD-NPs resulted in increased physical stability compared to versions lacking the stabilizing mutations without deleteriously affecting immunogenicity. The RBD-NP immunogens and HexaPro trimers, as well as combinations of VOC-based immunogens, elicited comparable levels of neutralizing antibodies against distinct VOCs. Our results demonstrate that RBD-NP-based vaccines can elicit neutralizing antibody responses against SARS-CoV-2 variants and can be rapidly designed and stabilized, demonstrating the potential of two-component RBD-NPs as a platform for the development of broadly protective coronavirus vaccines.

## Introduction

SARS-CoV-2 has had a major public health impact since its emergence in late 2019^1^. The rapid development of novel vaccines against the ancestral (Wu-1) strain of SARS-CoV-2 proved an effective public health measure in preventing severe disease and death amongst the vaccinated^2,3^. However, mutations in the spike of emerging variants has dramatically reduced the serum neutralization potency of infected and immunized individuals against new variants and variants of concern (VOCs)^4,5^. For example, neutralizing antibody levels against the B.1.351 (β) variant, one of the first VOCs, dropped ~13 and ~7-9 fold in pre-infected and twice-immunized individuals respectively when compared to the ancestral strain^6^. Since then, there has only been an increase in variants originating from the B.1.1.529 lineage, with the EG.5 sublineage having 42 and 24 mutations in the full-length spike and receptor binding domain (RBD), respectively^7–9^. Although mRNA vaccines based on the full-length, membrane-anchored spike protein have proven amenable to strain updates^2,4,10,11^, there is little information on the ability of other vaccine platforms to tolerate antigen updates. This topic merits further investigation as it is relevant to updating current vaccines to address emerging variants, the suitability of various vaccine platforms for pandemic preparedness and response, and understanding the interplay between the spike mutational landscape and vaccine-elicited immune responses.

We previously developed a computationally designed receptor binding domain protein nanoparticle vaccine (RBD-NP) that elicited strong protection against the ancestral strain of SARS-CoV-2 in mice and non-human primates and met its Phase III clinical trial endpoints, leading to licensure in South Korea and the UK as well as an Emergency Use Listing from the WHO^12–15^. Although we and others have begun to explore mosaic RBD-NPs as broadly protective sarbecovirus vaccine candidates^16–18^, questions remain about how often SARS-CoV-2 vaccines should be updated to keep pace with emerging variants of concern and the amenability of various vaccine platforms to strain updates.

Here we describe variant updated RBD-NP vaccines based on the ancestral (Wu-1), B.1.351(β), and P.1(γ) lineages benchmarked against prefusion-stabilized (HexaPro) spikes^19^. We find that although the RBD-NP based upon the ancestral lineage (Wu-1-RBD-NP) was stable over 28 days, RBD-NPs displaying wild-type RBDs from these VOCs exhibited much poorer solution properties. Although introducing stabilizing mutations we previously identified in the linoleic acid binding site^20^ improved stability and immunogenicity in some cases, our results suggest that additional RBD-stabilizing mutations may be necessary to enable reliable strain updates in this vaccine platform.

## Results

**Fig. 1:**
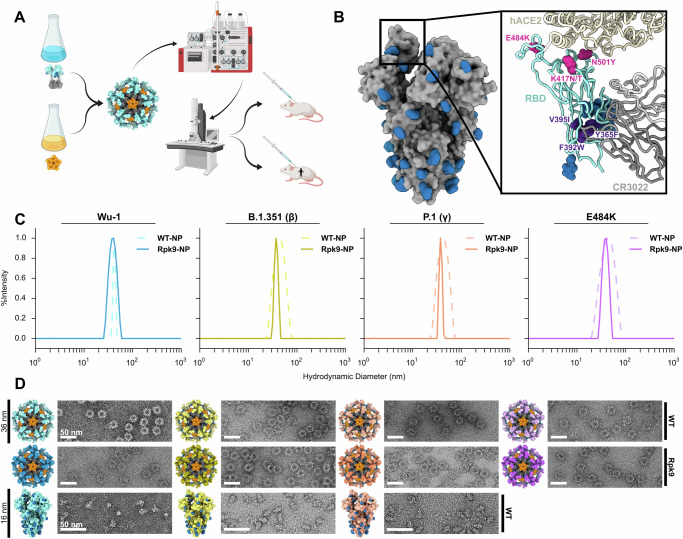
SARS-CoV-2 variant vaccine design and characterization. **A** Graphical representation of the production and characterization of SARS-CoV-2 RBD-NP vaccines, created with BioRender.com. **B** Molecular surface representation of the SARS-CoV-2 HexaPro trimer in the prefusion-stabilized conformation in gray^19^ (PDB: 6XKL). N-linked glycans in dark blue. A single RBD is boxed and expanded with bound hACE2 receptor^61^ (PDB: 6M0J) and CR3022 Fab (PDB: 6W41)^23^ shown for reference. Pink spheres indicate β and γ RBD mutations (K417N/T, E484K, and N501Y). Purple spheres indicate stabilizing Rpk9 mutations (Y365F, F392W, and V395I). **C** Representative DLS of 8 monovalent RBD-NPs with and without Rpk9 mutations. **D** Structural models of 8 monovalent RBD-NPs and 3 prefusion-stabilized HexaPro trimers alongside representative nsEM micrographs of each immunogen. All graphical representations of proteins made using ChimeraX^61^.

To gauge the manufacturability and immunogenicity of VOC vaccines based on the RBD-NP platform, we produced RBD-NPs displaying several different RBDs for immunogenicity studies (all amino acid sequences for novel proteins provided in Supplementary Table 1). All RBD-NP vaccines were based on the two-component self-assembling protein nanoparticle I53-50, which consists of 12 pentameric and 20 RBD-bearing trimeric building blocks for a total of 60 RBDs presented on each nanoparticle^12,21^. As previously described, we expressed and purified the antigen-bearing components in Expi293F cells and the I53-50B.4PT1 pentamer in *E. coli* prior to assembling the nanoparticle immunogens in vitro^16^ (Fig. 1A). We generated RBD-NPs displaying RBDs from the Wu-1, B.1.351 (β), and P.1 (γ) (K417N/T, E484K, N501Y) lineages with and without the previously reported “Rpk9” stabilizing mutations to the linoleic binding pocket (Y365F, F392W, V395I)^20^, as these were the dominant circulating strains with prominent escape mutations prior to the Omicron lineages (Fig. 1B). An RBD-NP vaccine bearing the single point mutation E484K was also created to probe the effects of this mutation on stability, antigenicity, and vaccine-elicited neutralizing antibody titers and breadth. SARS-CoV-2 strains containing this substitution escape protection by several monoclonal antibody therapies such as LY-CoV555, which resulted in discontinuation of their clinical use^22^.

Dynamic light scattering (DLS) and negative stain electron microscopy (nsEM) revealed that all eight monovalent RBD-NPs (four variants with and without Rpk9 mutations) were of the expected hydrodynamic diameter and adopted the known morphology of icosahedral I53-50-based immunogens (Fig. 1C, D). As benchmarks, we expressed prefusion-stabilized (HexaPro) spike proteins bearing the same VOC mutations (except for the E484K point mutant) in Expi293F cells, purified them by immobilized metal-ion affinity chromatography (IMAC), and structurally validated them by nsEM (Fig. 1D). These data suggested that the RBD-I53-50-NP and HexaPro immunogen formats are robust to variations in RBD or spike protein sequences.

**Fig. 2:**
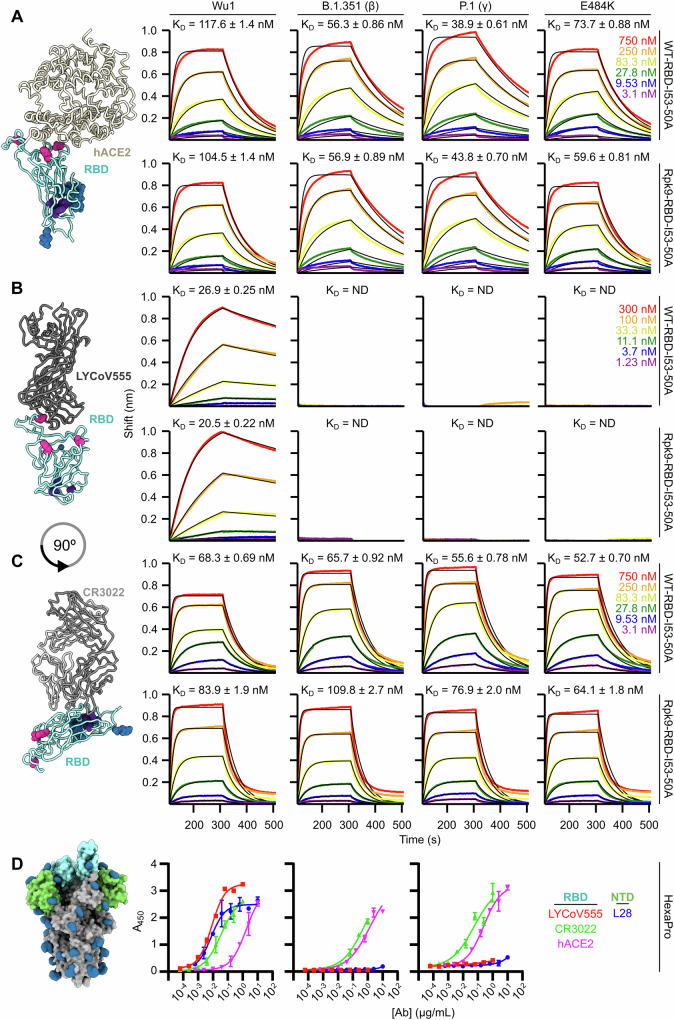
Antigenic characterization of variant antigens. *Left*, Models of RBD with bound (**A**) hACE2 receptor, (**B**) LY-CoV555^24^ (PDB: 7KMG), and **C)** CR3022 Fab, depicted as in Fig. 1. *Right*, Binding of (**A**) monomeric hACE2, (**B**) LY-CoV555, and (**C**) CR3022 Fab to immobilized VOC-RBD-I53-50A trimers. Data are shown in colors and global fits as black lines, with the *K*_D_ of each interaction indicated. **D** *Left*, Molecular surface representation of SARS-CoV-2 HexaPro prefusion-stabilized trimer^19^ (PDB: 6XKL) with RBDs and NTDs highlighted in light blue and green, respectively. *Right*, ELISA binding titers to hACE2 receptor, RBD-specific IgG (LY-CoV555 and CR3022), and NTD-specific IgG (S2L28). All graphical representations of proteins made using ChimeraX^61^. Error bars represent standard error of mean from three replicates.

To understand the effects of VOC and Rpk9 mutations on antigenicity, we used biolayer interferometry (BLI) to measure the binding of the trimeric antigen-bearing components to monomeric hACE2 receptor and two Fabs from antibodies that bind the ancestral Wu-1 RBD, LY-CoV555 and CR3022^23–25^. Since some VOC mutations are in the receptor binding motif, we determined the affinity of each component for hACE2, finding that there were minimal differences between variant-matched WT and Rpk9 antigen-bearing components (Fig. 2A). We then compared binding to LY-CoV555, expecting that the E484K substitution would abrogate binding as previously reported^22^. As expected, all antigen-bearing components with the E484K substitution failed to bind LY-CoV555 Fab at any concentration tested (1.23–300 nM) (Fig. 2B). Finally, we determined the affinity of each component to CR3022 Fab to understand the potential impact of the nearby Rpk9 mutations on this conserved cryptic epitope^23^. Our findings indicate that Rpk9 mutations result in a minimal but consistent reduction in CR3022 binding, with no pair of designs showing even a two-fold difference in *K*_D_ (Fig. 2C).

Antigenic characterization of Wu-1 and VOC HexaPros by ELISA recapitulated the BLI obtained with trimeric RBD-I53-50A components. Both VOC HexaPros lost all binding to LY-CoV555 while maintaining similar antigenic characteristics at the hACE2 and CR3022 binding sites (Fig. 2D). We also used the NTD-directed S2L28 monoclonal antibody^26^ to evaluate potential differences in antigenicity among the three HexaPro NTDs. Both VOC HexaPros failed to bind S2L28 mAb, indicating antigenic remodeling of this highly variable domain. In summary, all HexaPros and antigen-bearing components, with or without the Rpk9 mutations, were antigenically intact, exhibiting the expected binding profiles.

**Fig. 3:**
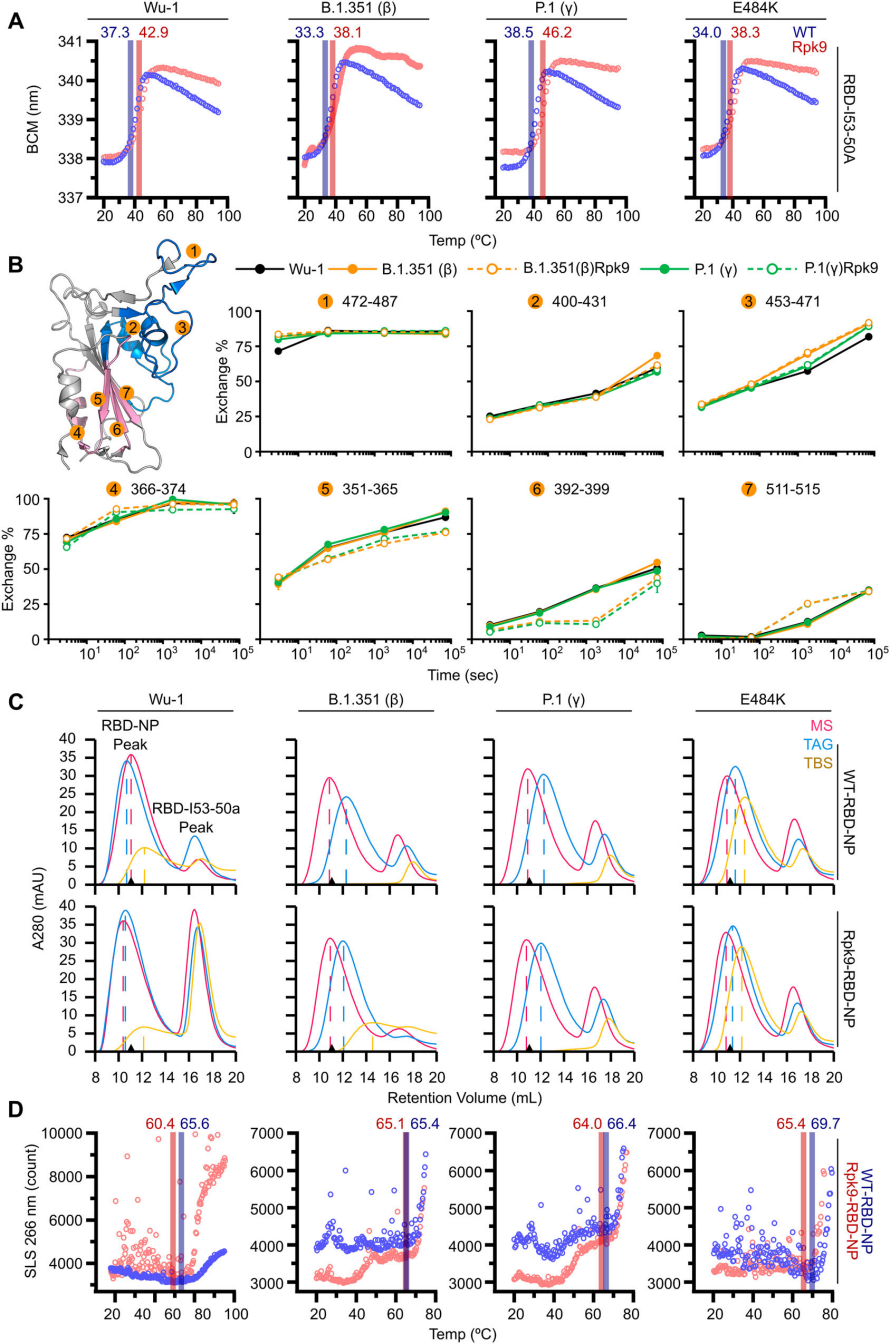
Stabilizing effects of Rpk9 mutations in variant RBD components and NPs. **A** Representative melting curves of RBD-I53-50A trimers with and without Rpk9 mutations. Melting temperatures (T_m_) are indicated. **B** Hydrogen/deuterium exchange mass spectrometry of selected RBD peptides. *Left*, RBD structure with observed peptides numbered and regions where the VOC-related and Rpk9 mutations reside represented in blue and pink, respectively. **C** SEC chromatograms of RBD-NPs in three different buffers. MS: 50 mM Tris pH 7.4, 185 mM NaCl, 100 mM arginine-HCl, 4.5% v/v glycerol, 0.75% w/v CHAPS; TAG: 50 mM Tris pH 8, 150 mM NaCl, 100 mM arginine-HCl, 5% v/v glycerol; TBS: 50 mM Tris pH 8, 150 mM NaCl. Major peak at ~10.5 mL represents assembled NP and minor peak ~17 mL represents excess VOC-RBD-I53-50A. Black triangle on the x axis represents the Wu-1-RBD-NP peak in MS. **D** Representative aggregation profiles of purified RBD-NPs, with temperature (T_agg_) indicated.

Next, to determine the impact of the Rpk9 mutations on VOC antigen stability, thermal melts and hydrogen/deuterium exchange mass spectrometry (HDX-MS) were performed on the trimeric I53-50A components. Melting temperatures for each VOC antigen-bearing component were obtained by nano differential scanning fluorimetry (nanoDSF), monitoring intrinsic tryptophan fluorescence from 20 °C to 95 °C. The melting temperature (T_m_) of each Rpk9-containing component was about 5 °C higher than its strain-matched WT construct, revealing that the Rpk9 mutations increase the thermal stability of VOC RBDs as they did for the ancestral Wu-1 RBD (Fig. 3A). Previously, the Rpk9 mutations were reported to increase the T_m_ of Wu-1-RBD-I53-50A from ~49 °C to ~54 °C (ref. ^20^). Here, we observed an increase from ~37 °C to ~43 °C. We attribute the difference to the inclusion of CHAPS, a zwitterionic detergent, in the buffers used in the present study. Nevertheless, the Rpk9 mutations consistently increase T_m_ by ~5–6 °C in the Wu-1-RBD-I53-50A regardless of the differences in buffer. The Rpk9 mutations also reduced hydrogen/deuterium exchange in the region of the linoleic acid binding site, specifically in peptides spanning residues 351-365 and 392-399 (Fig. 3B). However, there was no difference in exchange between variant-matched RBDs in peptides comprising residues 400-431, 453-471, and 472-487, indicating that—in accordance with our antigenic characterization—the Rpk9 mutations have minimal effect on the local stability of surface neutralizing epitopes.

We previously showed that the Rpk9 mutations improved the solution properties of the Wu-1-RBD-NP, particularly in excipient-free buffers lacking detergent^20^. To determine the effects of the Rpk9 mutations in VOC-RBD-NPs, we utilized SEC in decreasingly excipient-rich buffers. We found that Rpk9-RBD-NPs did not show major differences in solution properties compared to the WT-RBD-NPs (Fig. 3C). Unexpectedly, the temperatures at which the Rpk9-RBD-NPs began to aggregate were slightly lower than WT-RBD-NPs (Fig. 3D). This differs from previously published data on Rpk9-Wu-1-RBD-NP^20^, which is likely explained by the use of different buffers (previously 50 mM Tris pH 8, 150 mM NaCl, 100 mM arginine-HCl, 5% v/v glycerol versus currently 50 mM Tris pH 7.4, 150 mM NaCl, 100 mM arginine-HCl, 5% v/v glycerol, 0.75% w/v CHAPS). Overall, although the Rpk9 mutations led to increases in the stability of components bearing the β and γ RBDs, nanoparticles displaying these RBDs had relatively poor solution properties compared to the ancestral Wu-1 immunogen, even in excipient-rich buffers. Interestingly, NPs displaying the RBD of the more recent OBA.4/5 variants required the Rpk9 mutation for expression and assembly: we were unable to detect expression of the wild-type OBA.4/5 RBD fused to I53-50A. However, RBD-NPs containing the OXBB.1.5 variant RBD could be readily produced in high yield and were well-behaved in solution (Supplementary Fig. 1).

**Fig. 4:**
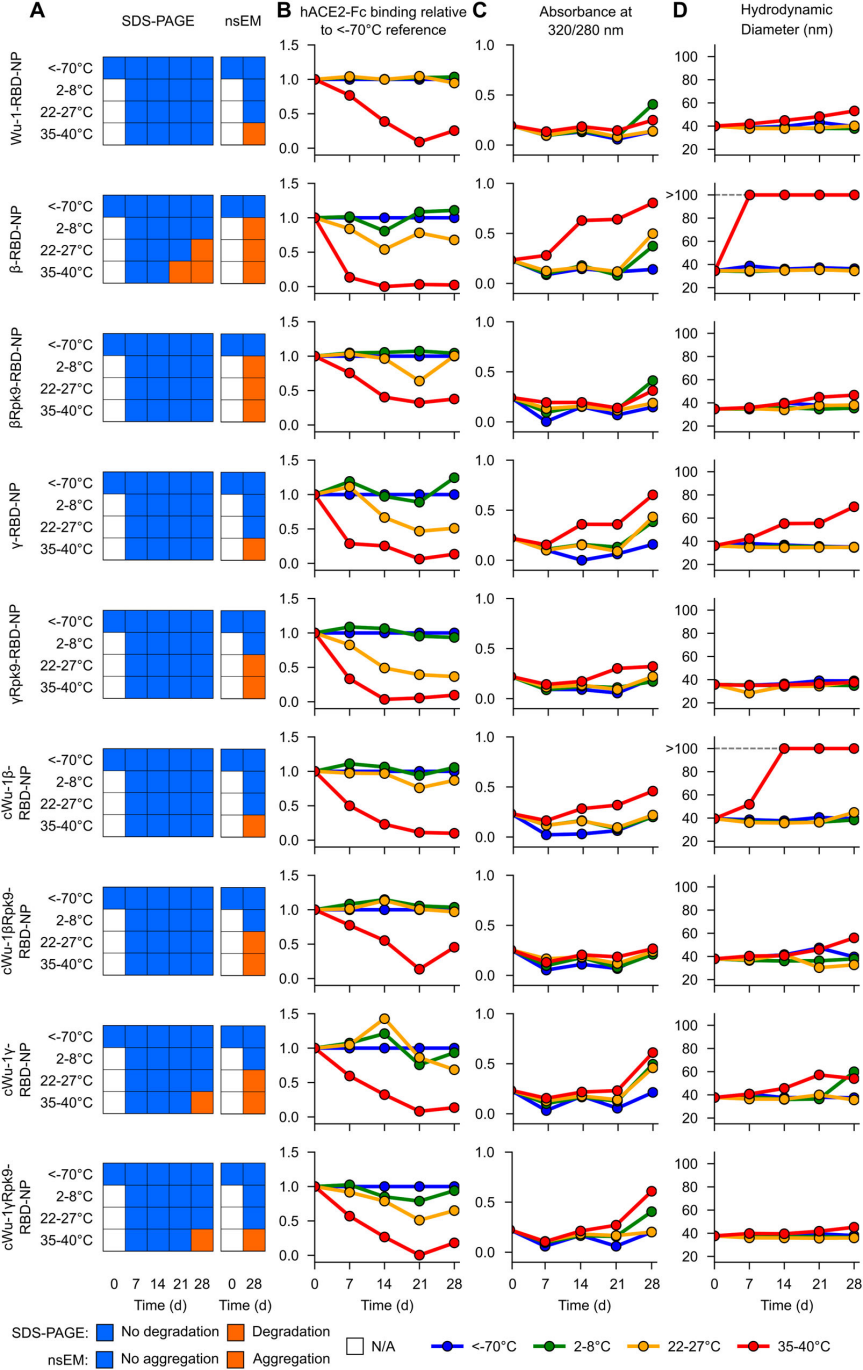
Shelf-life stability of SARS-CoV-2 variant RBD-NPs. **A** Summary of SDS-PAGE and nsEM stability data over 4 weeks. N/A, not assessed. Cocktail formulations include Wu-1-RBD-NP formulated with β-RBD-NP, βRpk9-RBD-NP, γ-RBD-NP, or γRpk9-RBD-NP. Detailed RBD-NP construct information can be found in Supplementary Table 2. **B** Binding to hACE2-Fc over 4 weeks. Immunogens were analyzed for hACE2-Fc binding by BLI after storage at the various temperatures, normalizing against a <-70°C reference sample at each time point. **C** Summary of UV/vis stability data over 4 weeks, using 320/280 nm absorbance ratio as a measure of particulate scattering. **D** Hydrodynamic diameter and polydispersity measured by DLS over 4 weeks. Raw data provided in Supplementary File 1.

To define the shelf-life stability of the Rpk9-bearing VOC vaccines, we conducted a 28-day stability assessment. A suite of biophysical characterization assays was used to analyze the physical and antigenic integrity of RBD nanoparticle immunogens at four temperatures: <-70, 2–8, 22–27, and 35–40 °C. As previously described, Wu-1-RBD-NP was stable over the 4-week study^12^, showing changes only at 35–40 °C, where some aggregation was observed at day 28 by nsEM and a slight increase in hydrodynamic diameter by DLS (Fig. 4 and Supplementary File 1). Antigenicity, as noted by hACE2-Fc binding relative to reference samples stored at −70 °C, decreased starting at day 7 only when the sample was incubated at 35–40 °C. At all other temperatures, there were no major differences over the 28-day study in appearance by SDS-PAGE or nsEM, antigenicity, UV/vis absorbance, or hydrodynamic diameter.

Consistent with previous reports^6^, the β-RBD-NP proved the least stable throughout the study, showing degradation at 22–27 and 35–40 °C by SDS-PAGE; aggregation from 2–8 °C up to 35–40 °C by nsEM, UV/vis, and DLS; and a progressive loss of hACE2 binding with increasing temperature. The γ-RBD-NP showed similar trends at mid- to high-temperature, although they were less pronounced. Introduction of the Rpk9 mutations dramatically increased the stability of these immunogens by maintaining the expected hydrodynamic diameters throughout the study duration, although in each case there was still a decrease in antigenicity over time, especially at 35–40 °C.

We also compared the shelf-life stabilities of nanoparticle cocktails in which the Wu-1-RBD-NP was mixed with variant RBD-NPs. When combined with the β-RBD-NP (cWu-1β-RBD-NP), Wu-1-RBD-NP seemed to improve temperature-induced aggregation and loss of antigenicity compared to β-RBD-NP alone. The differences were not as dramatic in the cWu-1γ-RBD-NP compared to monovalent γ-RBD-NP, but generally followed the same pattern. Again, introducing the Rpk9 mutations into the variant RBD-NPs improved immunogen stability in cWu-1-βRpk9-RBD-NP (Wu-1-RBD-NP + βRpk9-RBD-NP) and cWu-1-γRpk9-RBD-NP (Wu-1-RBD-NP + γRpk9-RBD-NP) compared to cWu-1β-RBD-NP and cWu-1γ-RBD-NP, respectively, but did not completely prevent loss of antigenicity at 35–40 °C.

Collectively, these data show that the Rpk9 mutations increased the stability and solution properties of variant RBD nanoparticles. Furthermore, they establish that the Wu-1-RBD-NP has higher physical and antigenic stability than nanoparticles displaying the β and γ variant RBDs, a finding with implications for vaccine updates.

**Fig. 5:**
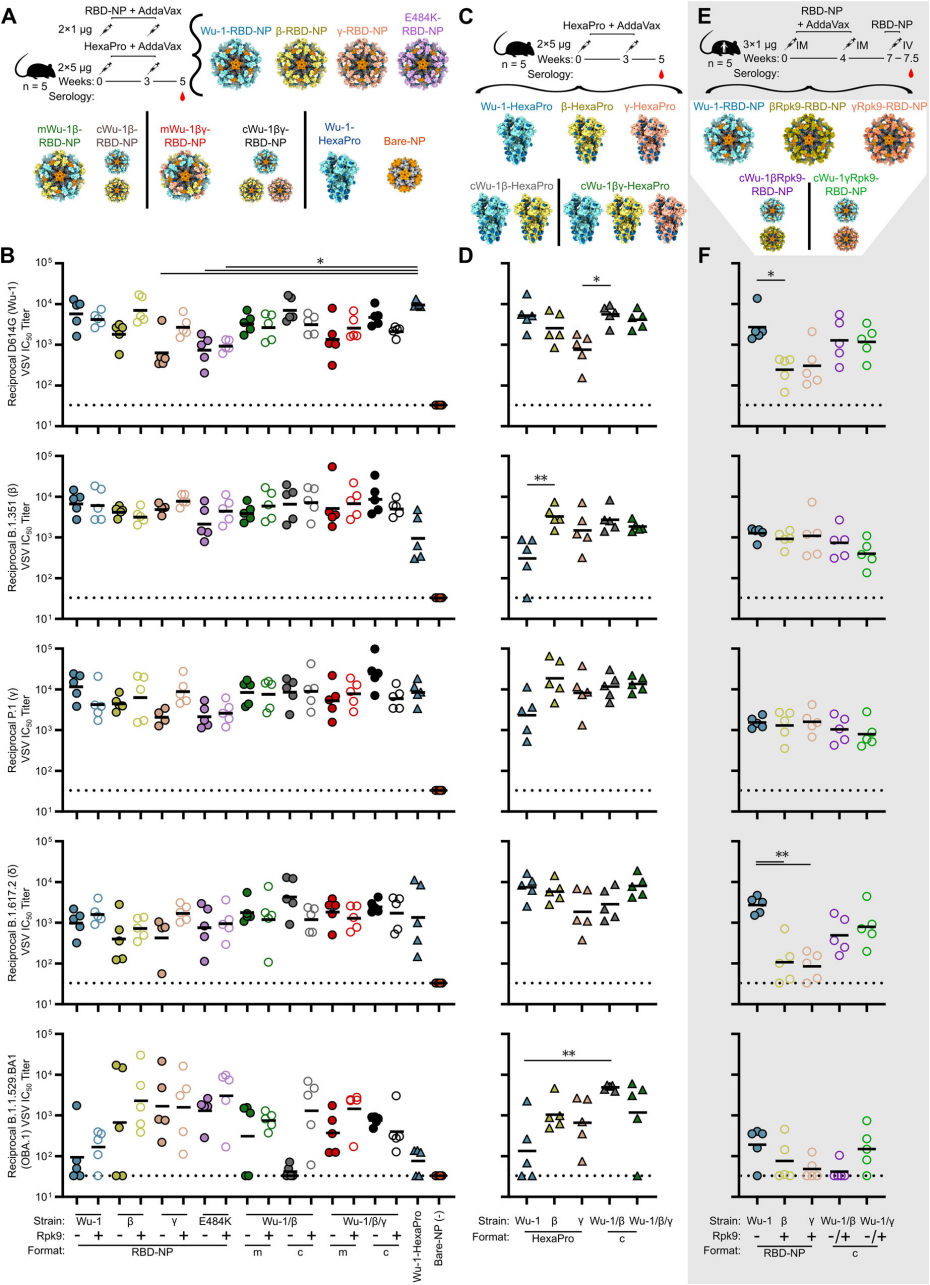
Variant RBD-NP vaccines elicit high neutralizing Ab titers to SARS-CoV-2 variants in mice. **A**, **C** Immunization scheme of VOC-RBD-NP, VOC-HexaPro, and the NP scaffold (Bare-NP) vaccines in Balb/c mice. Structural models of VOC-RBD-NPs, VOC-HexaPro, and Bare-NP vaccines. **B**, **D** Two weeks post boost neutralizing Ab titers for monovalent (RBD-NP), mosaics (m), and cocktails (c) of VOC with and without Rpk9 (+/−) mutations, Bare-NP, WT-VOC-HexaPro, and cocktails (c) of WT-VOC-HexaPro against various VOC pseudoviruses. Background neutralization is denoted by a dotted line. **E** Immunization scheme of VOC-RBD-NP vaccines in Darwin mice. Structural models of VOC-RBD-NP vaccines. **F** 0.5 week post IV boost neutralizing Ab titers for VOC-RBD-NP vaccines against various VOC pseudoviruses. Cocktail formulations including Wu-1-RBD-NP were formulated with βRpk9-RBD-NP or γRpk9-RBD-NP. All graphical representations of proteins made using ChimeraX^61^. Detailed RBD-NP immunogen information can be found in Supplementary Table 2. Kruskal–Wallis tests were performed to compare two groups to determine whether they were statistically different. Significance is indicated with stars: ^∗^*p* < 0.05; ^∗∗^*p* < 0.01; and non-significant differences are not shown.

Finally, we immunized mice with 17 different vaccine candidates including monovalent Wu-1, β, γ, and E484K RBD-NPs with and without the Rpk9 mutations; mosaic NPs (mRBD-NPs) co-displaying Wu-1/β or Wu-1/β/γ RBDs on the same nanoparticle; cocktails of NPs (cRBD-NPs) consisting of mixtures of monovalent NPs; and individual or cocktails of prefusion-stabilized (HexaPro) Wu-1, β, and γ spike trimers (Fig. 5A, C and Supplementary File 1). Groups of five BALB/c mice were first immunized at weeks 0 and 3 with 1 μg of RBD-NPs or 5 μg of Wu-1 HexaPro, and serum neutralizing antibody titers were tested against pseudotyped VSV bearing G614, β, γ, B.1.617.2 (δ), or B.1.1.529 (Omicron BA.1) S. All immunogens elicited neutralizing antibodies 2 weeks post-boost against all viruses tested, although there were subsets of mice in several groups that did not yield detectable neutralizing activity against BA.1 (Fig. 5B). A negative control immunogen consisting of bare I53-50 lacking any displayed antigen did not induce any neutralizing activity, as expected. The Rpk9-RBD-NPs tended to elicit comparable or higher neutralizing antibody (nAb) titers than their WT counterparts, although none of the differences were statistically significant. The Wu-1-RBD-NP elicited high nAb titers against all VOC pseudoviruses tested, with geometric mean titers (GMTs) ranging from 1 × 10^3^ to 1.2 × 10^4^, except B.1.1.529 (BA.1; GMT 94.5). Introduction of the stabilizing Rpk9 mutations to the β-RBD-NP improved neutralization of all vaccine-mismatched pseudoviruses tested (Wu-1, γ, δ, and BA.1) by 3.9×, 1.4×, 1.8×, and 3.4× respectively, while maintaining similar nAb titers to the vaccine-matched pseudovirus. The Rpk9 mutations also improved nAb titers elicited by the γ-RBD-NP against Wu-1, β, γ, and δ S by 4.3×, 1.6×, 4.3×, and 4.1×, while GMT nAb titers against BA.1 were relatively unchanged. Interestingly, although the Rpk9 mutations did not consistently improve nAb titers in mice immunized with E484K-RBD-NP, these nanoparticles induced nAb titers against BA.1 14× and 18× higher than the corresponding ancestral (Wu-1) RBD-NPs. The Rpk9 mutations generally did not seem to improve nAb titers in mosaic and cocktail formulations, except against BA.1, where they increased nAb titers by 2.4×, 31×, and 3.9× in the mosaic bivalent, cocktail bivalent, and mosaic trivalent RBD-NP groups, respectively. Regardless of the Rpk9 mutations, NP vaccines comprising multiple VOC RBDs elicited comparable titers to all VOCs, both vaccine-matched (Wu-1, β, or γ) or -mismatched (δ or OBA.1), compared to their monovalent counterparts.

To understand if the inclusion of the N-terminal domain (NTD) and S2 subunit in immunogens improves elicitation of vaccine-mismatched nAbs, we immunized BALB/c mice with 5 μg of VOC-HexaPro trimers and cocktails thereof (Fig. 5C). Although there is some evidence suggesting that incorporation of the NTD can elicit cross-reactive Abs, a minority of nAbs are NTD-directed^27–30^. Mice vaccinated with Wu-1-HexaPro, β-HexaPro, and γ-HexaPro elicited high levels of neutralizing antibodies to their vaccine-matched pseudoviruses, however those immunized with Wu-1-HexaPro experienced substantial drops in vaccine-mismatched nAb titers compared to the other HexaPro immunogens, except for δ neutralization (Fig. 5D). Both cocktail formulations of VOC-HexaPros elicited high levels of nAbs across all VOC tested, including the highly divergent δ and BA.1 strains. We conclude that HexaPro immunogen formulations including the β and γ lineages provide increased levels of neutralizing antibodies compared to those containing solely Wu-1-HexaPro in heterologous pseudovirus neutralization.

Based on the stability data and immunogenicity data from the two previous studies, we selected five RBD-NP vaccine candidates for evaluation in transgenic human Ig locus mice (Darwin mice) (Fig. 5E). Wu-1-RBD-NP elicited significantly higher and comparable G614 neutralization compared to VOC-Rpk9-RBD-NPs and cocktails of Wu-1- and VOC-Rpk9-RBD-NPs, respectively (Fig. 5F). Against both β and γ pseudoviruses, the Wu-1-RBD-NP, VOC-Rpk9-RBD-NPs, and cocktail immunogens generated equivalent nAb titers. Surprisingly, in Darwin mice immunization with Wu-1-RBD-NP yielded the highest nAb titers against δ and BA.1. Darwin mice have a human Ig repertoire^31^ and therefore may generate human-like polyclonal responses against the RBD. Both human and primate polyclonal responses have previously been reported to hyperfocus on position 484 in the RBD, which may explain some of the distinct sensitivities of neutralization breadth in BALB/c mice compared to Darwin mice^16,32,33^. Additionally, Darwin mice were bred from C57BL/6 and 129S7 mice, and the former have previously been shown to generate lower binding and neutralizing antibody titers than BALB/c mice in response to immunization^2^. In summary, we conclude that Wu-1-RBD-NP elicits the most consistent levels of vaccine-matched and -mismatched neutralizing antibodies in humanized mice, while c/mRBD-NP and HexaPro formulations containing antigens from multiple VOCs maintain the most consistent neutralizing antibody levels in BALB/c mice.

## Discussion

RBD-I53-50 nanoparticles have proven to be robust and versatile immunogens for coronavirus vaccines. The Wu-1-RBD-NP originally described in 2020 was shown to elicit high levels of neutralizing antibodies in Phase III clinical trials^14^ and has been licensed for use in multiple countries, establishing computationally designed protein nanoparticle vaccines as a commercial vaccine platform. Despite the inclusion of only the RBD, the performance of RBD-NP vaccines has proven similar to vaccines that also include the NTD and the conserved S2 fusion machinery, including against vaccine-mismatched variants. For example, non-human primates boosted with the βRpk9-RBD-NP described here 6 months after a primary immunization series of either the Wu-1-RBD-NP or Wu-1 HexaPro were similarly protected against SARS-CoV-2 Omicron BA.1 challenge 6 months later^34^. Furthermore, monoclonal antibodies isolated from both sets of animals showed similar potency and breadth against a diverse panel of sarbecoviruses^35^. Additionally, administration of a third dose of the licensed RBD-NP vaccine SKYCovione™ in humans 7 months after the primary series increased neutralizing antibody titers against Omicron BA.1 75-fold, reaching levels 25-fold higher than peak titers after the primary series^14^. Similar results against Omicron BA.1 were observed after a third dose of mRNA-1273^36^. These data, together with the production of RBD-NPs displaying various VOC (this work) and sarbecovirus^16^ RBDs, highlight RBD-I53-50 nanoparticles as a platform for coronavirus vaccine development.

However, our data reinforce that the properties of immunogens based on different SARS-CoV-2 variants can differ substantially and could affect the viability of variant vaccine development. For example, the poor stability and solution properties of the recombinant β spike protein were noted soon after the emergence of B.1.351^37^, and we observed similar effects in the β- and γ-RBD-NPs. Both of these RBD-NPs exhibited poor expression yields and a marked tendency to aggregate compared to the Wu-1-RBD-NP. Introducing the stabilizing Rpk9 mutations improved expression and the solution properties of these RBD-NPs somewhat, but did not fully rescue them. The OBA.4/5-RBD-NP could only be produced when the Rpk9 mutations were added, as expression was undetectable without them. By contrast, the OXBB.1.5-RBD-NP could be readily produced in high yield and was well-behaved in solution. These differences between individual variants and the inability of the Rpk9 mutations to ubiquitously improve VOC-RBD stability is a limitation of the current work. Identifying additional stabilizing mutations that are portable across sarbecovirus RBDs would help ensure that effective vaccines could be rapidly produced for any new variant^38,39^. Understanding how mutations that arise in new variants affect pre-existing immunity has also proven helpful for informing when vaccine strain updates are needed^40–43^.

Since the emergence of the virus, SARS-CoV-2 vaccine development efforts worldwide have shown that the time required to manufacture protein-based vaccines presents a challenge for updating vaccines in response to ongoing antigenic evolution. By contrast, the significantly shorter timelines for mRNA vaccine manufacture have facilitated the development of updated vaccines^44–46^. Approaches that combine the potency of protein nanoparticle vaccines with the manufacturing speed of mRNA, such as mRNA vaccines that encode secreted protein nanoparticle immunogens^47^, could become a powerful platform for vaccine development against emerging and evolving infectious diseases. Alternatively, broadly protective protein nanoparticle vaccines that induce immunity against multiple related viruses may preclude the need to frequently update vaccines. Several multivalent RBD nanoparticle vaccines have yielded promising preclinical data and are advancing toward clinical trials^16–18^. These vaccines utilize the mosaic nanoparticle display technology that was originally developed for influenza vaccines^48–50^ and is currently being evaluated clinically (NCT04896086 and NCT05968989). We note that the multivalent RBD-I53-50 nanoparticle vaccine currently in preclinical development extends the work presented here by displaying stabilized RBD antigens from several different sarbecoviruses^51^. The variable behavior we observed in the present study from protein-based vaccines derived from different SARS-CoV-2 isolates highlights the need for technologies such as mosaic nanoparticle display and mRNA-launched nanoparticles that may facilitate the rapid development of protective vaccines for antigenically variable families of viruses.

## Methods

### Cell lines

Expi293F cells are derived from the HEK293F cell line (Life Technologies). Expi293F cells were grown in Expi293 Expression Medium (Life Technologies), cultured at 36.5 °C with 8% CO_2_ and shaking at 150 rpm. HEK293T/17 is a female human embryonic kidney cell line (ATCC). The HEK-ACE2 adherent cell line was obtained through BEI Resources, NIAID, NIH: Human Embryonic Kidney Cells (HEK293T) Expressing Human Angiotensin-Converting Enzyme 2, HEK293T-hACE2 Cell Line, NR-52511. The VeroE6-TMPRSS2 cell line is an African Green monkey kidney cell line expressing TMPRSS2 (ref. ^52^). All adherent cells were cultured at 37 °C with 5% CO_2_ in flasks with DMEM + 10% FBS (Hyclone) + 1% penicillin-streptomycin. Cell lines other than Expi293F were not tested for mycoplasma contamination nor authenticated.

### Mice

Four week-old female BALB/c mice (order code 047, BALB/cAnNHsd strain) were obtained from Envigo, and maintained in a specific pathogen-free facility at the University of Washington, Seattle, WA, accredited by the American Association for the Accreditation of Laboratory Animal Care International (AAALAC). Animal procedures were performed under the approvals of the Institutional Animal Care and Use Committee (IACUC) of University of Washington, Seattle, WA. Kymab, a Sanofi Company’s proprietary IntelliSelect™ transgenic mouse platform, known as Darwin, has complete human antibody loci with a non-rearranged human antibody variable and constant germline repertoire. Consequently, the antibodies produced by these mice are fully human.

### Plasmid construction

The Wu-1-SARS-CoV-2, B.1.351(β)-SARS-CoV-2, P.1(γ)-SARS-CoV-2, and E484K RBDs (N-RFPN…KKST-C) were genetically fused to the N terminus of the trimeric I53-50A nanoparticle component using a 16-residue glycine and serine linker. The RBD-16GS-I53-50A fusions were cloned into pCMV/R using the Xba1 and AvrII restriction sites using Gibson assembly or by GenScript. All RBD-bearing components contained an N-terminal mu-phosphatase signal peptide and a C-terminal octa-histidine tag. Rpk9 mutants were designed the same way except with mutations at Y365F, F392W, V395I. The Wu-1, B.1.351(β), and P.1(γ)-SARS-CoV-2 S-6P ectodomain trimers were synthesized by GenScript into pCMV/R with an N-terminal BM40 signal peptide and a C-terminal T4 fibritin foldon trimerization domain and octa-histidine tag. The constructs contained the HexaPro mutations (proline substitutions at residues F817P, A892P, A899P, A942P, K986P, and V987P) and _682_SGAG_685_ substitution at the furin cleavage site.

### Transient transfection

Proteins were produced using endotoxin-free DNA in Expi293F cells grown in suspension using Expi293F expression medium (Life Technologies) at 33 °C, 70% humidity, 8% CO_2_ rotating at 150 rpm. The cultures were transfected using PEI-MAX (Polyscience) with cells grown to a density of 3.0 million cells per mL and cultivated for 3 days. Supernatants were clarified by centrifugation (5 min at 4000 rcf), addition of PDADMAC solution to a final concentration of 0.0375% (Sigma Aldrich, #409014), and a second centrifugation (5 min at 4000 rcf).

### Microbial plasmid construction, protein expression and purification of I53-50B.4PT1

I53-50B.4PT1 plasmid was synthesized by GenScript in pET29b between the NdeI and XhoI restriction sites with a double-stop codon just before the C-terminal polyhistidine tag. Protein was expressed in Lemo21(DE3) cells (NEB) in LB (10 g Tryptone, 5 g Yeast Extract, 10 g NaCl) grown in a 10 L BioFlo 320 Fermenter (Eppendorf). At inoculation, impeller speed was set to 225 rpm, SPLM set to 5 with O_2_ supplementation as part of the dissolved-oxygen aeration cascade, and the temperature set to 37 °C. At the onset of a DO spike (OD ~ 12), the culture was fed with a bolus addition of 100 mL of 100% glycerol and induced with 1 mM IPTG. During this time, the culture temperature was reduced to 18 °C, and O_2_ supplementation was ceased, with expression continuing until an OD ~ 20. The culture was harvested by centrifugation and the pellets were resuspended in PBS, homogenized, and then lysed by microfluidization using a Microfluidics M110P at 18,000 psi using 3 discrete passes. Following sample clarification by centrifugation (24,000 g for 30 min), the supernatant was discarded, and protein was extracted from the inclusion bodies. First, the pellet was washed with PBS supplemented with 0.1% Triton X-100, pH 8.0. After this initial wash and sample clarification by centrifugation, the pellet was washed with PBS supplemented with 1 M NaCl, pH 8.0. Following the second wash, the protein was extracted from the pellet into PBS supplemented with 2 M urea and 0.75% CHAPS (3-[(3-Cholamidopropyl)dimethylammonio]-1-propanesulfonate), pH 8.0. Following extraction, the sample was applied to a DEAE Sepharose FF column (Cytiva) on an ӒKTA Avant150 FPLC system (Cytiva). After sample binding, the column was washed with 5 column volumes (CV) of PBS supplemented with 0.1% Triton X-100, pH 8.0, followed by a 5 CV wash with PBS supplemented with 0.75% CHAPs, pH 8.0 in series. The protein was eluted with 3 CV of PBS supplemented with 500 mM NaCl, pH 8.0. After purification, fractions were pooled and concentrated in 10 K molecular weight cutoff (MWCO) centrifugal filters (Millipore), sterile-filtered (0.22 μm), and tested to confirm low endotoxin levels before use in nanoparticle assembly.

### Protein purification

Proteins containing His tags were purified from clarified supernatants via a batch bind method where each clarified supernatant was supplemented with 1 M Tris-HCl pH 8.0 to a final concentration of 45 mM and 5 M NaCl to a final concentration of ∼310 mM. Talon cobalt affinity resin (Takara) (for S-6P) or Nickel Sepharose Excel resin (Cytiva) (for VOC-RBD-I53-50A) were added to the treated supernatants and allowed to incubate for 15 min with gentle shaking. Resin was collected using vacuum filtration with a 0.2 μm filter and transferred to a gravity column. The resin was washed with 20 mM Tris pH 8.0, 300 mM NaCl, and the protein was eluted with 3 CV of 20 mM Tris pH 8.0, 300 mM NaCl, 300 mM imidazole. The batch bind process was then repeated and the first and second elutions combined. SDS-PAGE was used to assess purity. VOC-RBD-I53-50A fusion protein IMAC elutions were concentrated to >1 mg/mL and subjected to three rounds of dialysis into 50 mM Tris pH 7.4, 185 mM NaCl, 100 mM L-arginine, 4.5% glycerol, and 0.75% w/v CHAPS (MAGiC Sauce, MS or Mixture of Arginine, Glycerol, in CHAPS) in a hydrated 10 K MWCO dialysis cassette (Thermo Scientific). VOC-HexaPro elutions were concentrated to >0.5 mg/mL and subjected to three rounds of dialysis into 50 mM Tris pH 8, 185 mM NaCl, 0.25% w/v L-histidine, 5% v/v glycerol in a hydrated 10 K MWCO dialysis cassette (Thermo Scientific). Clarified supernatants of cells expressing monoclonal antibodies and human ACE2-Fc were purified using a MabSelect PrismA 2.6 × 5 cm column (Cytiva) on an ӒKTA Avant150 FPLC (Cytiva). Bound antibodies were washed with five CV of 20 mM NaPO_4_, 150 mM NaCl pH 7.2, then five CV of 20 mM NaPO_4_, 1 M NaCl, pH 7.4 and eluted with three CV of 100 mM glycine at pH 3.0. The eluate was neutralized with 2 M Trizma base to 50 mM final concentration. SDS-PAGE was used to assess purity.

### In vitro nanoparticle assembly and purification

Total protein concentration of purified individual nanoparticle components was determined by measuring absorbance at 280 nm using a UV/vis spectrophotometer (Agilent Cary 8454) and calculated extinction coefficients. The assembly steps were performed at room temperature with addition in the following order: VOC-RBD-I53-50A trimeric fusion protein, followed by additional buffer (50 mM Tris pH 7.4, 185 mM NaCl, 100 mM L-arginine, 4.5% glycerol, and 0.75% w/v CHAPS) as needed to achieve desired final concentration, and finally I53-50B.4PT1 pentameric component (in 50 mM Tris pH 8, 500 mM NaCl, 0.75% w/v CHAPS), with a molar ratio of VOC-RBD-I53-50A:I53-50B.4PT1 of 1.1:1. All VOC-RBD-NP in vitro assemblies were incubated briefly at room temperature before subsequent purification by SEC in order to remove residual unassembled VOC-RBD-I53-50A component. A Superose 6 Increase 10/300 GL column was used for nanoparticle purification. Assembled nanoparticles were purified in 50 mM Tris pH 7.4, 185 mM NaCl, 100 mM Arginine, 4.5% v/v glycerol, and 0.75% w/v CHAPS, and elute at ∼11 mL on the Superose 6 column. Assembled nanoparticles were sterile-filtered (0.22 μm) immediately prior to column application and following pooling of fractions.

### UV/vis

UV/vis absorbance was measured using an Agilent Technologies Cary 8454. Samples were applied to a 10 mm, 50 μL quartz cell (Starna Cells, Inc.) and absorbance was measured from 180 to 1000 nm. Net absorbance at 280 nm, obtained from measurement and single reference wavelength baseline subtraction, was used with calculated extinction coefficients and molecular weights to obtain protein concentration. The ratio of absorbance at 320/280 nm was used to determine relative aggregation levels in real-time stability study samples. Samples were diluted with respective purification/instrument blanking buffers to obtain an absorbance between 0.1 and 1.0. All data produced from the UV/vis instrument was processed in the 845x UV/visible System software.

### Endotoxin measurements

Endotoxin levels in protein samples were measured using the EndoSafe Nexgen-MCS System (Charles River). Samples were diluted 1:100 in Endotoxin-free LAL reagent water, and applied into wells of an EndoSafe LAL reagent cartridge. Charles River EndoScan-V software was used to analyze endotoxin content, automatically back-calculating for the dilution factor. Endotoxin values were reported as EU/mL which were then converted to EU/mg based on UV/vis measurements. Our threshold for samples suitable for immunization was <50 EU/mg.

### Dynamic light scattering

Dynamic light scattering (DLS) was used to measure hydrodynamic diameter (Dh) and % Polydispersity (%Pd) of RBD-NPs, mRBD-NPs, and cRBD-NPs on an UNcle Nano-DSF (UNchained Laboratories). Sample was applied to a 8.8 µL quartz capillary cassette (UNi, UNchained Laboratories) and measured with 10 acquisitions of 5 s each, using auto-attenuation of the laser. Increased viscosity due to 4.5% v/v glycerol in the RBD nanoparticle buffer was accounted for by the UNcle Client software in Dh measurements.

### Negative stain electron microscopy

VOC-RBD-NP and VOC-HexaPro vaccines were first diluted to 75 µg/mL or 30 µg/mL in 50 mM Tris pH 7.4, 185 mM NaCl, 100 mM L-arginine, 4.5% v/v glycerol, 0.75% w/v CHAPS or 50 mM Tris pH 8, 185 mM NaCl, 0.25% w/v L-histidine, 5% v/v glycerol prior to application of 3 µL of sample onto freshly glow-discharged 300-mesh copper grids. Sample was incubated on the grid for 1 min before 6 µL of 0.75% w/v uranyl formate stain was applied to the grid. Stain was blotted off with filter paper, then the grids were dipped into another 6 µL of stain and then repeated once more. Finally, the stain was blotted away and the grids were allowed to dry for 1 min. Prepared grids were imaged in a Talos model L120C electron microscope at 57,000× or 92,000×.

### hACE2-Fc, CR3022, and LYCoV555 IgG digestion

hACE2-Fc was digested with thrombin protease (Sigma Aldrich) in the presence of 2.5 mM CaCl_2_ at a 1:300 w/w thrombin:protein ratio. The reaction was incubated at ambient temperature for 16–18 h with gentle rocking. Following incubation, the reaction mixture was concentrated using Ultracel 10 K MWCO centrifugal filters (Millipore Amicon Ultra) and sterile filtered (0.22 μM). Cleaved hACE2 monomer was separated from uncleaved hACE2-Fc and the cleaved Fc regions using Protein A purification (see Protein purification above) on a HiScreen MabSelect SuRe column (Cytiva) using an ӒKTA avant 25 FPLC (Cytiva). Cleaved hACE2 monomer was collected in the flowthrough, sterile-filtered (0.22 μm), and quantified by UV/vis.

LysC (New England BioLabs) was diluted to 10 ng/μL in 10 mM Tris pH 8 and added to CR3022 or LYCoV555 IgG at 1:2000 w/w LysC:IgG and subsequently incubated for 18 h at 37 °C with orbital shaking at 230 rpm. The cleavage reaction was concentrated using Ultracel 10 K MWCO centrifugal filters (Millipore Amicon Ultra) and sterile filtered (0.22 μM). Cleaved CR3022 or LYCoV555 mAb was separated from uncleaved CR3022 or LYCoV555 IgG and the Fc portion of cleaved IgG, using Protein A purification as described above. Cleaved CR3022 or LYCoV555 was collected in the flowthrough, sterile-filtered (0.22 μm), and quantified by UV/vis.

### Bio-layer interferometry (affinity determination)

Affinity assays were performed and analyzed using BLI on an Octet Red 96 System (Pall Forté Bio/Sartorius) at ambient temperature with shaking at 1000 rpm. VOC-RBD-I53-50A trimeric components were diluted to 40 μg/mL in Kinetics buffer (1× HEPES-EP+ (Pall Forté Bio), 0.05% nonfat milk, and 0.02% sodium azide). Monomeric hACE2, CR3022, and LYCoV555 Fab were diluted to 750 or 300 nM in Kinetics buffer and serially diluted three-fold for a final concentration of 3.1 or 1.23 nM. Reagents were applied to a black 96-well Greiner Bio-one microplate at 200 μL per well as described below. VOC-RBD-I53-50A components were immobilized onto Anti-Penta-HIS (HIS1K) biosensors per manufacturer instructions (Forté Bio) except using the following sensor incubation times. HIS1K biosensors were hydrated in water for 10 min, and were then equilibrated in Kinetics buffer for 60 s. The HIS1K tips were loaded with diluted trimeric VOC-RBD-I53-50A component or monomeric RBD for 150 s and washed with Kinetics buffer for 60 s. The association step was performed by dipping the HIS1K biosensors with immobilized immunogen into diluted hACE2 monomer, CR3022, or LYCoV555 Fab for 200 s, then dissociation was measured by inserting the biosensors back into Kinetics buffer for 200 s. The data were baseline subtracted and the plots fitted using the Pall FortéBio/Sartorius analysis software (version 12.0). Plots in Fig. 2 show the association and dissociation steps.

### ELISA (antigenicity)

For anti-HexaPro ELISA, 50 μL of 2 μg/mL HexaPro S was plated onto 384-well Nunc Maxisorp (ThermoFisher) plates in PBS and sealed overnight at RT. The next day, the plates were slapped dry and blocked with Casein (ThermoFisher) for 1 h at 37 °C. Plates were washed 4× in Tris-Buffered Saline Tween (TBST) using a plate washer (BioTek) and 1:5 serial dilutions of mAb were made in 40 μL TBST and incubated at 37 °C for 1 h. Plates were washed 4× with TBST on the BioTek plate washer, then anti-human (Invitrogen) horseradish peroxidase-conjugated antibodies were diluted 1:5,000 and 40 μL added to each well and incubated at 37 °C for 1 h. Plates were washed 4× in TBST on the BioTek plate washer and 40 μL of room temperature TMB (SeraCare) was added to every well for ∼5 min at room temperature. The reaction was quenched with the addition of 40 μL of 1 N HCl. Plates were immediately read at 450 nm on a BioTek plate reader and data plotted and fit in Prism (GraphPad) using nonlinear regression sigmoidal, 4PL, X is log(concentration) to determine EC_50_ values from curve fits. ELISA experiments were performed three separate times and representative experiments are shown.

### Hydrogen/Deuterium-exchange mass spectrometry

3 μg of VOC-RBD-I53-50A and VOC-Rpk9-RBD-I53-50A mutants were H/D exchanged in the deuteration buffer (pH* 7.5, 85% D_2_O, Cambridge Isotope Laboratories, Inc.) at 23 °C for 3, 60, 1800, and 72000 s, respectively. H/D exchanged samples were immediately mixed with an equal volume of ice-cold quench buffer (8 M urea, 200 mM tris(2-chloroethyl) phosphate (TCEP), 0.2% formic acid (FA)) to a final pH 2.5 and flash frozen in liquid nitrogen. Samples were analyzed by LC-MS on a Waters Synapt G2-Si mass spectrometer using a custom-built cooling box to maintain sample digestion and injection at 0 °C. Quenched samples were loaded over an immobilized pepsin column (2.1 × 50 mm) with a 200 μL/min flow of loading buffer (0.1% trifluoroacetic acid (TFA), 2% acetonitrile (ACN)). Peptides were trapped on a Waters CSH C18 trap cartridge (2.1 × 5 mm) and resolved over a Waters CSH C18 1 × 100 mm 1.7 μm column with a linear gradient from 3% to 40% B over 18 min (A: 2% ACN, 0.1% FA, 0.025% TFA; B: 100% ACN, 0.1% FA, flow rate of 40 μL/min). A series of washes steps were performed between all samples to minimize carryover^53^. The total deuterated control was made by collecting a pepsin digest elution, drying by speed-vacuum, and incubating in deuteration buffer for 1 h at 85 °C. Peptides were identified and updated based on our previously examined lists^20,54^ and validated using DriftScope™ (Waters). Deuterium uptake analysis was performed with HDExaminer (Sierra Analytics) and HX-Express v2^55,56^. Peaks were identified from the peptide spectra with binomial fitting applied. The deuterium uptake level was normalized relative to total deuterated control.

### Nanoscale differential scanning fluorimetry (Component and NP)

nanoDSF was used to measure the relative melting temperature (T_m_) and aggregation (T_agg_) temperatures of the individual VOC-RBD-I5350As and VOC-RBD-NPs, respectively, on an UNcle Nano-DSF (UNchained Laboratories). VOC-RBD-I5350As and VOC-RBD-NPs were diluted to 0.5 mg/mL in MAGiC Sauce (50 mM Tris pH 7.4, 185 mM NaCl, 100 mM L-arginine, 4.5% glycerol, and 0.75% w/v CHAPS) and 8.8 µL were applied to a 8.8 µL quartz capillary cassette (UNi, UNchained Laboratories). The barycentric mean (BCM) and static light scattering at 488 nm (SLS 488 nm) were taken from 20 °C to 95 °C. T_m_ and T_agg_ were approximated by analyzing when the derivative or second derivative of the line fit for BCM or SLS 488 nm, respectively, was approximately equal to zero.

### Bio-layer interferometry

Binding of hACE2-Fc to monovalent VOC-RBD-NP, mVOC-RBD-NP, and cVOD-RBD-NP was analyzed for antigenicity experiments and real-time stability studies using an Octet Red 96 System (Pall FortéBio/Sartorius) at ambient temperature with shaking at 1000 rpm. Protein samples were diluted to 100 nM in Kinetics buffer (1 × HEPES-EP+ (Pall Forté Bio), 0.05% nonfat milk, and 0.02% sodium azide). Buffer, receptor, and analyte were then applied to a black 96-well Greiner Bio-one microplate at 200 μL per well. Protein A biosensors (FortéBio/Sartorius) were first hydrated for 10 min in Kinetics buffer, then dipped into hACE2-Fc diluted to 10 μg/mL in Kinetics buffer in the immobilization step. After 150 s, the tips were transferred to Kinetics buffer for 60 s to reach a baseline. The association step was performed by dipping the loaded biosensors into the immunogens for 200 s, and subsequent dissociation was performed by dipping the biosensors back into Kinetics buffer for an additional 200 s. The data were baseline subtracted prior for plotting using the FortéBio analysis software (version 12.0).

### BALB/c mice immunizations

At 8 weeks of age, 5 female BALB/c mice per dosing group were vaccinated with a prime immunization, and 3 weeks later mice were boosted with a second vaccination (IACUC protocol 4470.01). Prior to inoculation, immunogen suspensions were gently mixed 1:1 vol/vol with AddaVax adjuvant (Invivogen, San Diego, CA) to reach a final concentration of 0.01 mg/mL antigen and 0.13125% w/v CHAPS. Mice were injected intramuscularly into the gastrocnemius muscle of each hind leg using a 27-gauge needle (BD, San Diego, CA) with 50 μL per injection site (100 μL total) of immunogen under isoflurane anesthesia. Specifically, mice underwent induction of anesthesia in an induction chamber using 3–5% isoflurane with an O2 flow rate of 1 L/min. After assessing depth of anesthesia via toe pinch, mice were transferred from induction chambers into nosecones with the same isoflurane and oxygen parameters for immunization. Euthermia was maintained throughout the procedure using circulating warm water pads. Following immunization, mice recovered from anesthesia in separate recovery chambers. To obtain sera all mice were bled 2 weeks after prime and boost immunizations. Blood was collected via submental venous puncture and rested in 1.5 mL plastic Eppendorf tubes at room temperature for 30 min to allow for coagulation. Serum was separated from red blood cells via centrifugation at 2000 g for 10 min. Complement factors in isolated serum were heat-inactivated via incubation at 56 °C for 60 min. Serum was stored at 4 °C or −80 °C until use. After the post-boost blood collection, mice were euthanized in CO_2_ chambers with secondary confirmation via cervical dislocation. BALB/c immunogenicity studies were repeated twice.

### Darwin mice immunizations

Kymab Darwin mice, equivalent to the Kymouse^31^ in the IgH locus except the immunoglobulin constant regions are also fully human, and with a fully human IgL kappa loci, were used in this study (a mix of males and females, 10 weeks of age). Five mice per dosing group were vaccinated with a prime immunization and 3 weeks later boosted with a second vaccination. Prior to inoculation, immunogen suspensions were gently mixed 1:1 vol/vol with AddaVax adjuvant (Invivogen) to reach a final concentration of 0.025 mg/mL antigen. Mice were injected intramuscularly into the tibialis muscle of each hind leg using a 30-gauge needle (BD) with 20 μL per injection site (40 μL total) of immunogen under isoflurane anesthesia. A final boost was administered intravenously (50 μL) with no adjuvant at week 7 in order to recruit circulating B cells to the spleen. Mice were sacrificed 5 days later under UK Home Office Schedule 1 (rising concentration of CO_2_) and spleen, lymph nodes, and bone marrow cryopreserved. Whole blood (0.1 mL) was collected 7.5 weeks after the first immunization. Serum was separated from hematocrit via centrifugation at 2000 g for 10 min. Serum was stored at −20 °C. All mice were maintained and all procedures carried out under United Kingdom Home Office License 70/8718 and with the approval of the Wellcome Trust Sanger Institute Animal Welfare and Ethical Review Body.

### Pseudovirus production

SARS-CoV-2 Wu-1 G614 S, B.1.351 S, P1, B.1.617.2 S, and B.1.1.259.BA.1 S pseudotyped VSV viruses were prepared as described previously^57–59^. Briefly, 293 T cells in DMEM supplemented with 10% FBS, 1% PenStrep seeded in polylysine coated 10-cm dishes were transfected with the plasmid encoding for the corresponding S glycoprotein using lipofectamine 2000 (Life Technologies) following manufacturer’s indications. One day post-transfection, cells were infected with VSV(G^∗^ΔG-luciferase) and after 2 h were washed five times with warm DMEM before adding medium supplemented with anti-VSV-G antibody (I1- mouse hybridoma supernatant, CRL- 2700, ATCC). Virus pseudotypes were harvested 18–24 h post-inoculation, clarified by centrifugation at 2500 g for 10 min, filtered through a 0.45 μm cutoff membrane, concentrated 10 times with a 30 kDa MWCO Sartorius membrane, aliquoted, and stored at −80 °C prior to use at a 1:25 dilution in DMEM.

### Pseudovirus neutralization

HEK293-hACE2 cells^60^ were cultured in DMEM with 10% FBS (Hyclone) and 1% PenStrep with 8% CO_2_ in a 37 °C incubator (ThermoFisher). One day prior to infection, 40 μL of poly-lysine (Sigma) was placed into white 96-well plates and incubated with rotation for 5 min. Poly-lysine was removed, plates were dried for 5 min then washed 1× with water prior to plating with ~40,000 cells. The following day, cells were checked to be at 80% confluence. In an empty half-area 96-well plate, a 1:3 serial dilution of sera was made in DMEM and diluted pseudovirus was then added to the serial dilution and incubated at room temperature for 30–60 min. After incubation, the sera-virus mixture was added to the cells and incubated at 37 °C for 2 hours. Following the 2 h incubation, 40 μL of 20% FBS-2% PenStrep DMEM was added for overnight incubation. After 16–20 h 40 μL/well of One-Glo-EX substrate (Promega) was added to the cells and incubated in the dark for 5–10 min prior reading on a BioTek plate reader. Measurements were done in at least duplicate with distinct pseudovirus batches and backbones. Relative luciferase units were plotted and normalized in Prism (GraphPad). Nonlinear regression of log(inhibitor) versus normalized response was used to determine IC_50_ values from curve fits.

### Quantification and statistical analysis

Statistical details of experiments can be found in the figure legends. For mouse experiments, 5 BALB/c animals were used and experiments were completed in at least duplicate. Geometric mean titers were calculated. Kruskal–Wallis tests were performed to compare two groups to determine whether they were statistically different. Significance is indicated with stars: ^∗^*p* < 0.05; ^∗∗^*p* < 0.01 and non-significant differences are not shown.

## Supplementary information

Supplementary Information

Supplementary File 1

## Data Availability

The datasets generated during and/or analyzed during the current study are available from the corresponding author upon reasonable request.

## References

[1] Novel Coronavirus – China. https://www.who.int/emergencies/disease-outbreak-news/item/2020-DON233.

[2] Corbett, K. S. et al. SARS-CoV-2 mRNA vaccine design enabled by prototype pathogen preparedness. *Nature* **586**, 567–571 (2020). 32756549 10.1038/s41586-020-2622-0 PMC7581537

[3] van Doremalen, N. et al. ChAdOx1 nCoV-19 vaccine prevents SARS-CoV-2 pneumonia in rhesus macaques. *Nature* **586**, 578–582 (2020). 32731258 10.1038/s41586-020-2608-y PMC8436420

[4] Wang, Z. et al. mRNA vaccine-elicited antibodies to SARS-CoV-2 and circulating variants. *Nature* **592**, 616–622 (2021). 33567448 10.1038/s41586-021-03324-6 PMC8503938

[5] Tegally, H. et al. Detection of a SARS-CoV-2 variant of concern in South Africa. *Nature* **592**, 438–443 (2021). 33690265 10.1038/s41586-021-03402-9

[6] Zhou, D. et al. Evidence of escape of SARS-CoV-2 variant B.1.351 from natural and vaccine-induced sera. *Cell* **184**, 2348–2361.e6 (2021). 33730597 10.1016/j.cell.2021.02.037 PMC7901269

[7] Viana, R. et al. Rapid epidemic expansion of the SARS-CoV-2 Omicron variant in Southern Africa. *Nature* **603**, 679–686 (2022). 35042229 10.1038/s41586-022-04411-y PMC8942855

[8] Lu, G. & Moriyama, E. N. 2019nCoVR-A comprehensive genomic resource for SARS-CoV-2 variant surveillance. *Innovation (Camb.)* **2**, 100150 (2021). 34401863 10.1016/j.xinn.2021.100150 PMC8357486

[9] Tracking SARS-CoV-2 variants. https://www.who.int/en/activities/tracking-SARS-CoV-2-variants.

[10] Gagne, M. et al. mRNA-1273 or mRNA-Omicron boost in vaccinated macaques elicits similar B cell expansion, neutralizing responses, and protection from Omicron. *Cell* **185**, 1556–1571.e18 (2022). 35447072 10.1016/j.cell.2022.03.038 PMC8947944

[11] Corbett, K. S. et al. Protection against SARS-CoV-2 Beta variant in mRNA-1273 vaccine-boosted nonhuman primates. *Science* **374**, 1343–1353 (2021). 34672695 10.1126/science.abl8912

[12] Walls, A. C. et al. Elicitation of potent neutralizing antibody responses by designed protein nanoparticle vaccines for SARS-CoV-2. *Cell* **183**, 1367–1382.e17 (2020). 33160446 10.1016/j.cell.2020.10.043 PMC7604136

[13] Arunachalam, P. S. et al. Adjuvanting a subunit COVID-19 vaccine to induce protective immunity. *Nature* **594**, 253–258 (2021). 33873199 10.1038/s41586-021-03530-2

[14] Song, J. Y. et al. Immunogenicity and safety of SARS-CoV-2 recombinant protein nanoparticle vaccine GBP510 adjuvanted with AS03: interim results of a randomised, active-controlled, observer-blinded, phase 3 trial. *eClinicalMedicine* **64**, 102140 (2023). 37711219 10.1016/j.eclinm.2023.102140 PMC10498190

[15] Song, J. Y. et al. Safety and immunogenicity of a SARS-CoV-2 recombinant protein nanoparticle vaccine (GBP510) adjuvanted with AS03: A randomised, placebo-controlled, observer-blinded phase 1/2 trial. *EClinicalMedicine* **51**, 101569 (2022). 35879941 10.1016/j.eclinm.2022.101569 PMC9304916

[16] Walls, A. C. et al. Elicitation of broadly protective sarbecovirus immunity by receptor-binding domain nanoparticle vaccines. *Cell* **184**, 5432–5447.e16 (2021). 34619077 10.1016/j.cell.2021.09.015 PMC8440233

[17] Cohen, A. A. et al. Mosaic RBD nanoparticles protect against challenge by diverse sarbecoviruses in animal models. *Science* **377**, eabq0839 (2022). 35857620 10.1126/science.abq0839 PMC9273039

[18] Cohen, A. A. et al. Mosaic nanoparticles elicit cross-reactive immune responses to zoonotic coronaviruses in mice. *Science* **371**, 735–741 (2021). 33436524 10.1126/science.abf6840 PMC7928838

[19] Hsieh, C.-L. et al. Structure-based design of prefusion-stabilized SARS-CoV-2 spikes. *Science* **369**, 1501–1505 (2020). 32703906 10.1126/science.abd0826 PMC7402631

[20] Ellis, D. et al. Stabilization of the SARS-CoV-2 spike receptor-binding domain using deep mutational scanning and structure-based design. *Front. Immunol.* **12**, 710263 (2021). 34267764 10.3389/fimmu.2021.710263 PMC8276696

[21] Bale, J. B. et al. Accurate design of megadalton-scale two-component icosahedral protein complexes. *Science* **353**, 389–394 (2016). 27463675 10.1126/science.aaf8818 PMC5485857

[22] Starr, T. N., Greaney, A. J., Dingens, A. S. & Bloom, J. D. Complete map of SARS-CoV-2 RBD mutations that escape the monoclonal antibody LY-CoV555 and its cocktail with LY-CoV016. *Cell Rep. Med.* **2**, 100255 (2021). 33842902 10.1016/j.xcrm.2021.100255 PMC8020059

[23] Yuan, M. et al. A highly conserved cryptic epitope in the receptor binding domains of SARS-CoV-2 and SARS-CoV. *Science* **368**, 630–633 (2020). 32245784 10.1126/science.abb7269 PMC7164391

[24] Jones, B. E. et al. The neutralizing antibody, LY-CoV555, protects against SARS-CoV-2 infection in nonhuman primates. *Sci. Transl. Med.* **13**, eabf1906 (2021). 33820835 10.1126/scitranslmed.abf1906 PMC8284311

[25] ter Meulen, J. et al. Human monoclonal antibody combination against SARS coronavirus: synergy and coverage of escape mutants. *PLoS Med.* **3**, e237 (2006). 16796401 10.1371/journal.pmed.0030237 PMC1483912

[26] McCallum, M. et al. N-terminal domain antigenic mapping reveals a site of vulnerability for SARS-CoV-2. *Cell* **184**, 2332–2347.e16 (2021). 33761326 10.1016/j.cell.2021.03.028 PMC7962585

[27] Piccoli, L. et al. Mapping neutralizing and immunodominant sites on the SARS-CoV-2 spike receptor-binding domain by structure-guided high-resolution serology. *Cell* **183**, 1024–1042.e21 (2020). 32991844 10.1016/j.cell.2020.09.037 PMC7494283

[28] McCallum, M. et al. Structural basis of SARS-CoV-2 Omicron immune evasion and receptor engagement. *Science* **375**, 864–868 (2022). 35076256 10.1126/science.abn8652 PMC9427005

[29] Liu, L. et al. Potent neutralizing antibodies against multiple epitopes on SARS-CoV-2 spike. *Nature* **584**, 450–456 (2020). 32698192 10.1038/s41586-020-2571-7

[30] Cerutti, G. et al. Potent SARS-CoV-2 neutralizing antibodies directed against spike N-terminal domain target a single supersite. *Cell Host Microbe* **29**, 819–833.e7 (2021). 33789084 10.1016/j.chom.2021.03.005 PMC7953435

[31] Richardson, E. et al. Characterisation of the immune repertoire of a humanised transgenic mouse through immunophenotyping and high-throughput sequencing. *Elife* **12**, e81629 (2023). 36971345 10.7554/eLife.81629 PMC10115447

[32] Greaney, A. J. et al. Comprehensive mapping of mutations in the SARS-CoV-2 receptor-binding domain that affect recognition by polyclonal human plasma antibodies. *Cell Host Microbe* **29**, 463–476.e6 (2021). 33592168 10.1016/j.chom.2021.02.003 PMC7869748

[33] Walls, A. C. et al. Distinct sensitivities to SARS-CoV-2 variants in vaccinated humans and mice. *Cell Rep.* **40**, 111299 (2022). 35988541 10.1016/j.celrep.2022.111299 PMC9376299

[34] Arunachalam, P. S. et al. Durable protection against the SARS-CoV-2 Omicron variant is induced by an adjuvanted subunit vaccine. *Sci. Transl. Med.* **14**, eabq4130 (2022). 35976993 10.1126/scitranslmed.abq4130 PMC10466502

[35] Feng, Y. et al. Broadly neutralizing antibodies against sarbecoviruses generated by immunization of macaques with an AS03-adjuvanted COVID-19 vaccine. *Sci. Transl. Med.* **15**, eadg7404 (2023). 37163615 10.1126/scitranslmed.adg7404 PMC11032722

[36] Pajon, R. et al. SARS-CoV-2 Omicron variant neutralization after mRNA-1273 booster vaccination. *N. Engl. J. Med.* **386**, 1088–1091 (2022). 35081298 10.1056/NEJMc2119912 PMC8809504

[37] Gobeil, S. M.-C. et al. Effect of natural mutations of SARS-CoV-2 on spike structure, conformation, and antigenicity. *Science* **373**, eabi6226 (2021). 34168071 10.1126/science.abi6226 PMC8611377

[38] Leonard, A. C. et al. Stabilization of the SARS-CoV-2 receptor binding domain by protein core redesign and deep mutational scanning. *Protein Eng. Des. Sel.* **35**, gzac002 (2022). 35325236 10.1093/protein/gzac002 PMC9077414

[39] Ahmed, S. et al. A stabilized, monomeric, receptor binding domain elicits high-titer neutralizing antibodies against all SARS-CoV-2 variants of concern. *Front. Immunol.* **12**, 765211 (2021). 34956193 10.3389/fimmu.2021.765211 PMC8695907

[40] Starr, T. N. et al. Deep mutational scanning of SARS-CoV-2 receptor binding domain reveals constraints on folding and ACE2 binding. *Cell* **182**, 1295–1310.e20 (2020). 32841599 10.1016/j.cell.2020.08.012 PMC7418704

[41] Starr, T. N. et al. Deep mutational scans for ACE2 binding, RBD expression, and antibody escape in the SARS-CoV-2 Omicron BA.1 and BA.2 receptor-binding domains. *PLoS Pathog.* **18**, e1010951 (2022). 36399443 10.1371/journal.ppat.1010951 PMC9674177

[42] Cao, Y. et al. Imprinted SARS-CoV-2 humoral immunity induces convergent Omicron RBD evolution. *Nature* **614**, 521–529 (2023). 36535326 10.1038/s41586-022-05644-7 PMC9931576

[43] Smith, D. J. et al. Mapping the antigenic and genetic evolution of influenza virus. *Science* **305**, 371–376 (2004). 15218094 10.1126/science.1097211

[44] Chalkias, S. et al. Original SARS-CoV-2 monovalent and Omicron BA.4/BA.5 bivalent COVID-19 mRNA vaccines: phase 2/3 trial interim results. *Nat. Med.* **29**, 2325–2333 (2023). 37653342 10.1038/s41591-023-02517-y PMC10504066

[45] Chalkias, S. et al. Three-month antibody persistence of a bivalent Omicron-containing booster vaccine against COVID-19. *Nat. Commun.* **14**, 5125 (2023). 37612300 10.1038/s41467-023-38892-w PMC10447540

[46] Usdan, L. et al. A bivalent Omicron-BA.4/BA.5-Adapted BNT162b2 booster in ≥12-year-olds. *Clin. Infect. Dis*. 10.1093/cid/ciad718 (2023). 10.1093/cid/ciad718 PMC11093671 38016021

[47] Mu, Z. et al. mRNA-encoded HIV-1 Env trimer ferritin nanoparticles induce monoclonal antibodies that neutralize heterologous HIV-1 isolates in mice. *Cell Rep.* **38**, 110514 (2022). 35294883 10.1016/j.celrep.2022.110514 PMC8922439

[48] Kanekiyo, M. et al. Mosaic nanoparticle display of diverse influenza virus hemagglutinins elicits broad B cell responses. *Nat. Immunol.* **20**, 362–372 (2019). 30742080 10.1038/s41590-018-0305-x PMC6380945

[49] Boyoglu-Barnum, S. et al. Quadrivalent influenza nanoparticle vaccines induce broad protection. *Nature* **592**, 623–628 (2021). 33762730 10.1038/s41586-021-03365-x PMC8269962

[50] Cohen, A. A. et al. Construction, characterization, and immunization of nanoparticles that display a diverse array of influenza HA trimers. *PLoS One* **16**, e0247963 (2021). 33661993 10.1371/journal.pone.0247963 PMC7932532

[51] CEPI. CEPI expands partnership with SK bioscience to develop a ‘variant-proof’ vaccine against SARS-CoV and SARS-CoV-2 variants –. *CEPI* https://cepi.net/news_cepi/cepi-expands-partnership-with-sk-bioscience-to-develop-a-variant-proof-vaccine-against-sars-cov-and-sars-cov-2-variants/ (2021).

[52] Lempp, F. A. et al. Lectins enhance SARS-CoV-2 infection and influence neutralizing antibodies. *Nature*10.1038/s41586-021-03925-1 (2021). 10.1038/s41586-021-03925-1 34464958

[53] Verkerke, H. P. et al. Epitope-independent purification of native-like envelope trimers from diverse HIV-1 isolates. *J. Virol.* **90**, 9471–9482 (2016). 27512064 10.1128/JVI.01351-16 PMC5044855

[54] Chen, C. et al. hACE2-induced allosteric activation in SARS-CoV versus SARS-CoV-2 spike assemblies revealed by structural dynamics. *ACS Infect. Dis.* **9**, 1180–1189 (2023). 37166130 10.1021/acsinfecdis.3c00010 PMC10228703

[55] Guttman, M., Weis, D. D., Engen, J. R. & Lee, K. K. Analysis of overlapped and noisy hydrogen/deuterium exchange mass spectra. *J. Am. Soc. Mass Spectrom.* **24**, 1906–1912 (2013). 24018862 10.1007/s13361-013-0727-5 PMC3855366

[56] Weis, D. D., Engen, J. R. & Kass, I. J. Semi-automated data processing of hydrogen exchange mass spectra using HX-Express. *J. Am. Soc. Mass Spectrom.* **17**, 1700–1703 (2006). 16931036 10.1016/j.jasms.2006.07.025

[57] Sauer, M. M. et al. Structural basis for broad coronavirus neutralization. *Nat. Struct. Mol. Biol.* **28**, 478–486 (2021). 33981021 10.1038/s41594-021-00596-4

[58] Walls, A. C. et al. SARS-CoV-2 breakthrough infections elicit potent, broad, and durable neutralizing antibody responses. *Cell* **185**, 872–880.e3 (2022). 35123650 10.1016/j.cell.2022.01.011 PMC8769922

[59] McCallum, M. et al. Molecular basis of immune evasion by the Delta and Kappa SARS-CoV-2 variants. *Science* **374**, 1621–1626 (2021). 34751595 10.1126/science.abl8506 PMC12240541

[60] Crawford, K. H. D. et al. Protocol and reagents for pseudotyping lentiviral particles with SARS-CoV-2 spike protein for neutralization assays. *Viruses* **12**, 513 (2020). 32384820 10.3390/v12050513 PMC7291041

[61] Meng, E. C. et al. UCSF ChimeraX: tools for structure building and analysis. *Protein Sci.* **32**, e4792 (2023). 37774136 10.1002/pro.4792 PMC10588335

