# Supplementary material for: Potent neutralization of SARS-CoV-2 variants by RBD nanoparticle and prefusion-stabilized spike immunogens
Source: NPJ Vaccines. 2024 Oct 8;9:184. doi: 10.1038/s41541-024-00982-1 (PMC11461925; doi:10.1038/s41541-024-00982-1)
Supplement: Supplementary file 1 — Supplementary Information [file 41541_2024_982_MOESM1_ESM.pdf]

Supplementary Information

Supplementary Table 1: Amino acid sequences.

Construct Name	Amino Acid Sequence
**Wu-1-RBD-I53-50A**	MGILPSPGMPALLSLVSLLSVLLMGCVAETGTRFPNITNLCPFGEVFNATRFASVYAWNRKRISNCVADYSV<br>LYNSASFSTFKCYGVSPTKLNDLCFTNVYADSFVIRGDEVRQIAPGQTGKIADYNYKLPDDFTGCVIAWNSN<br>NLDSKVGGNYNYLYRLFRKSNLKPFERDISTEIYQAGSTPCNGVEGFNCYFPLQSYGFQPTNGVGYQPYR<br>VVVLSFELLHAPATVCGPKKSTGGSGGSGSGGSGGSGSEKAAKAEEAARKMEELFKKHKIVAVLRANSVE<br>EAIEKAVAVFAGGVHLIEITFTVPDADTVIKALSVLKEKGAIIGAGTVTSVEQARKAVESGAEFIVSPHLDEEIS<br>QFAKEKGVFYMPGVMTPTELVKAMKLGHTILKLFPGEVVGPQFVKAMKGPFPNVKFVPTGGVNLDNVAEW<br>FKAGVLAVGVGSALVKGTPDEVREKAKAFVEKIRGATEGGSHHHHHHHH*
**Wu-1Rpk9-RBD-I53-50A**	MGILPSPGMPALLSLVSLLSVLLMGCVAETGTRFPNITNLCPFGEVFNATRFASVYAWNRKRISNCVADFSV<br>LYNSASFSTFKCYGVSPTKLNDLCWTNIYADSFVIRGDEVRQIAPGQTGKIADYNYKLPDDFTGCVIAWNSN<br>NLDSKVGGNYNYLYRLFRKSNLKPFERDISTEIYQAGSTPCNGVEGFNCYFPLQSYGFQPTNGVGYQPYR<br>VVVLSFELLHAPATVCGPKKSTGGSGGSGSGGSGGSGSEKAAKAEEAARKMEELFKKHKIVAVLRANSVE<br>EAIEKAVAVFAGGVHLIEITFTVPDADTVIKALSVLKEKGAIIGAGTVTSVEQARKAVESGAEFIVSPHLDEEIS<br>QFAKEKGVFYMPGVMTPTELVKAMKLGHTILKLFPGEVVGPQFVKAMKGPFPNVKFVPTGGVNLDNVAEW<br>FKAGVLAVGVGSALVKGTPDEVREKAKAFVEKIRGATEGGSHHHHHHHH*
**β-RBD-I53-50A**	MGILPSPGMPALLSLVSLLSVLLMGCVAETGTRFPNITNLCPFGEVFNATRFASVYAWNRKRISNCVADYSV<br>LYNSASFSTFKCYGVSPTKLNDLCFTNVYADSFVIRGDEVRQIAPGQTGNIADYNYKLPDDFTGCVIAWNSN<br>NLDSKVGGNYNYLYRLFRKSNLKPFERDISTEIYQAGSTPCNGVKGFNCYFPLQSYGFQPTYGVGYQPYR<br>VVVLSFELLHAPATVCGPKKSTGGSGGSGSGGSGGSGSEKAAKAEEAARKMEELFKKHKIVAVLRANSVE<br>EAIEKAVAVFAGGVHLIEITFTVPDADTVIKALSVLKEKGAIIGAGTVTSVEQARKAVESGAEFIVSPHLDEEIS<br>QFAKEKGVFYMPGVMTPTELVKAMKLGHTILKLFPGEVVGPQFVKAMKGPFPNVKFVPTGGVNLDNVAEW<br>FKAGVLAVGVGSALVKGTPDEVREKAKAFVEKIRGATEGGSHHHHHHHH*
**βRpk9-RBD-I53-50A**	MGILPSPGMPALLSLVSLLSVLLMGCVAETGTRFPNITNLCPFGEVFNATRFASVYAWNRKRISNCVADFSV<br>LYNSASFSTFKCYGVSPTKLNDLCWTNIYADSFVIRGDEVRQIAPGQTGNIADYNYKLPDDFTGCVIAWNSN<br>NLDSKVGGNYNYLYRLFRKSNLKPFERDISTEIYQAGSTPCNGVKGFNCYFPLQSYGFQPTYGVGYQPYR<br>VVVLSFELLHAPATVCGPKKSTGGSGGSGSGGSGGSGSEKAAKAEEAARKMEELFKKHKIVAVLRANSVE<br>EAIEKAVAVFAGGVHLIEITFTVPDADTVIKALSVLKEKGAIIGAGTVTSVEQARKAVESGAEFIVSPHLDEEIS<br>QFAKEKGVFYMPGVMTPTELVKAMKLGHTILKLFPGEVVGPQFVKAMKGPFPNVKFVPTGGVNLDNVAEW<br>FKAGVLAVGVGSALVKGTPDEVREKAKAFVEKIRGATEGGSHHHHHHHH*
**γ-RBD-I53-50A**	MGILPSPGMPALLSLVSLLSVLLMGCVAETGTRFPNITNLCPFGEVFNATRFASVYAWNRKRISNCVADYSV<br>LYNSASFSTFKCYGVSPTKLNDLCFTNVYADSFVIRGDEVRQIAPGQTGTIADYNYKLPDDFTGCVIAWNSN<br>NLDSKVGGNYNYLYRLFRKSNLKPFERDISTEIYQAGSTPCNGVKGFNCYFPLQSYGFQPTYGVGYQPYR<br>VVVLSFELLHAPATVCGPKKSTGGSGGSGSGGSGGSGSEKAAKAEEAARKMEELFKKHKIVAVLRANSVE<br>EAIEKAVAVFAGGVHLIEITFTVPDADTVIKALSVLKEKGAIIGAGTVTSVEQARKAVESGAEFIVSPHLDEEIS<br>QFAKEKGVFYMPGVMTPTELVKAMKLGHTILKLFPGEVVGPQFVKAMKGPFPNVKFVPTGGVNLDNVAEW<br>FKAGVLAVGVGSALVKGTPDEVREKAKAFVEKIRGATEGGSHHHHHHHH*
**γRpk9-RBD-I53-50A**	MGILPSPGMPALLSLVSLLSVLLMGCVAETGTRFPNITNLCPFGEVFNATRFASVYAWNRKRISNCVADFSV<br>LYNSASFSTFKCYGVSPTKLNDLCWTNIYADSFVIRGDEVRQIAPGQTGTIADYNYKLPDDFTGCVIAWNSN<br>NLDSKVGGNYNYLYRLFRKSNLKPFERDISTEIYQAGSTPCNGVKGFNCYFPLQSYGFQPTYGVGYQPYR<br>VVVLSFELLHAPATVCGPKKSTGGSGGSGSGGSGGSGSEKAAKAEEAARKMEELFKKHKIVAVLRANSVE<br>EAIEKAVAVFAGGVHLIEITFTVPDADTVIKALSVLKEKGAIIGAGTVTSVEQARKAVESGAEFIVSPHLDEEIS<br>QFAKEKGVFYMPGVMTPTELVKAMKLGHTILKLFPGEVVGPQFVKAMKGPFPNVKFVPTGGVNLDNVAEW<br>FKAGVLAVGVGSALVKGTPDEVREKAKAFVEKIRGATEGGSHHHHHHHH*
**E484K-RBD-I53-50A**	MGILPSPGMPALLSLVSLLSVLLMGCVAETGTRFPNITNLCPFGEVFNATRFASVYAWNRKRISNCVADYSV<br>LYNSASFSTFKCYGVSPTKLNDLCFTNVYADSFVIRGDEVRQIAPGQTGKIADYNYKLPDDFTGCVIAWNSN<br>NLDSKVGGNYNYLYRLFRKSNLKPFERDISTEIYQAGSTPCNGVKGFNCYFPLQSYGFQPTNGVGYQPYR<br>VVVLSFELLHAPATVCGPKKSTGGSGGSGSGGSGGSGSEKAAKAEEAARKMEELFKKHKIVAVLRANSVE<br>EAIEKAVAVFAGGVHLIEITFTVPDADTVIKALSVLKEKGAIIGAGTVTSVEQARKAVESGAEFIVSPHLDEEIS<br>QFAKEKGVFYMPGVMTPTELVKAMKLGHTILKLFPGEVVGPQFVKAMKGPFPNVKFVPTGGVNLDNVAEW

	FKAGVLAVGVGSALVKGTPDEVREKAKAFVEKIRGATEGGSHHHHHHHH*
**E484KRpk9-RBD-I53-50A**	MGILPSPGMPALLSLVSLLSVLLMGCVAETGTRFPNITNLCPFGEVFNATRFASVYAWNRKRISNCVADFSV<br>LYNSASFSTFKCYGVSPTKLNDLCWTNIYADSFVIRGDEVRQIAPGQTGKIADYNYKLPDDFTGCVIAWNSN<br>NLDSKVGGNYNYLYRLFRKSNLKPFERDISTEIYQAGSTPCNGVKGFNCYFPLQSYGFQPTNGVGYQPYR<br>VVVLSFELLHAPATVCGPKKSTGGSGGSGSGGSGGSGSEKAAKAEEAARKMEELFKKHKIVAVLRANSVE<br>EAIEKAVAVFAGGVHLIEITFTVPDADTVIKALSVLKEKGAIIGAGTVTSVEQARKAVESGAEFIVSPHLDEEIS<br>QFAKEKGVFYMPGVMTPTELVKAMKLGHTILKLFPGEVVGPQFVKAMKGPFPNVKFVPTGGVNLDNVAEW<br>FKAGVLAVGVGSALVKGTPDEVREKAKAFVEKIRGATEGGSHHHHHHHH*
**OmicronBA.4/5Rpk9-RBD-I53-50A**	MGILPSPGMPALLSLVSLLSVLLMGCVAETGTRFPNITNLCPFDEVFNATRFASVYAWNRKRISNCVADFSVL<br>YNFAPFFAFKCYGVSPTKLNDLCWTNIYADSFVIRGNEVSQIAPGQTGNIADYNYKLPDDFTGCVIAWNSNK<br>LDSKVGGNYNYRYRLFRKSNLKPFERDISTEIYQAGNKPCNGVAGVNCYFPLQSYGFRPTYGVGHQPYRV<br>VVLSFELLHAPATVCGPKKSTGGSGGSGSGGSGGSGSEKAAKAEEAARKMEELFKKHKIVAVLRANSVEE<br>AIEKAVAVFAGGVHLIEITFTVPDADTVIKALSVLKEKGAIIGAGTVTSVEQARKAVESGAEFIVSPHLDEEISQ<br>FAKEKGVFYMPGVMTPTELVKAMKLGHTILKLFPGEVVGPQFVKAMKGPFPNVKFVPTGGVNLDNVAEWF<br>KAGVLAVGVGSALVKGTPDEVREKAKAFVEKIRGATEGGSHHHHHH*
**OmicronXBB.1.5-RBD-I53-50A**	MGILPSPGMPALLSLVSLLSVLLMGCVAETGTRFPNITNLCPFHEVFNATTFASVYAWNRKRISNCVADYSVI<br>YNFAPFFAFKCYGVSPTKLNDLCFTNVYADSFVIRGNEVSQIAPGQTGNIADYNYKLPDDFTGCVIAWNSNK<br>LDSKPSGNYNYLYRLFRKSKLKPFERDISTEIYQAGNKPCNGVAGPNCYSPLQSYGFRPTYGVGHQPYRV<br>VVLSFELLHAPATVCGPKKSTGGSGGSGSGGSGGSGSEKAAKAEEAARKMEELFKKHKIVAVLRANSVEE<br>AIEKAVAVFAGGVHLIEITFTVPDADTVIKALSVLKEKGAIIGAGTVTSVEQARKAVESGAEFIVSPHLDEEISQ<br>FAKEKGVFYMPGVMTPTELVKAMKLGHTILKLFPGEVVGPQFVKAMKGPFPNVKFVPTGGVNLDNVAEWF<br>KAGVLAVGVGSALVKGTPDEVREKAKAFVEKIRGATEGGSHHHHHHHH*
**Wu-1-HexaPro**	MFVFLVLLPLVSSQCVNLTTRTQLPPAYTNSFTRGVYYPDKVFRSSVLHSTQDLFLPFFSNVTWFHAIHVSG<br>TNGTKRFDNPVLPFNDGVYFASTEKSNIIRGWIFGTTLDSKTQSLLIVNNATNVVIKVCEFQFCNDPFLGVYY<br>HKNNKSWMESEFRVYSSANNCTFEYVSQPFLMDLEGKQGNFKNLREFVFKNIDGYFKIYSKHTPINLVRDL<br>PQGFSALEPLVDLPIGINITRFQTLLALHRSYLTPGDSSSGWTAGAAAYYVGYLQPRTFLLKYNENGTITDAV<br>DCALDPLSETKCTLKSFTVEKGIYQTSNFRVQPTESIVRFPNITNLCPFGEVFNATRFASVYAWNRKRISNCV<br>ADYSVLYNSASFSTFKCYGVSPTKLNDLCFTNVYADSFVIRGDEVRQIAPGQTGKIADYNYKLPDDFTGCVI<br>AWNSNNLDSKVGGNYNYLYRLFRKSNLKPFERDISTEIYQAGSTPCNGVEGFNCYFPLQSYGFQPTNGVG<br>YQPYRVVVLSFELLHAPATVCGPKKSTNLVKNKCVNFNFNGLTGTGVLTESNKKFLPFQQFGRDIADTTDAV<br>RDPQTLEILDITPCSFGGVSVITPGTNTSNQVAVLYQDVNCTEVPVAIHADQLTPTWRVYSTGSNVFQTRAG<br>CLIGAEHVNNSYECDIPIGAGICASYQTQTNSPGSASSVASQSIIAYTMSLGAENSVAYSNNSIAIPTNFTISVT<br>TEILPVSMTKTSVDCTMYICGDSTECSNLLLQYGSFCTQLNRALTGIAVEQDKNTQEVFAQVKQIYKTPPIKD<br>FGGFNFSQILPDPSKPSKRSPIEDLLFNKVTLADAGFIKQYGDCLGDIAARDLICAQKFNGLTVLPPLLTDEMI<br>AQYTSALLAGTITSGWTFGAGPALQIPFPMQMAYRFNGIGVTQNVLYENQKLIANQFNSAIGKIQDSLSSTP<br>SALGKLQDVVNQNAQALNTLVKQLSSNFGAISSVLNDILSRLDPPEAEVQIDRLITGRLQSLQTYVTQQLIRA<br>AEIRASANLAATKMSECVLGQSKRVDFCGKGYHLMSFPQSAPHGVVFLHVTYVPAQEKNFTTAPAICHDGK<br>AHFPREGVFVSNGTHWFVTQRNFYEPQIITTDNTFVSGNCDVVIGIVNNTVYDPLQPELDSFKEELDKYFKN<br>HTSPDVDLGDISGINASVVNIQKEIDRLNEVAKNLNESLIDLQELGKYEQGSGYIPEAPRDGQAYVRKDGEW<br>VLLSTFLGRSLEVLFQGPGHHHHHHHHSAWSHPQFEKGGGSGGGGSGGSAWSHPQFEK*
**β-HexaPro**	MARAWIFFLLCLAGRALAQCVNFTTRTQLPPAYTNSFTRGVYYPDKVFRSSVLHSTQDLFLPFFSNVTWFH<br>AIHVSGTNGTKRFANPVLPFNDGVYFASTEKSNIIRGWIFGTTLDSKTQSLLIVNNATNVVIKVCEFQFCNDP<br>FLGVYYHKNNKSWMESEFRVYSSANNCTFEYVSQPFLMDLEGKQGNFKNLREFVFKNIDGYFKIYSKHTPI<br>NLVRGLPQGFSALEPLVDLPIGINITRFQTLHISYLTPGDSSSGWTAGAAAYYVGYLQPRTFLLKYNENGTIT<br>DAVDCALDPLSETKCTLKSFTVEKGIYQTSNFRVQPTESIVRFPNITNLCPFGEVFNATRFASVYAWNRKRIS<br>NCVADYSVLYNSASFSTFKCYGVSPTKLNDLCFTNVYADSFVIRGDEVRQIAPGQTGNIADYNYKLPDDFTG<br>CVIAWNSNNLDSKVGGNYNYLYRLFRKSNLKPFERDISTEIYQAGSTPCNGVKGFNCYFPLQSYGFQPTYG<br>VGYQPYRVVVLSFELLHAPATVCGPKKSTNLVKNKCVNFNFNGLTGTGVLTESNKKFLPFQQFGRDIADTT<br>DAVRDPQTLEILDITPCSFGGVSVITPGTNTSNQVAVLYQGVNCTEVPVAIHADQLTPTWRVYSTGSNVFQT<br>RAGCLIGAEHVNNSYECDIPIGAGICASYQTQTNSPGSASSVASQSIIAYTMSLGVENSVAYSNNSIAIPTNFT<br>ISVTTEILPVSMTKTSVDCTMYICGDSTECSNLLLQYGSFCTQLNRALTGIAVEQDKNTQEVFAQVKQIYKTP<br>PIKDFGGFNFSQILPDPSKPSKRSPIEDLLFNKVTLADAGFIKQYGDCLGDIAARDLICAQKFNGLTVLPPLLT<br>DEMIAQYTSALLAGTITSGWTFGAGPALQIPFPMQMAYRFNGIGVTQNVLYENQKLIANQFNSAIGKIQDSLS<br>STPSALGKLQDVVNQNAQALNTLVKQLSSNFGAISSVLNDILSRLDPPEAEVQIDRLITGRLQSLQTYVTQQL<br>IRAAEIRASANLAATKMSECVLGQSKRVDFCGKGYHLMSFPQSAPHGVVFLHVTYVPAQEKNFTTAPAICH<br>DGKAHFPREGVFVSNGTHWFVTQRNFYEPQIITTDNTFVSGNCDVVIGIVNNTVYDPLQPELDSFKEELDK<br>YFKNHTSPDVDLGDISGINASVVNIQKEIDRLNEVAKNLNESLIDLQELGKYEQGSGYIPEAPRDGQAYVRK

	DGEWVLLSTFLGRSLEVLFQGPGSGGLNDIFEAQKIEWHEGSGHHHHHHHH*
**γ-HexaPro**	MARAWIFFLLCLAGRALAQCVNFTNRTQLPSAYTNSFTRGVYYPDKVFRSSVLHSTQDLFLPFFSNVTWFHAIHVSGTNGTKRFDNPVLPFNDGVYFASTEKSNIIRGWIFGTTLDSKTQSLLIVNNATNVVIKVCEFQFCNYPFLGVYYHKNNKSWMESEFRVYSSANNCTFEYVSQPFLMDLEGKQGNFKNLSEFVFKNIDGYFKIYSKHTPINLVRDLPQGFSALEPLVDLPIGINITRFQTLLALHRSYLTPGDSSSGWTAGAAAYYVGYLQPRTFLLKYNENGTITDAVDCALDPLSETKCTLKSFTVEKGIYQTSNFRVQPTESIVRFPNITNLCPFGEVFNATRFASVYAWNRKRISNCVADYSVLYNSASFSTFKCYGVSPTKLNDLCFTNVYADSFVIRGDEVRQIAPGQTGTIADYNYKLPDDFTGCVIAWNSNNLDSKVGGNYNYLYRLFRKSNLKPFERDISTEIYQAGSTPCNGVKGFNCYFPLQSYGFQPTYGVGYQPYRVVVLSFELLHAPATVCGPKKSTNLVKNKCVNFNFNGLTGTGVLTESNKKFLPFQQFGRDIADTTDAVRDPQTLEILDITPCSFGGVSVITPGTNTSNQVAVLYQGVNCTEVPVAIHADQLTPTWRVYSTGSNVFQTRAGCLIGAEYVNNSYECDIPIGAGICASYQTQTNSPGSASSVASQSIIAYTMSLGAENSVAYSNNSIAIPTNFTISVTTEILPVSMTKTSVDCTMYICGDSTECSNLLLQYGSFCTQLNRALTGIAVEQDKNTQEVFAQVKQIYKTPPIKDFGGFNFSQILPDPSKPSKRSPIEDLLFNKVTLADAGFIKQYGDCLGDIAARDLICAQKFNGLTVLPPLLTDEMIAQYTSALLAGTITSGWTFGAGPALQIPFPMQMAYRFNGIGVTQNVLYENQKLIANQFNSAIGKIQDSLSSTPSALGKLQDVVNQNAQALNTLVKQLSSNFGAISSVLNDILSRLDPPEAEVQIDRLITGRLQSLQTYVTQQLIRAAEIRASANLAAIKMSECVLGQSKRVDFCGKGYHLMSFPQSAPHGVVFLHVTYVPAQEKNFTTAPAICHDGKAHFPREGVFVSNGTHWFVTQRNFYEPQIITTDNTFVSGNCDVVIGIVNNTVYDPLQPELDSFKEELDKYFKNHTSPDVDLGDISGINASVVNIQKEIDRLNEVAKNLNESLIDLQELGKYEQGSGYIPEAPRDGQAYVRKDGEWVLLSTFLGRSLEVLFQGPGSGGLNDIFEAQKIEWHEGSGHHHHHHHH*
**I53-50B.4PT1**	HMNQHSHKDHETVRIAVVRARWHAEIVDACVSAFEAAMRDIGGDRFAVDVFDVPGAYEIPLHARTLAETGRYGAVLGTAFVVNGGIYRHEFVASAVINGMMNVQLNTGVPVLSAVLTPHNYDKSKAHTLLFLALFAVKGMEAARACVEILAAREKIAA*LE
**hACE2-Fc**	MARAWIFFLLCLAGRALASTIEEQAKTFLDKFNHEAEDLFYQSSLASWNYNTNITEENVQNMNNAGDKWSAFLKEQSTLAQMYPLQEIQNLTVKLQLQALQQNGSSVLSEDKSKRLNTILNTMSTIYSTGKVCNPDNPQECLLLEPGLNEIMANSLDYNERLWAWESWRSEVGKQLRPLYEEYVVLKNEMARANHYEDYGDYWRGDYEVNGVDGYDYSRGQLIEDVEHTFEEIKPLYEHLHAYVRAKLMNAYPSYISPIGCLPAHLLGDMWGRFWTNLYSLTVPFGQKPNIDVTDAMVDQAWDAQRIFKEAEKFFVSVGLPNMTQGFWENSMLTDPGNVQKAVCHPTAWDLGKGDFRILMCTKVTMDDFLTAHHEMGHIQYDMAYAAQPFLLRNGANEGFHEAVGEIMSLSAATPKHLKSIGLLSPDFQEDNETEINFLLKQALTIVGTLPFTYMLEKWRWMVFKGEIPKDQWMKKWWEMKREIVGVVEPVPHDETYCDPASLFHVSNDYSFIRYYTRTLYQFQFQEALCQAAKHEGPLHKCDISNSTEAGQKLFNMLRLGKSEPWTLALENVVGAKNMNVRPLLNYFEPLFTWLKDQNKNSFVGWSTDWSPYADPLVPRGSGGGGDPEPKSCDKTHTCPPCPAPELLGGPSVFLFPPKPKDTLMISRTPEVTCVVVDVSHEDPEVKFNWYVDGVEVHNAKTKPREEQYNSTYRVVSVLTVLHQDWLNGKEYKCKVSNKALPAPIEKTISKAKGQPREPQVYTLPPSRDELTKNQVSLTCLVKGFYPSDIAVEWESNGQPENNYKTTPPVLDSDGSFFLYSKLTVDKSRWQQGNVFSCSVMHEALHNHYTQKSLSLSPGK*

Supplementary Table 2: RBD-NP immunogens in shelf-life stability study (Figure 4) & immunogenicity studies (Figure 5).

Immunogen name	Figure	Immunogen composition
**Wu-1-RBD-NP**	Figure 4, 5A, 5B, 5E, 5F	Wu-1-RBD-I53-50A + I53-50B.4PT1
**Wu-1Rpk9-RBD-NP**	Figure 5B	Wu-1Rpk9-RBD-I53-50A + I53-50B.4PT1
**β-RBD-NP**	Figure 4, 5A, 5B	β-RBD-I53-50A + I53-50B.4PT1
**βRpk9-RBD-NP**	Figure 4, 5B, 5E, 5F	βRpk9-RBD-I53-50A + I53-50B.4PT1
**γ-RBD-NP**	Figure 4, 5A, 5B	γ-RBD-I53-50A + I53-50B.4PT1
**γRpk9-RBD-NP**	Figure 4, 5B, 5E, 5F	γRpk9-RBD-I53-50A + I53-50B.4PT1
**E484K-RBD-NP**	Figure 5A, 5B	E484K-RBD-I53-50A + I53-50B.4PT1
**E484KRpk9-RBD-NP**	Figure 5B	E484KRpk9-RBD-I53-50A + I53-50B.4PT1

**cWu-1β-RBD-NP**	Figure 4, 5A, 5B	**Wu-1-RBD-NP** (Wu-1-RBD-I53-50A + I53-50B.4PT1) + **β-RBD-NP** (β-RBD-I53-50A + I53-50B.4PT1)
**cWu-1-βRpk9-RBD-NP**	Figure 4, 5E, 5F	**Wu-1-RBD-NP** (Wu-1-RBD-I53-50A + I53-50B.4PT1) + **βRpk9-RBD-NP** (βRpk9-RBD-I53-50A + I53-50B.4PT1)
**cWu-1βRpk9-RBD-NP**	Figure 5B	**Wu-1Rpk9-RBD-NP** (Wu-1Rpk9-RBD-I53-50A + I53-50B.4PT1) + **βRpk9-RBD-NP** (βRpk9-RBD-I53-50A + I53-50B.4PT1)
**mWu-1β-RBD-NP**	Figure 5A, 5B	Wu-1-RBD-I53-50A + β-RBD-I53-50A + I53-50B.4PT1
**mWu-1βRpk9-RBD-NP**	Figure 5B	Wu-1Rpk9-RBD-I53-50A + βRpk9-RBD-I53-50A + I53-50B.4PT1
**cWu-1γ-RBD-NP**	Figure 4	**Wu-1-RBD-NP** (Wu-1-RBD-I53-50A + I53-50B.4PT1) + **γ-RBD-NP** (γ-RBD-I53-50A + I53-50B.4PT1)
**cWu-1-γRpk9-RBD-NP**	Figure 4, 5E, 5F	**Wu-1-RBD-NP** (Wu-1-RBD-I53-50A + I53-50B.4PT1) + **γRpk9-RBD-NP** (γRpk9-RBD-I53-50A + I53-50B.4PT1)
**cWu-1βγ-RBD-NP**	Figure 5A, 5B	**Wu-1-RBD-NP** (Wu-1-RBD-I53-50A + I53-50B.4PT1) **β-RBD-NP** (β-RBD-I53-50A + I53-50B.4PT1) + **γ-RBD-NP** (γ-RBD-I53-50A + I53-50B.4PT1)
**cWu-1βγRpk9-RBD-NP**	Figure 5B	**Wu-1Rpk9-RBD-NP** (Wu-1Rpk9-RBD-I53-50A + I53-50B.4PT1) + **βRpk9-RBD-NP** (βRpk9-RBD-I53-50A + I53-50B.4PT1) + **γRpk9-RBD-NP** (γRpk9-RBD-I53-50A + I53-50B.4PT1)
**mWu-1βγ-RBD-NP**	Figure 5A, 5B	Wu-1-RBD-I53-50A + β-RBD-I53-50A + γ-RBD-I53-50A + I53-50B.4PT1
**mWu-1βγRpk9-RBD-NP**	Figure 5B	Wu-1Rpk9-RBD-I53-50A + βRpk9-RBD-I53-50A + γRpk9-RBD-I53-50A + I53-50B.4PT1

Supplementary Figures

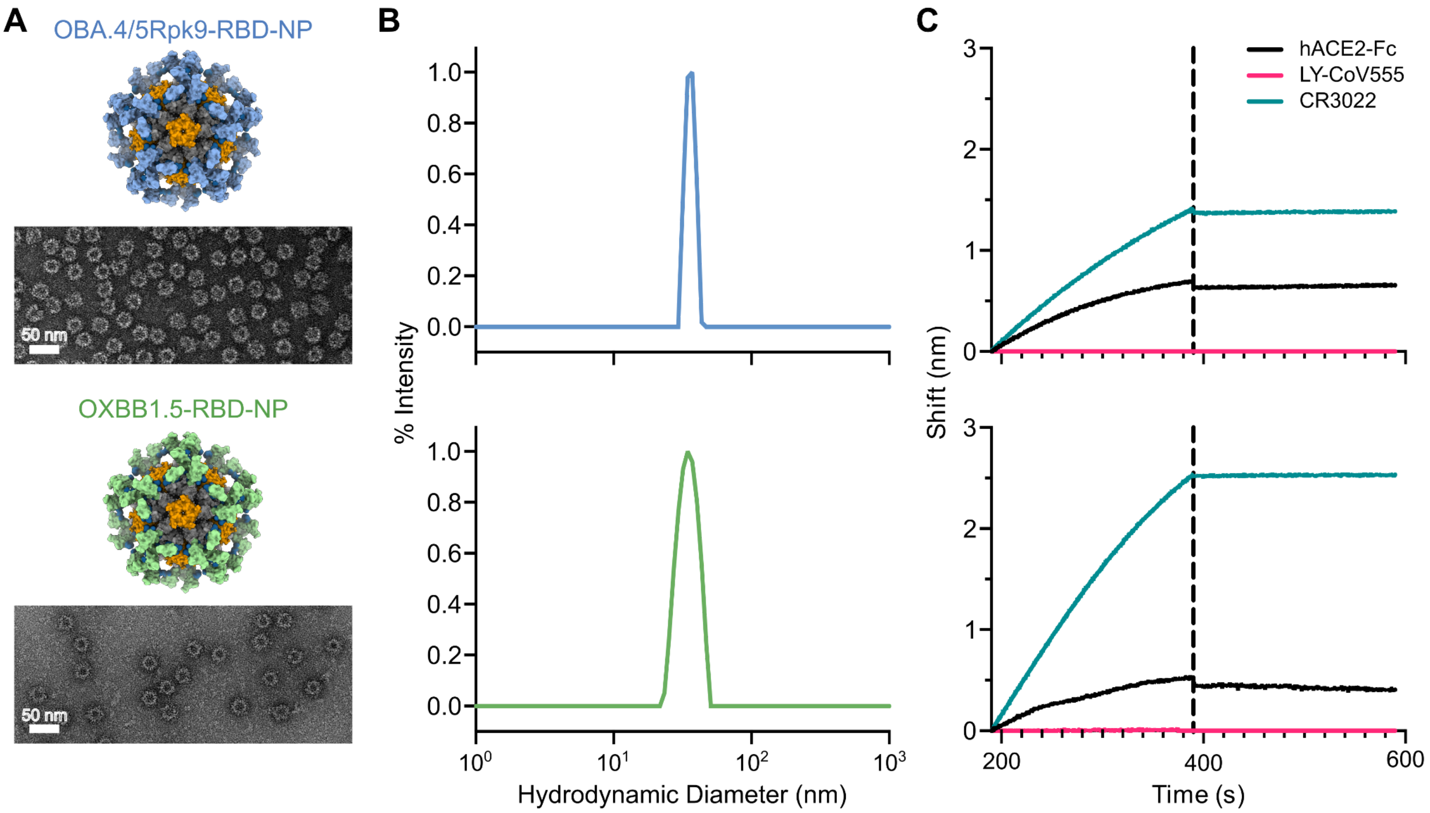

Supplementary Figure 1. SARS-CoV-2 Omicron variant vaccine design and characterization. A) Structural models of two monovalent RBD-NPs bearing the Omicron BA.4/5 RBD with Rpk9 mutations (OBA.4/5Rpk9) and the wild-type Omicron XBB.1.5 RBD (OXBB1.5) alongside representative nsEM micrographs of each immunogen. **B)** Representative DLS of OBA.4/5-9-RBD-NP (top) and OXBB1.5-RBD-NP (bottom). **C)** Representative BLI of OBA.4/5-9-RBD-NP (top) and OXBB1.5-RBD-NP (bottom) against hACE2-Fc, LY-CoV555, and CR3022 mAbs. All graphical representations of proteins made using ChimeraX[23].
