# Supplementary material for: Potent neutralization of SARS-CoV-2 variants by RBD nanoparticle and prefusion-stabilized spike immunogens
Source: NPJ Vaccines. 2024 Oct 8;9:184. doi: 10.1038/s41541-024-00982-1 (PMC11461925; doi:10.1038/s41541-024-00982-1)

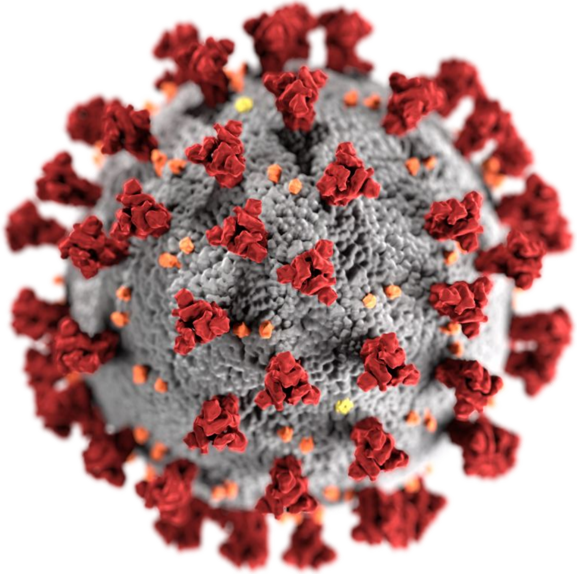

SARS-CoV-2 VOC-RBD-NP Stability Study

INSTITUTE FOR
Protein Design
UNIVERSITY of WASHINGTON

Table of Contents

- Stability Study Plan
- Wu-1-RBD-NP
- β-RBD-NP
- β9-RBD-NP
- γ-RBD-NP
- γ9-RBD-NP
- cWu-1β-RBD-NP
- cWu-1β9-RBD-NP
- cWu-1γ-RBD-NP
- cWu-1γ9-RBD-NP
- Biolayer Interferometry (BLI)

Stability Study Plan - 4 week duration at 4 temperatures

Buffer conditions - MAGiC Sauce: 50 mM Tris pH 7.4, 185 mM NaCl, 4.5% v/v glycerol, 0.75% w/v CHAPS, 100 mM Arginine

Reference: aliquots frozen at -80 C

Temperatures: -80 C, 2-8 C, 22-27 C, 37-40 C

Timepoints: T0, W1, W2, W3, W4

Timepoints analyzed in real time, on day aliquot removed from temperature

Aliquot stored in 0.5 mL screwcap tube with O ring

Wu-1-RBD-NP

nsEM for Wu-1-RBD-NP

Day 0

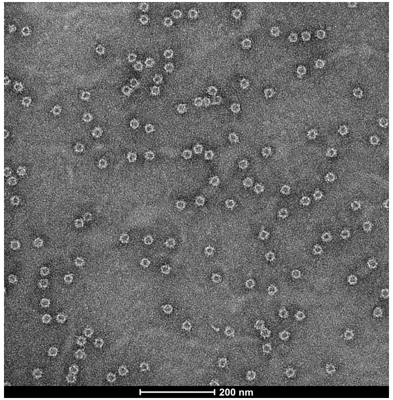

Day 28 -80 C

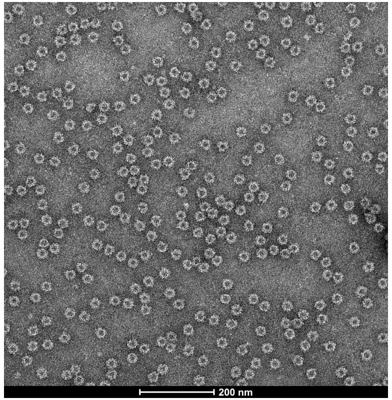

Day 28 2-8 C

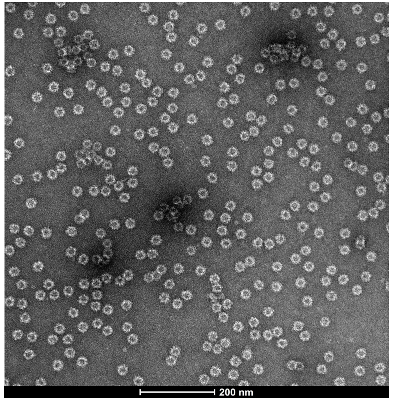

Day 28 22-25 C

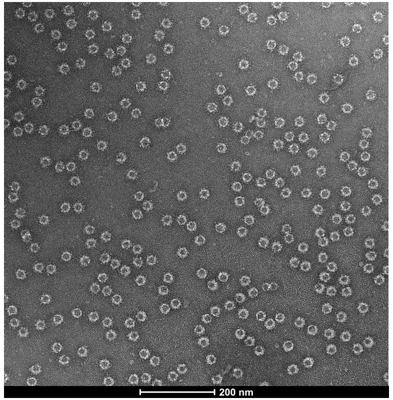

Day 28 35-40 C

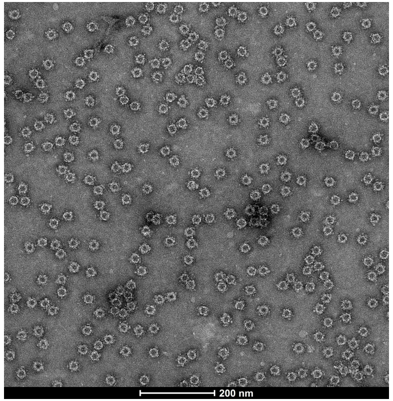

SDS-PAGE for Wu-1-RBD-NP

Day 0 | Day 7 | Day 14 | Day 21 | Day 28

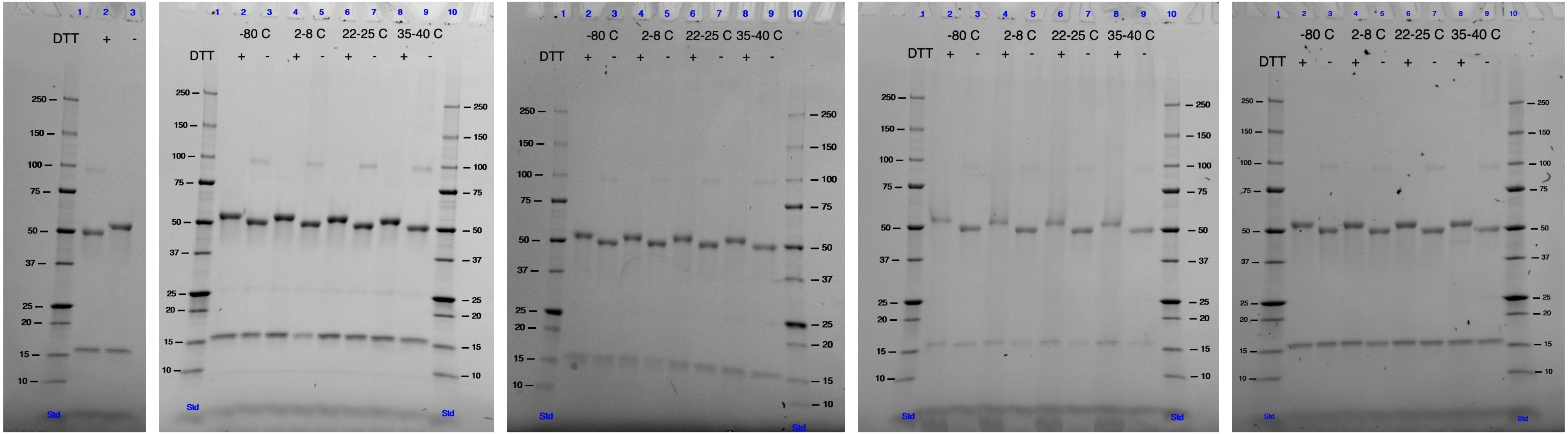

Dynamic Light Scattering for Wu-1-RBD-NP

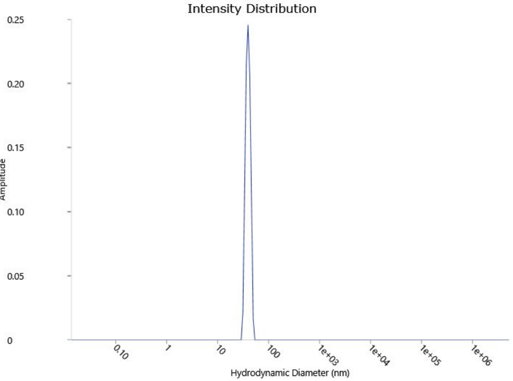

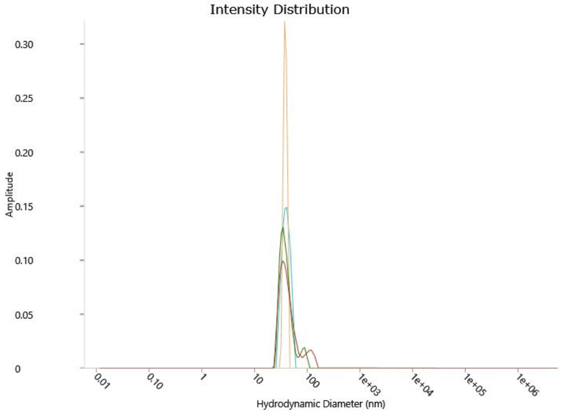

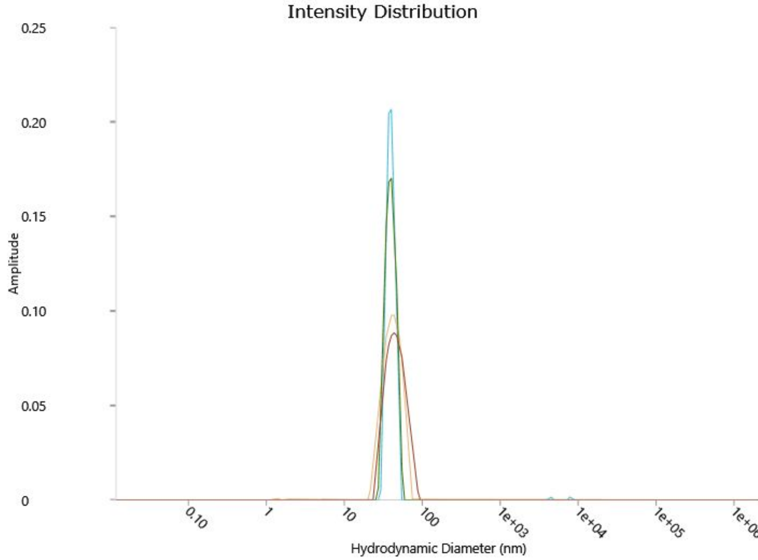

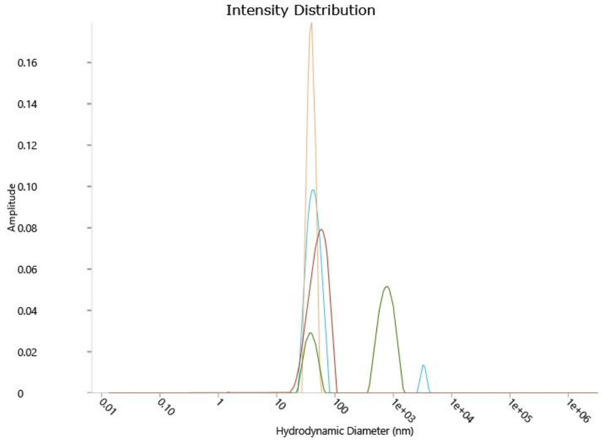

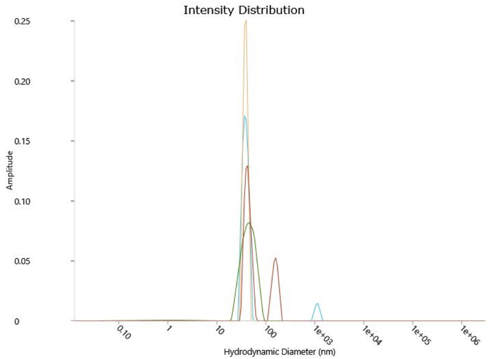

Absorbance at 320/280 for Wu-1-RBD-NP

Day 0

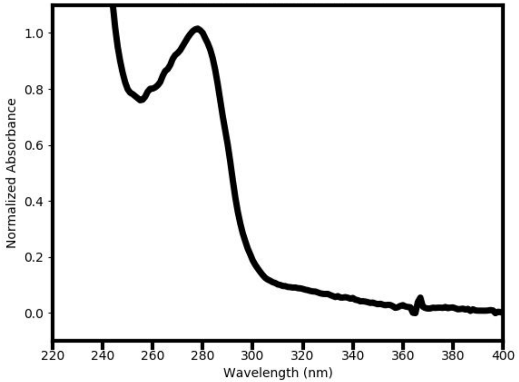

Day 7

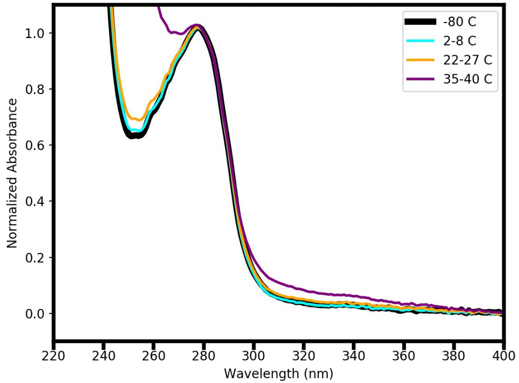

Day 14

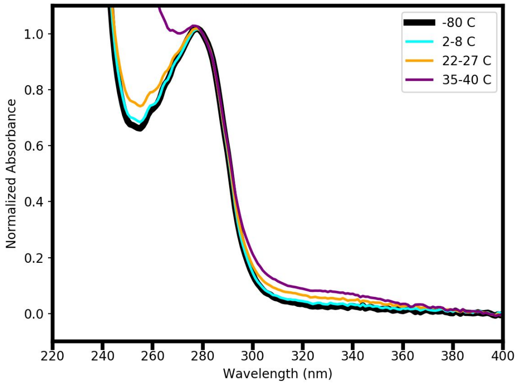

Day 21

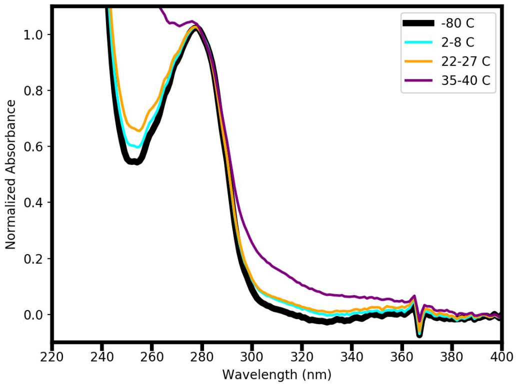

Day 28

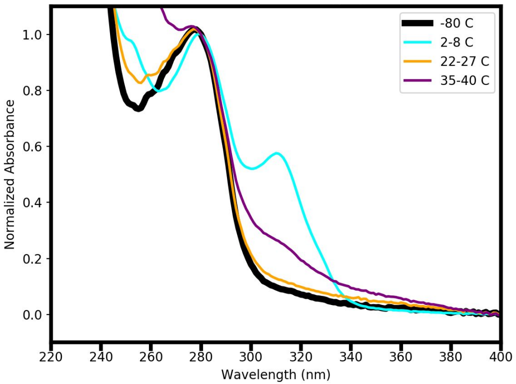

hACE2-Fc binding relative to <-70°C reference for Wu-1-RBD-NP

Day 0

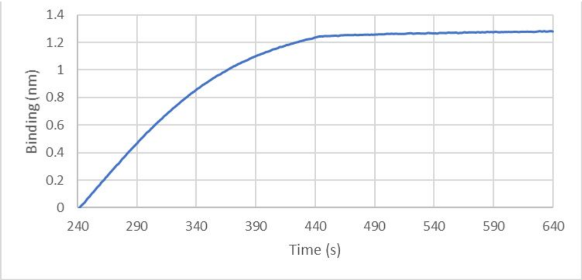

Day 7

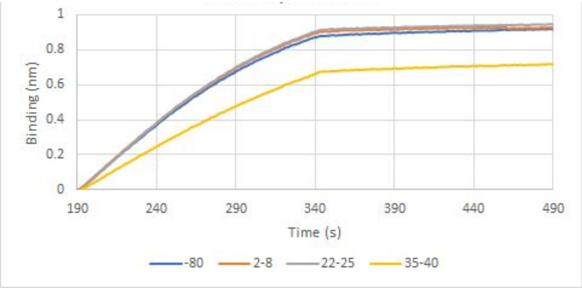

Day 14

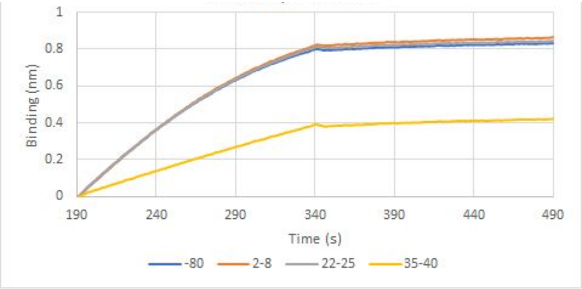

Day 21

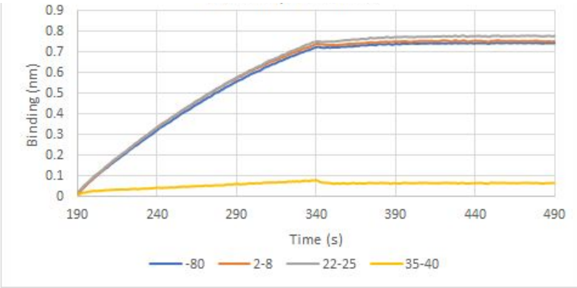

Day 28

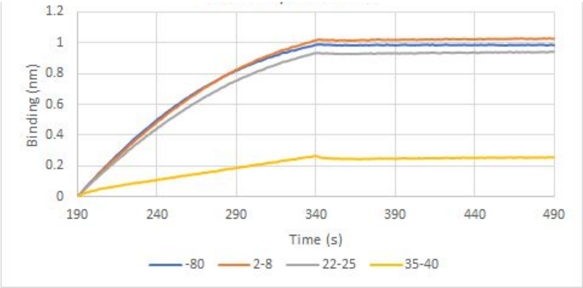

β-RBD-NP

nsEM for β-RBD-NP

Day 0

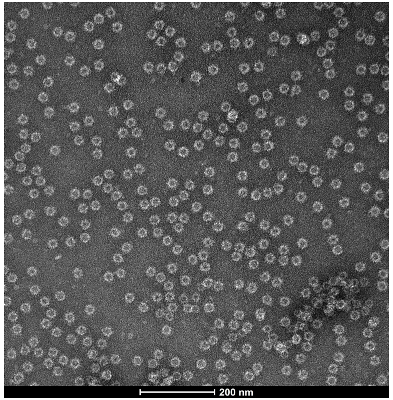

Day 28 -80 C

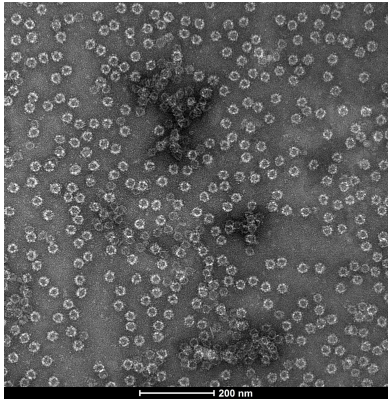

Day 28 2-8 C

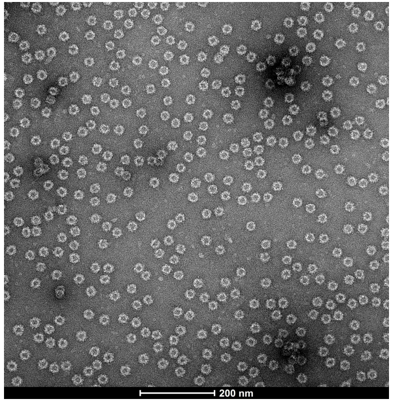

Day 28 22-25 C

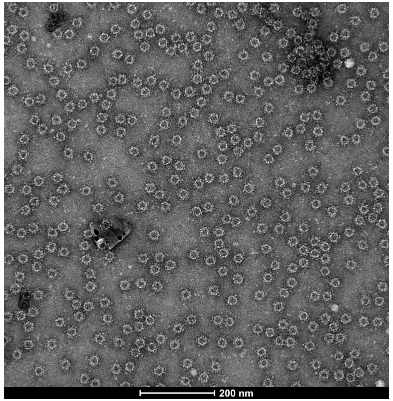

Day 28 35-40 C

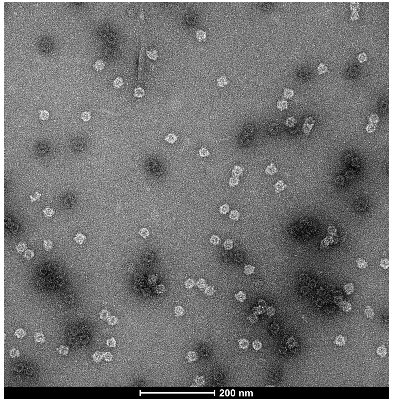

SDS-PAGE for β-RBD-NP

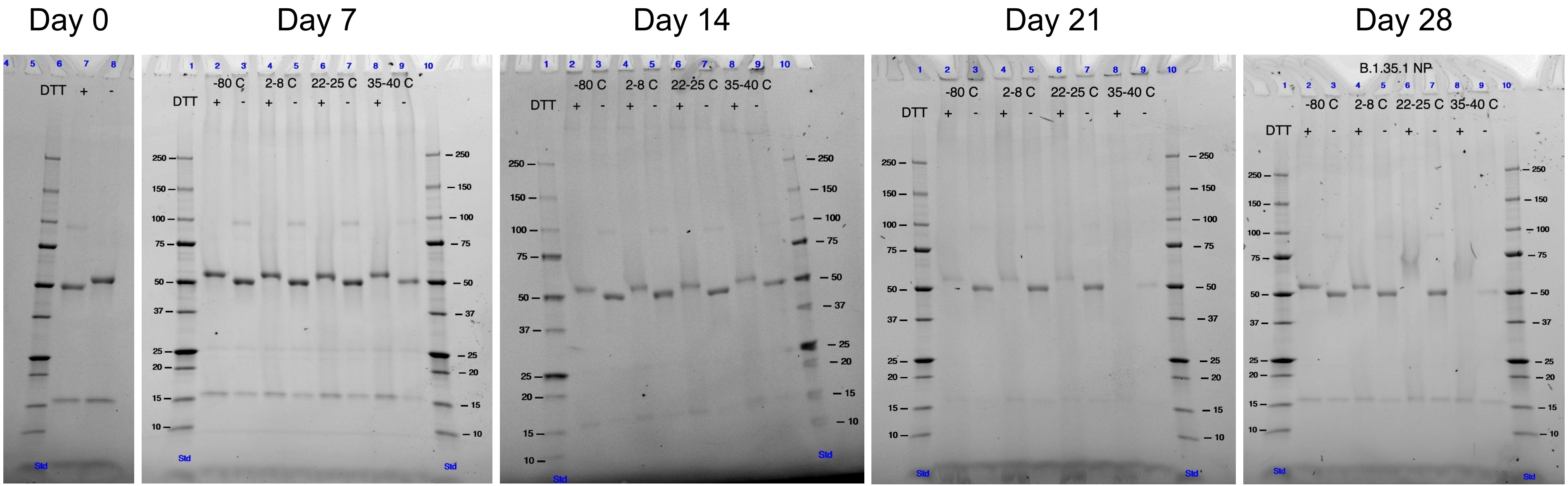

Dynamic Light Scattering for β-RBD-NP

Day 0

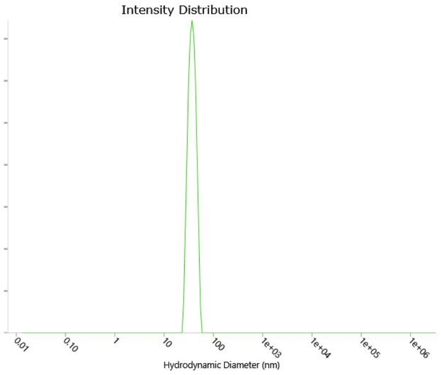

Day 7

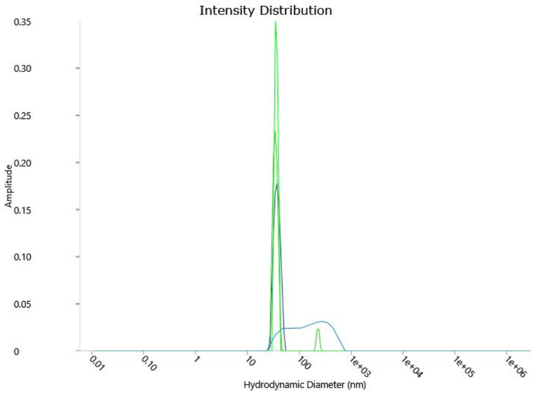

Day 14

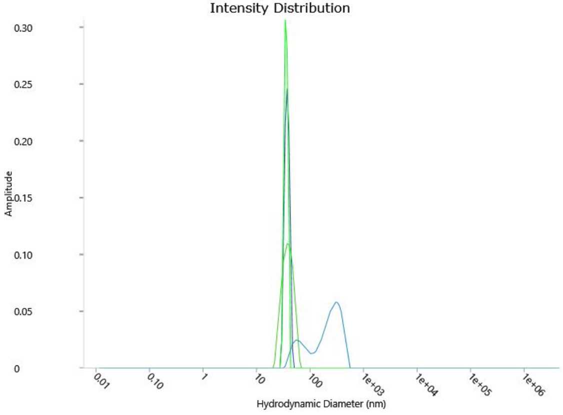

Day 21

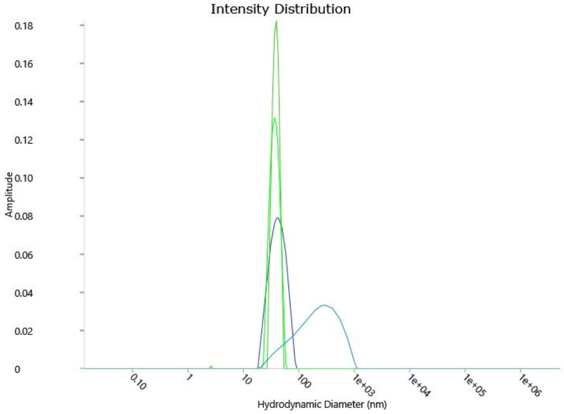

Day 28

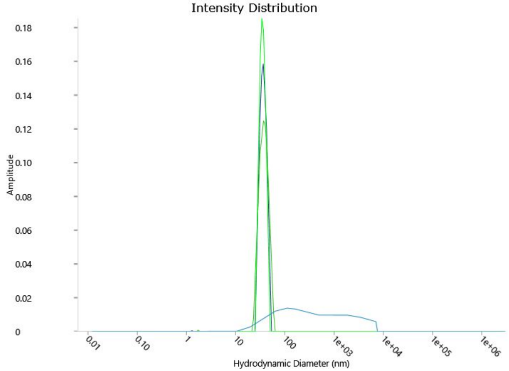

Absorbance at 320/280 for β-RBD-NP

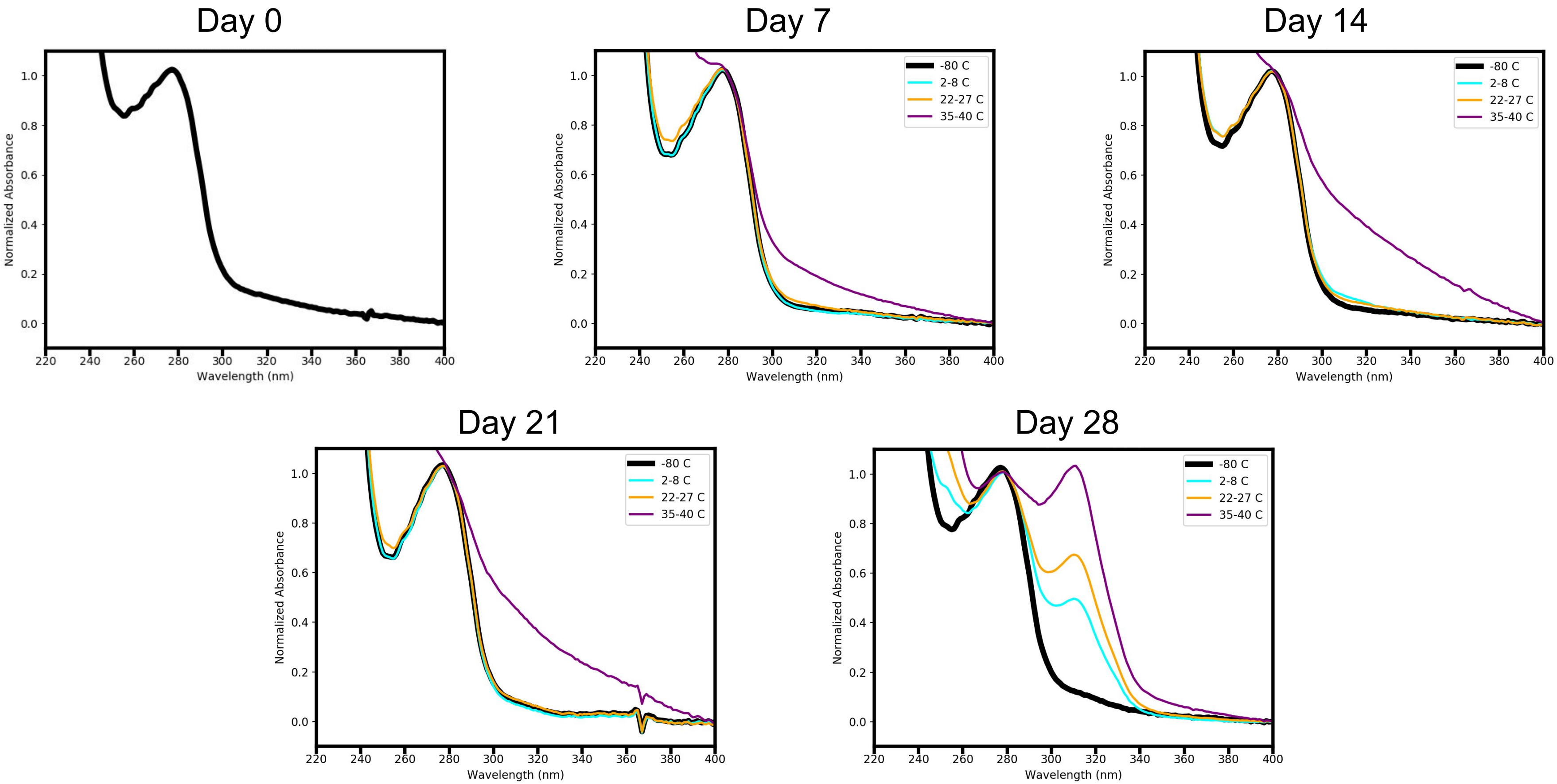

hACE2-Fc binding relative to <-70°C reference for β-RBD-NP

Day 0

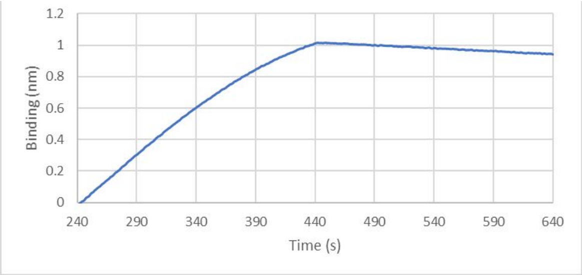

Day 7

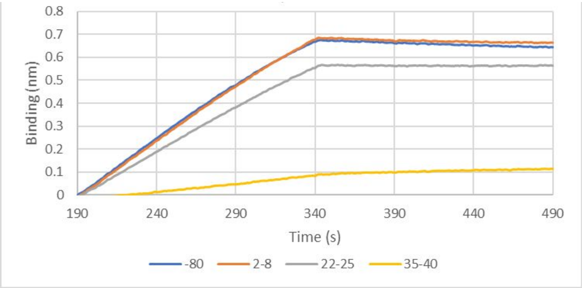

Day 14

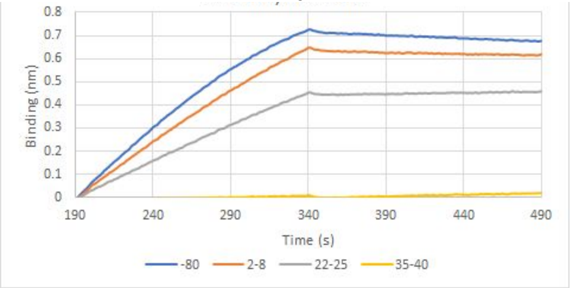

Day 21

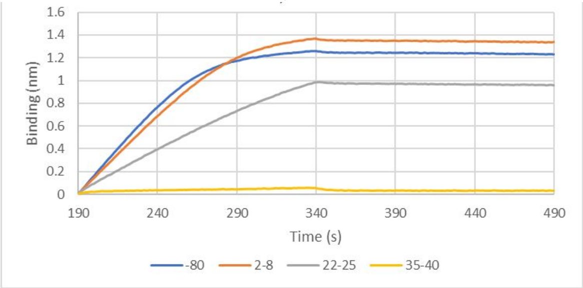

Day 28

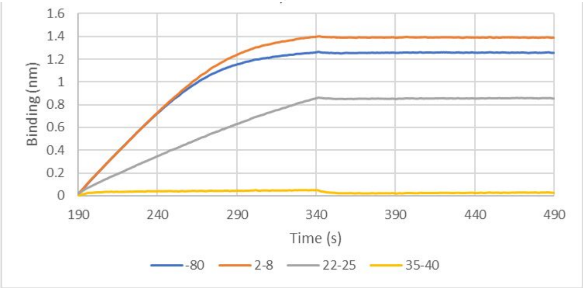

β9-RBD-NP

nsEM for β9-RBD-NP

Day 0

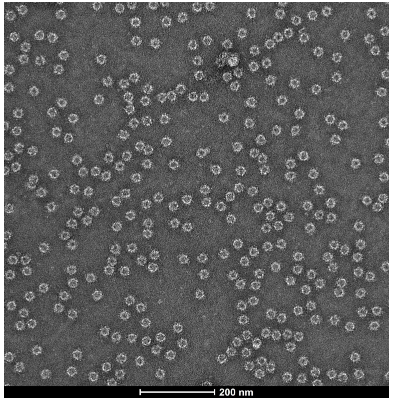

Day 28 -80 C

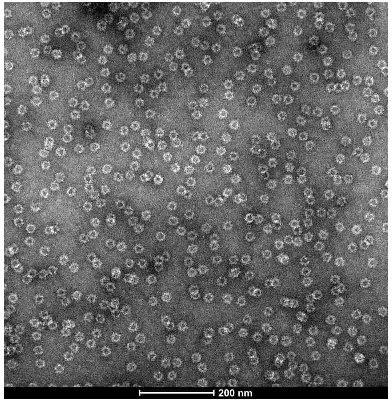

Day 28 2-8 C

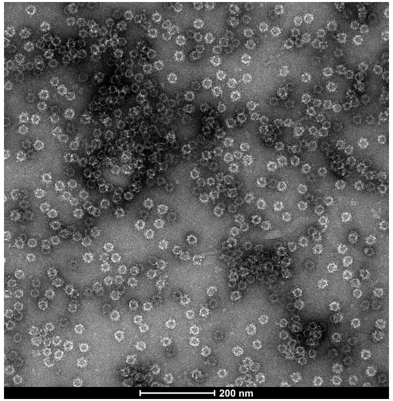

Day 28 22-25 C

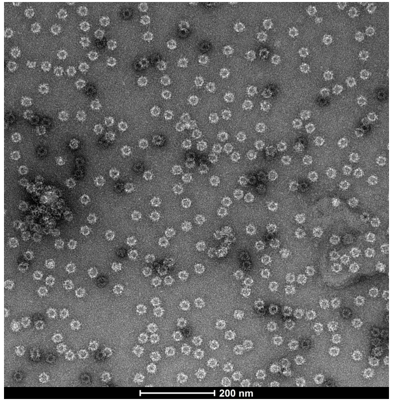

Day 28 35-40 C

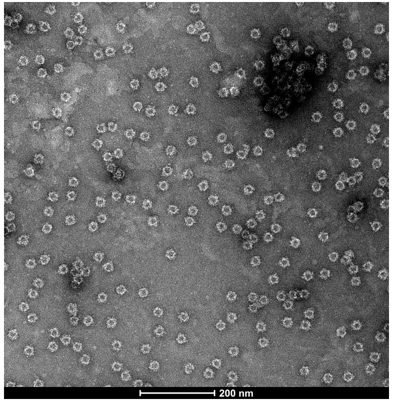

SDS-PAGE for β9-RBD-NP

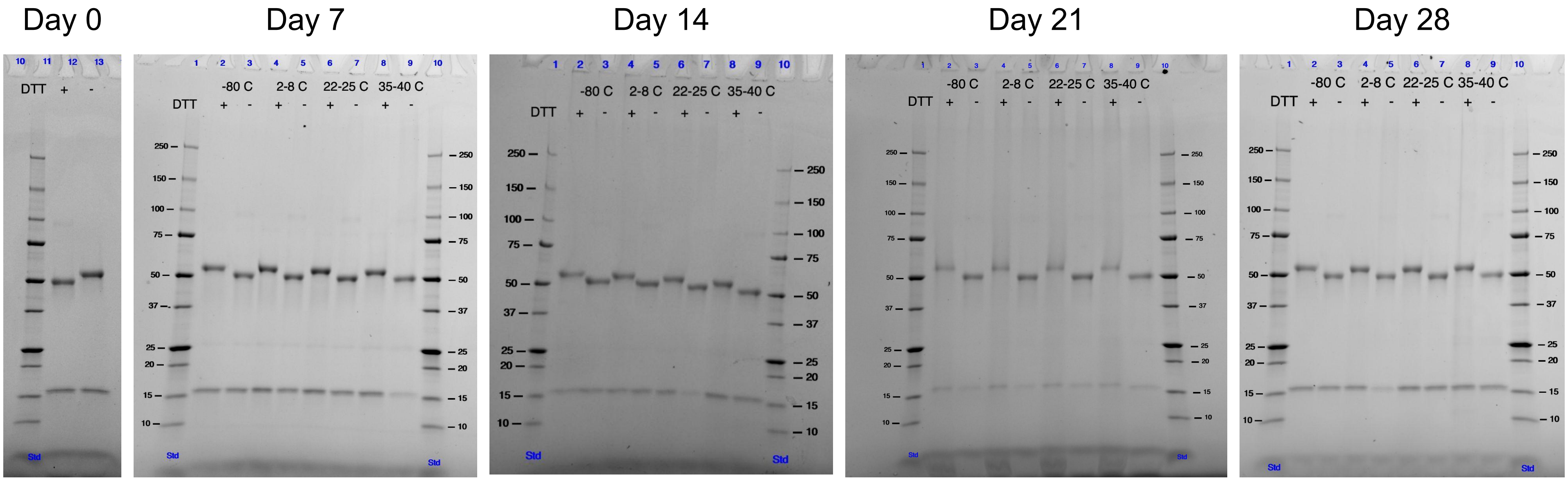

Dynamic Light Scattering for β9-RBD-NP

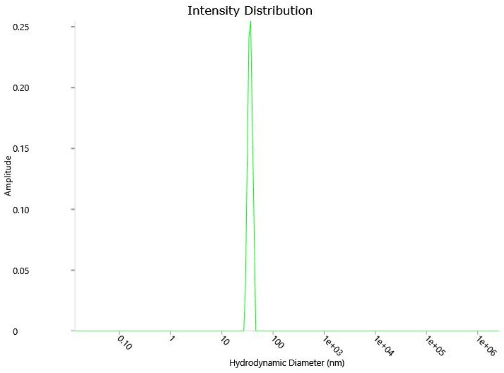

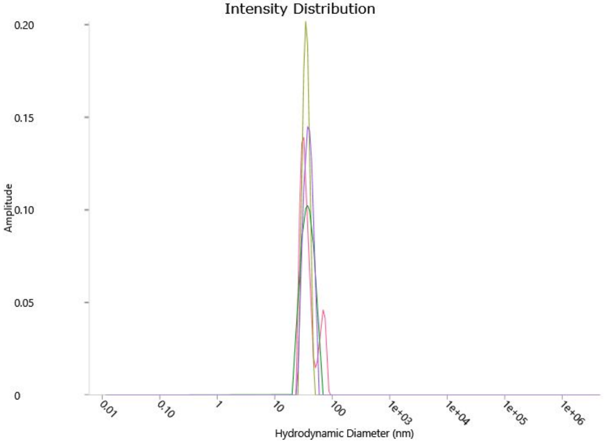

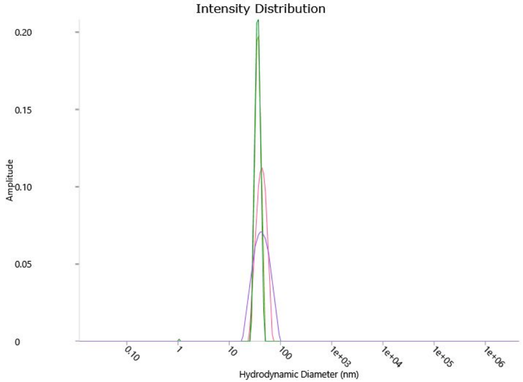

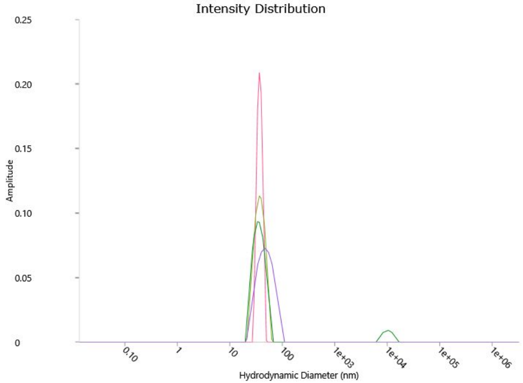

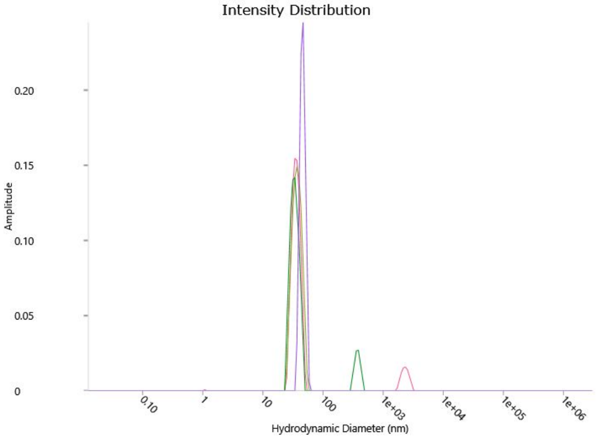

Absorbance at 320/280 for β9-RBD-NP

Day 0

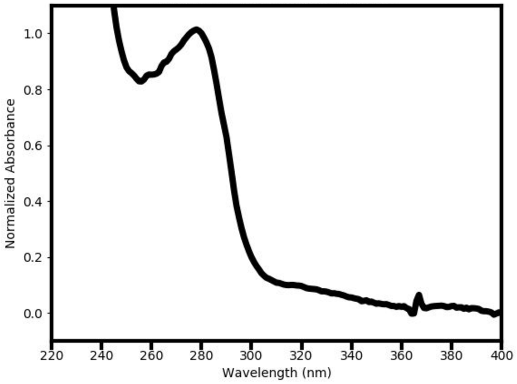

Day 7

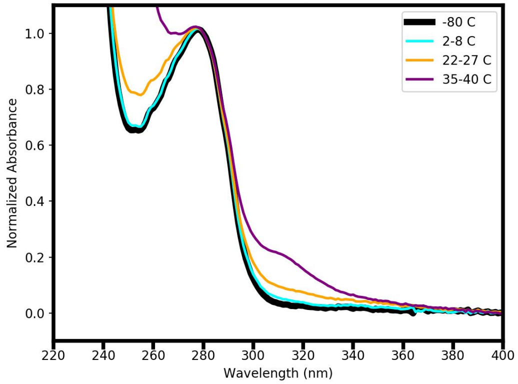

Day 14

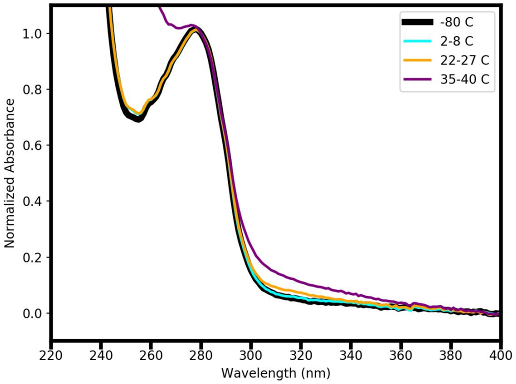

Day 21

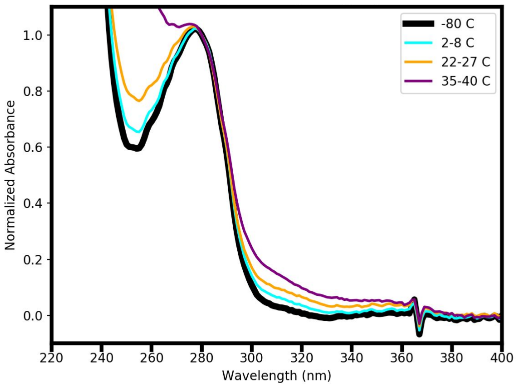

Day 28

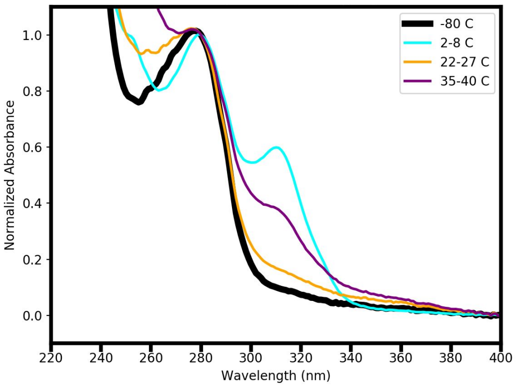

hACE2-Fc binding relative to <-70°C reference for β9-RBD-NP

Day 0

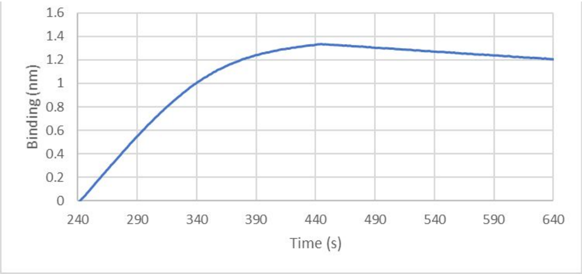

Day 7

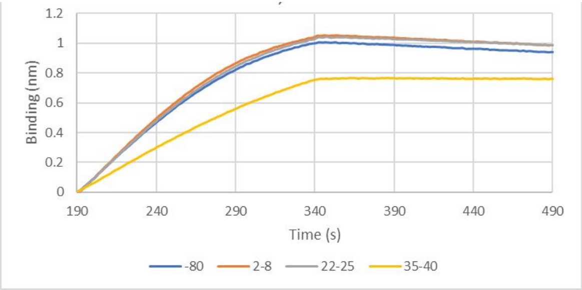

Day 14

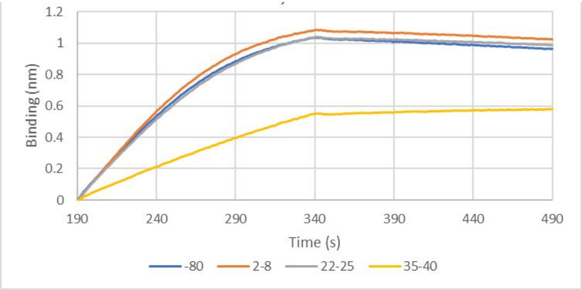

Day 21

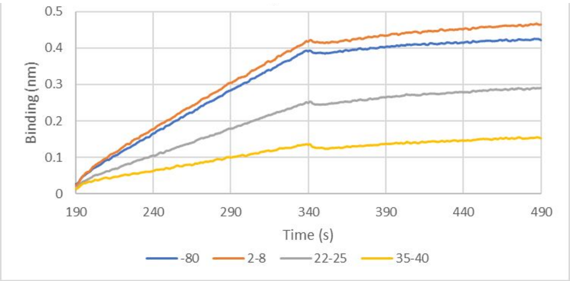

Day 28

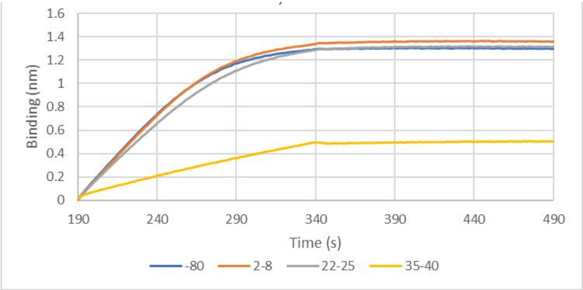

γ-RBD-NP

nsEM for γ-RBD-NP

Day 0

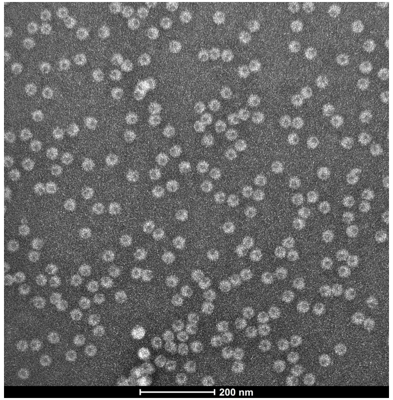

Day 28 -80 C

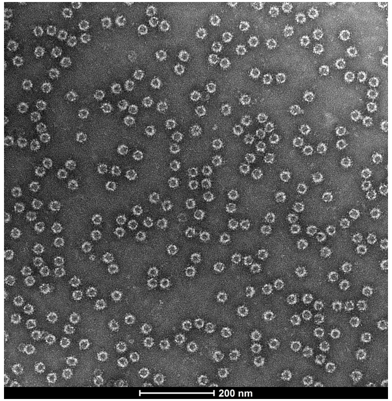

Day 28 2-8 C

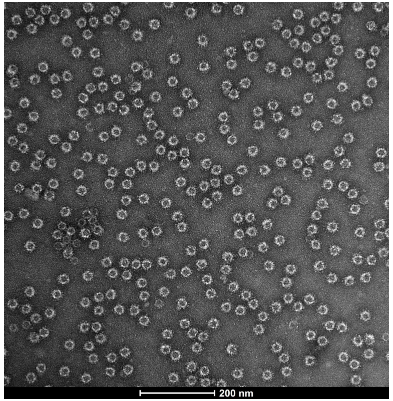

Day 28 22-25 C

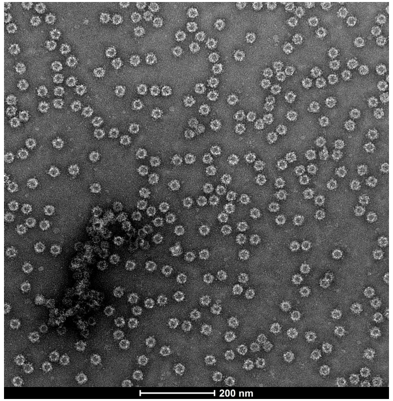

Day 28 35-40 C

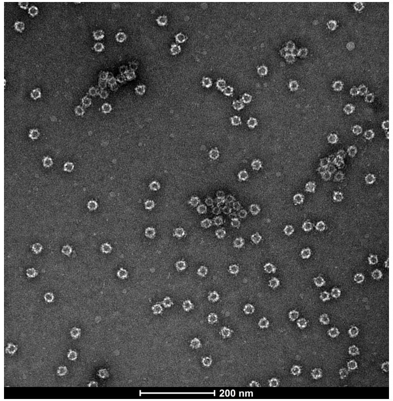

SDS-PAGE for γ-RBD-NP

Day 0 | Day 7 | Day 14 | Day 21 | Day 28

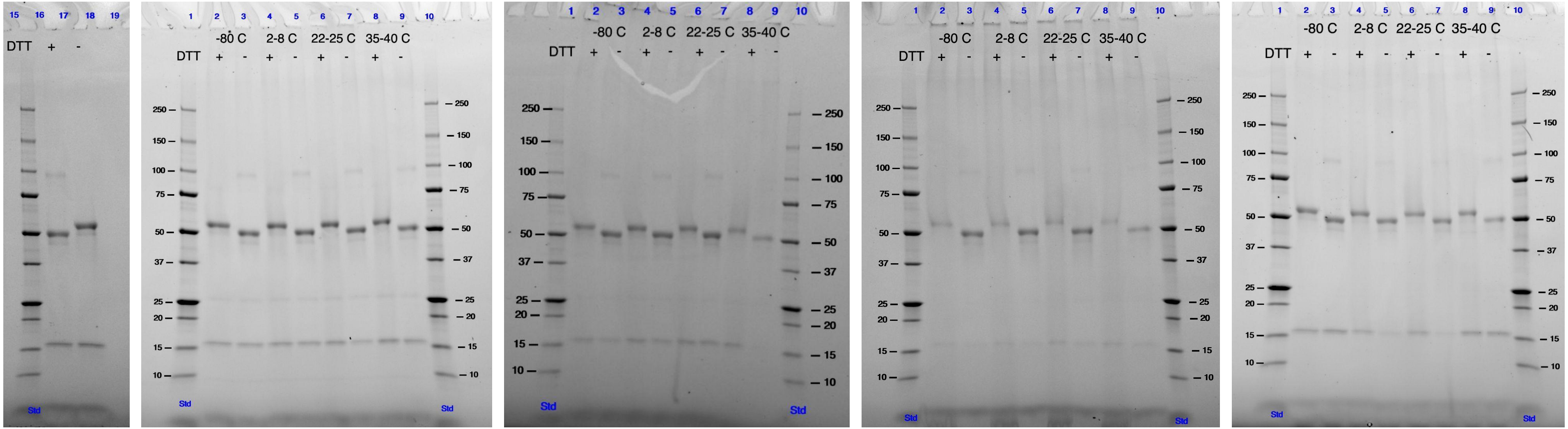

Dynamic Light Scattering for γ-RBD-NP

Day 0

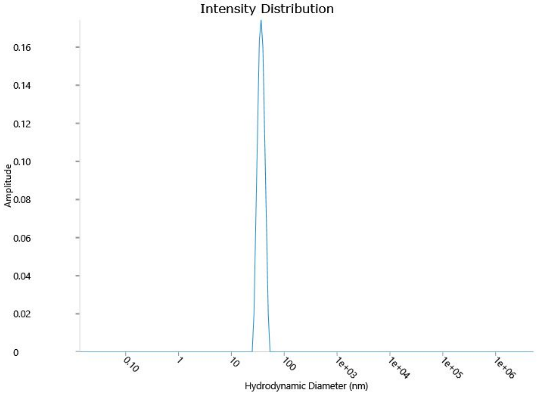

Day 7

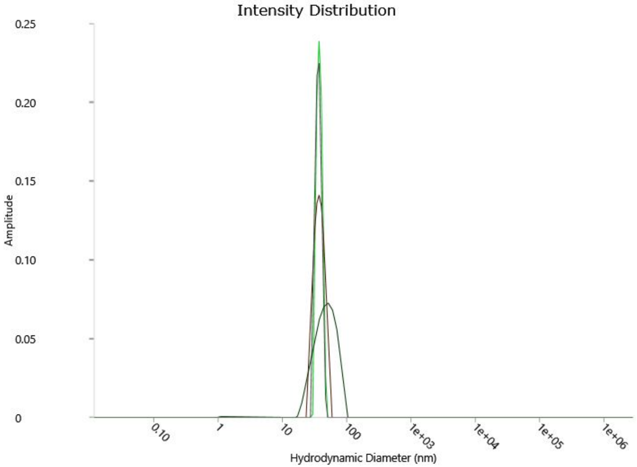

Day 14

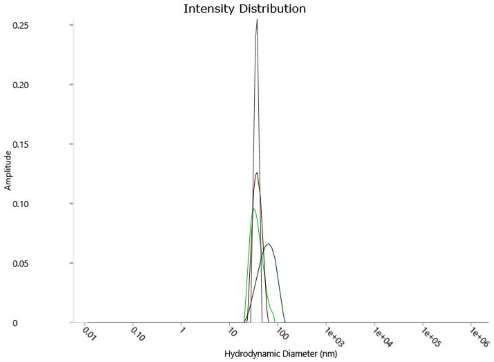

Day 21

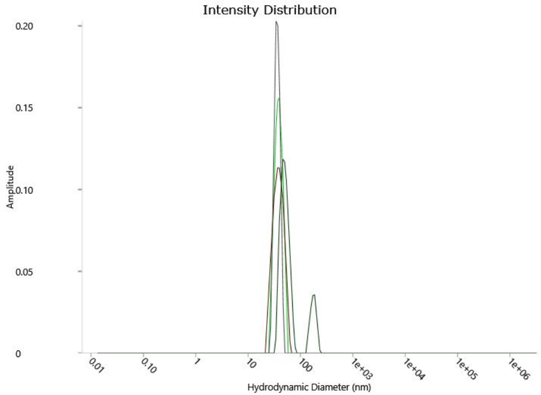

Day 28

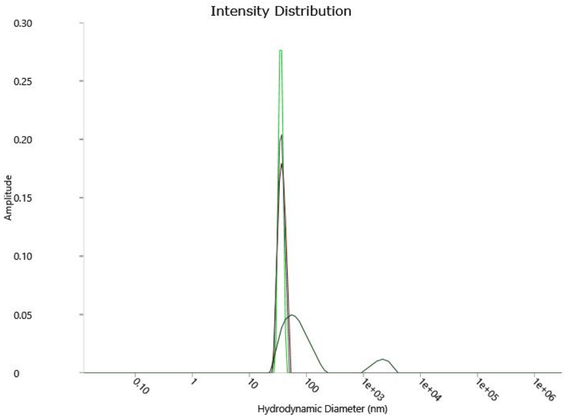

Absorbance at 320/280 for γ-RBD-NP

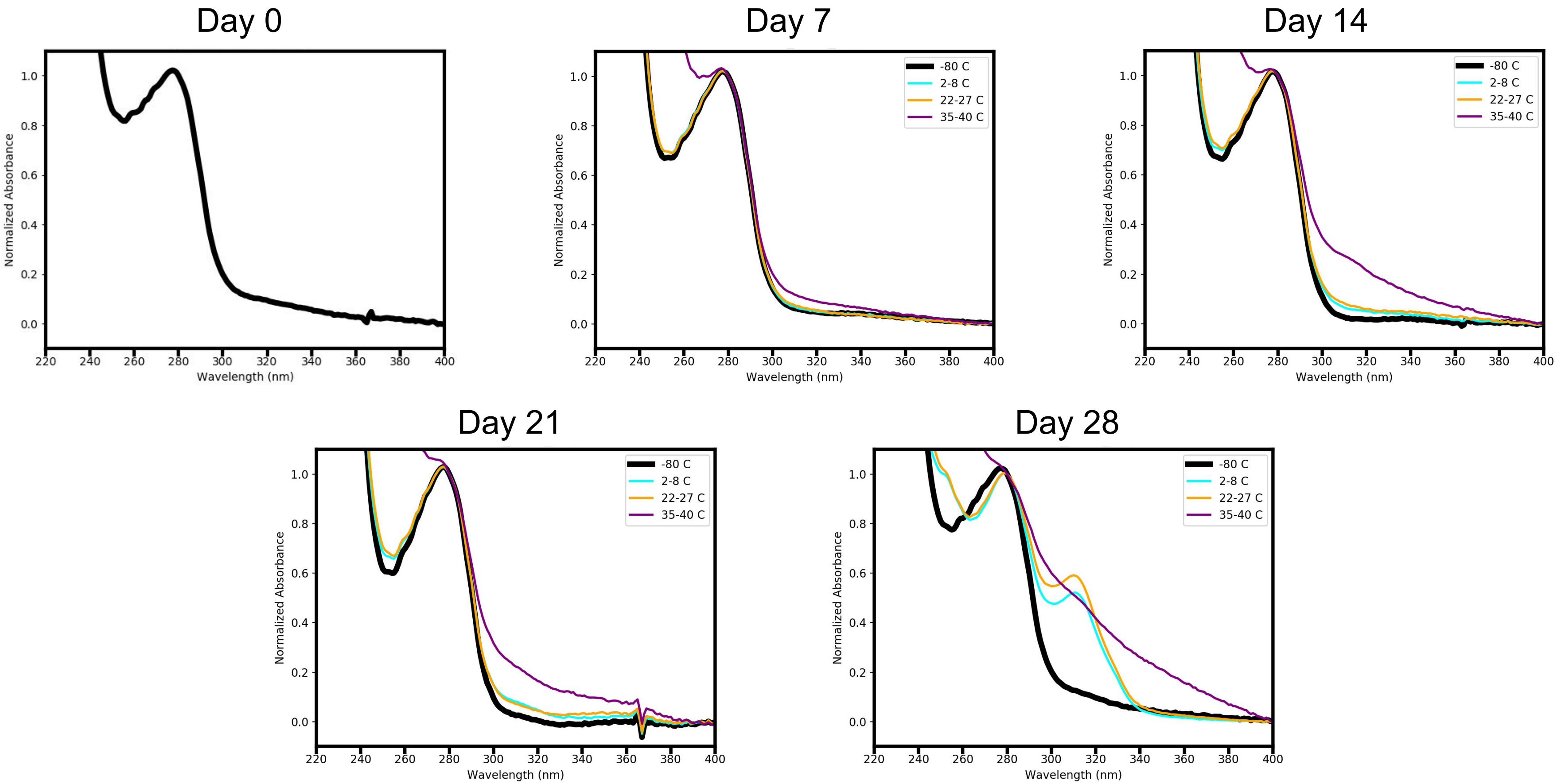

hACE2-Fc binding relative to <-70°C reference for γ-RBD-NP

Day 0

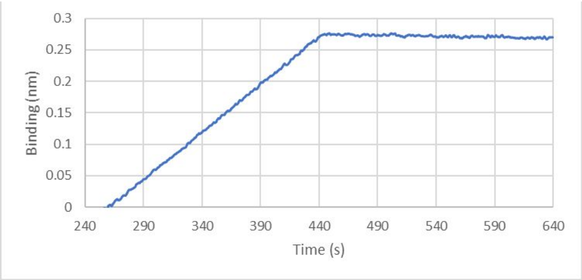

Day 7

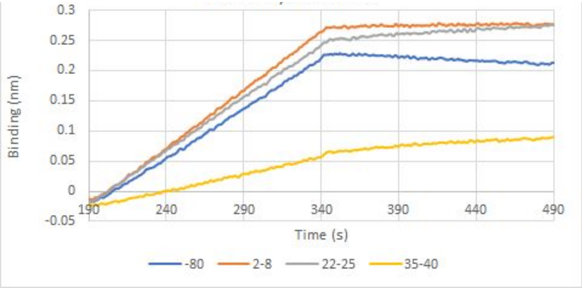

Day 14

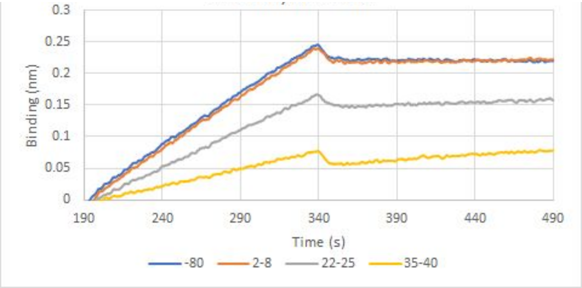

Day 21

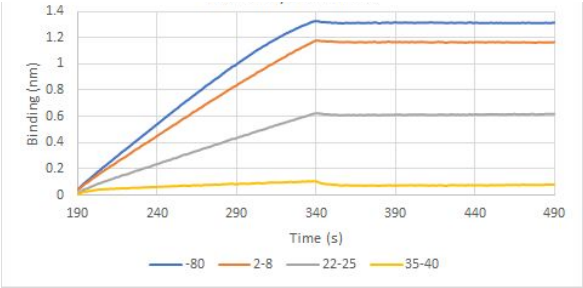

Day 28

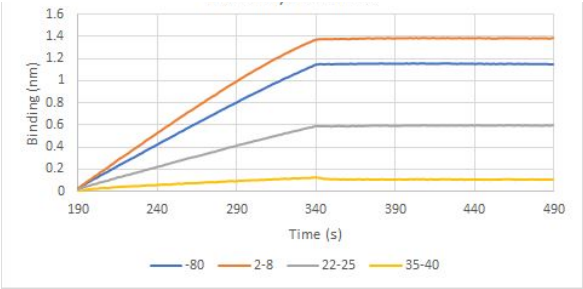

γ9-RBD-NP

nsEM for γ9-RBD-NP

Day 0

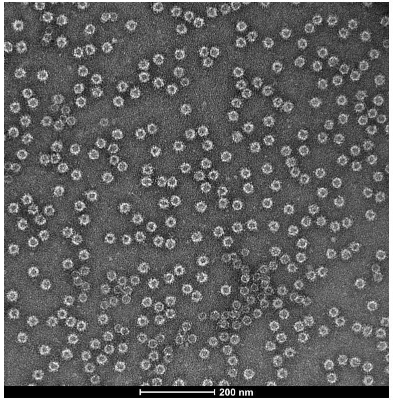

Day 28 -80 C

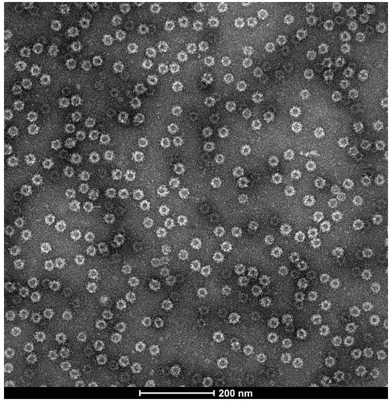

Day 28 2-8 C

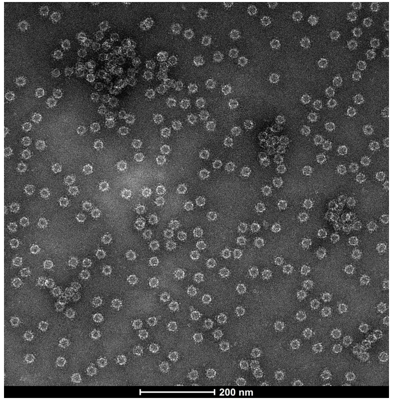

Day 28 22-25 C

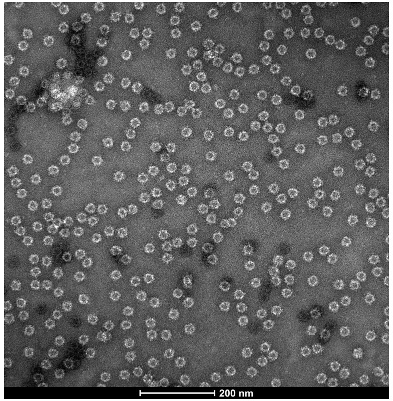

Day 28 35-40 C

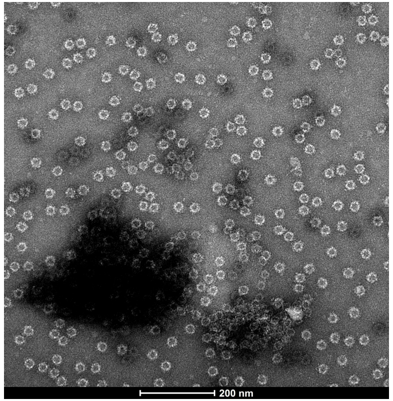

SDS-PAGE for γ9-RBD-NP

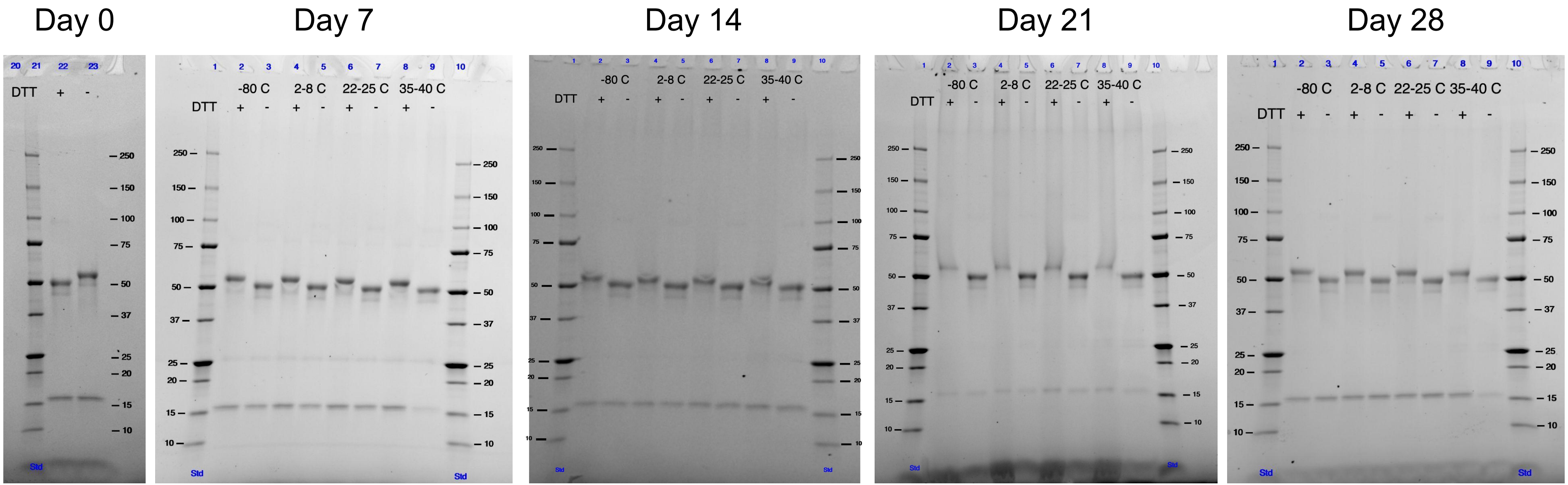

Dynamic Light Scattering for γ9-RBD-NP

Day 0

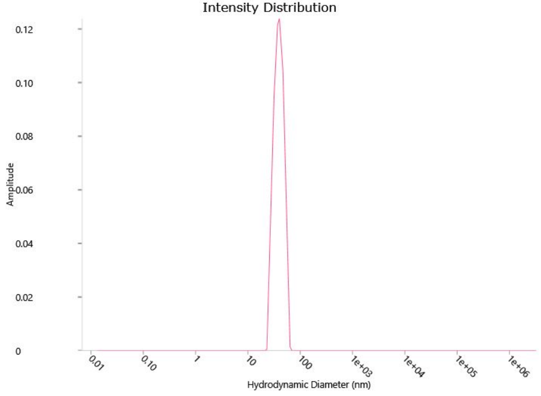

Day 7

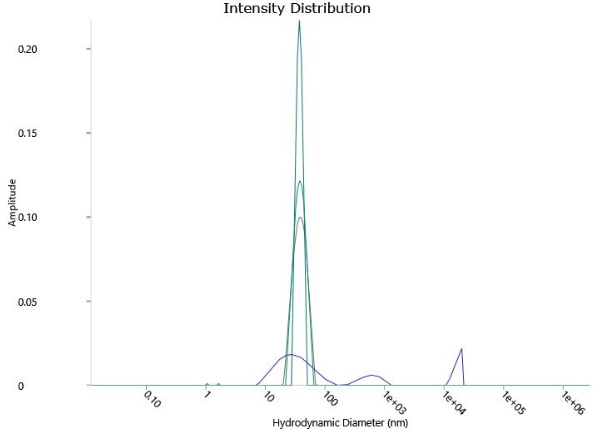

Day 14

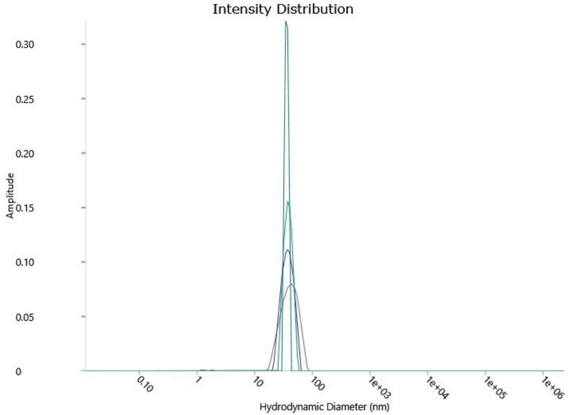

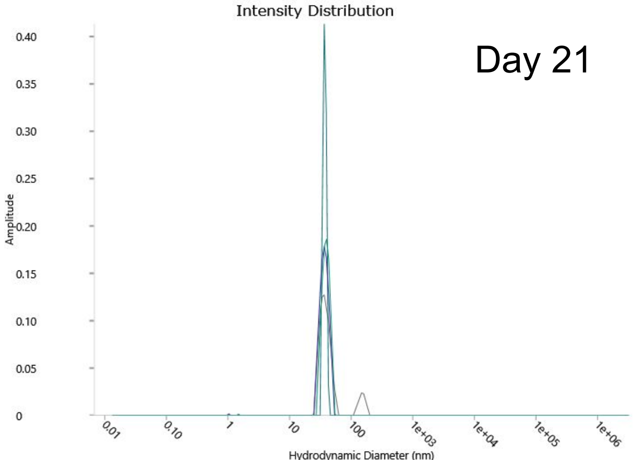

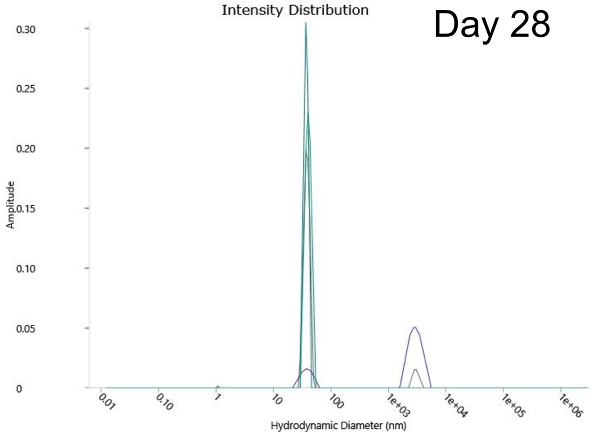

Absorbance at 320/280 for γ9-RBD-NP

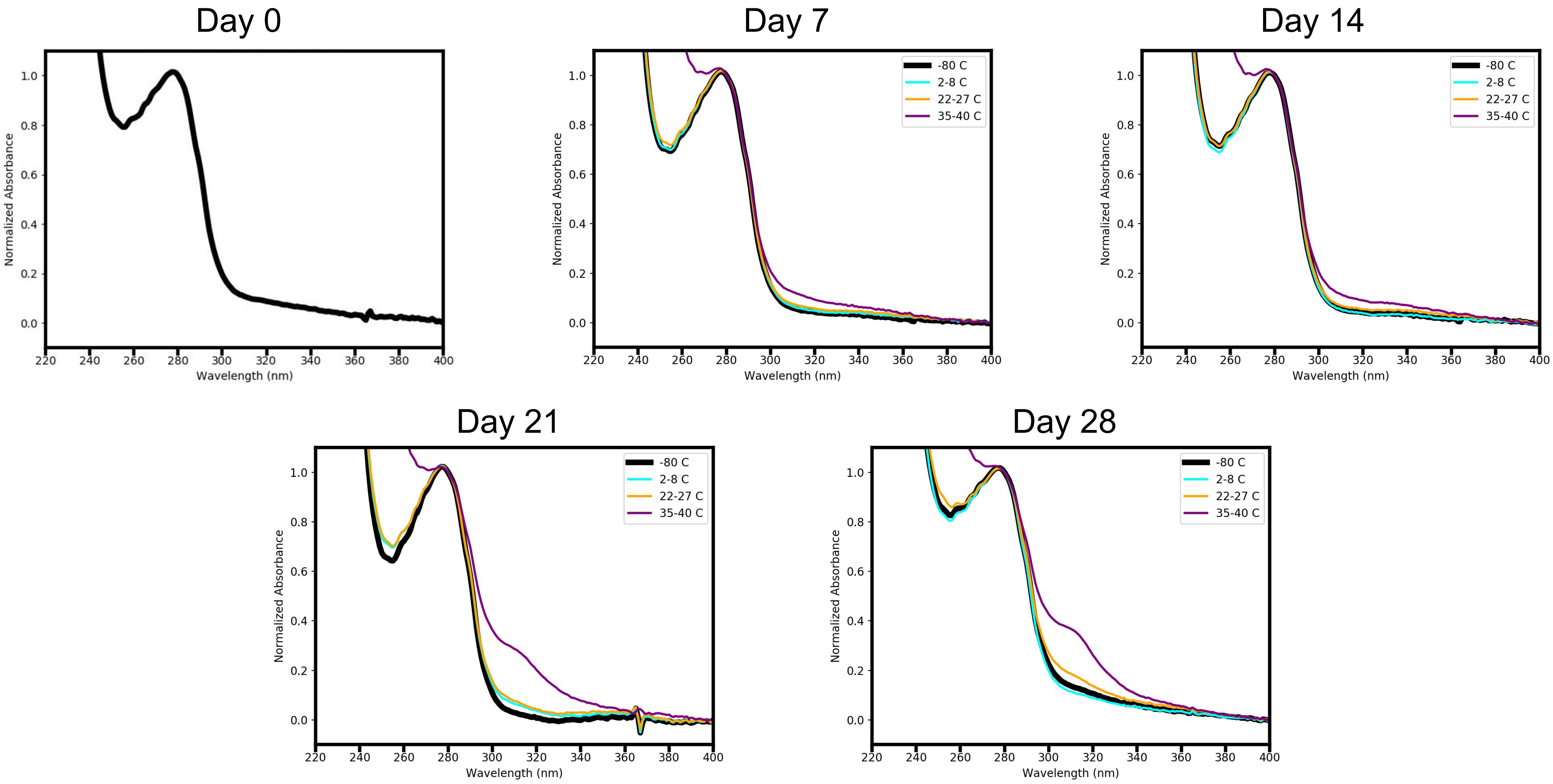

ACE2-Fc binding relative to <-70°C reference for γ9-RBD-NP

Day 0

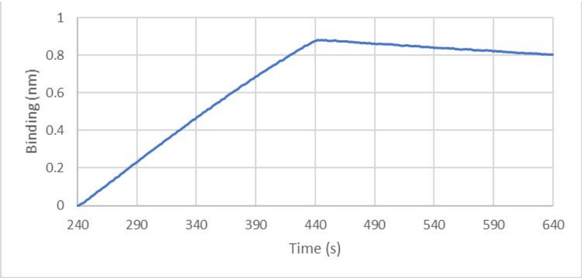

Day 7

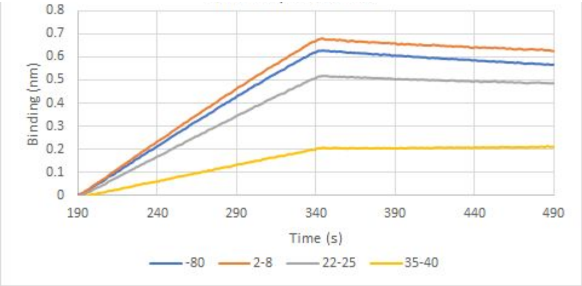

Day 14

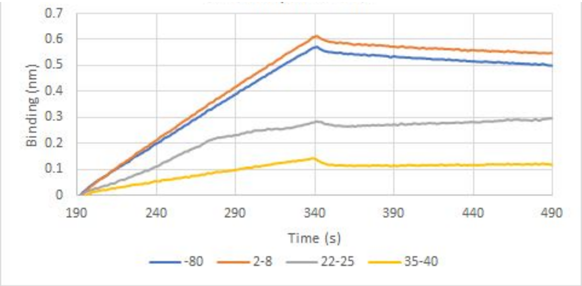

Day 21

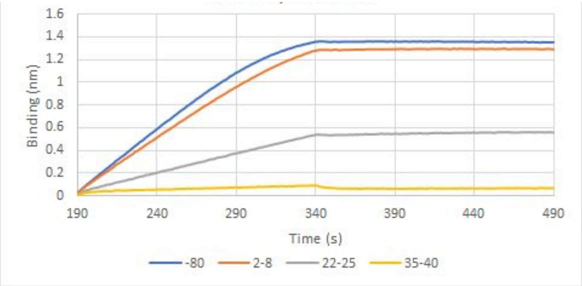

Day 28

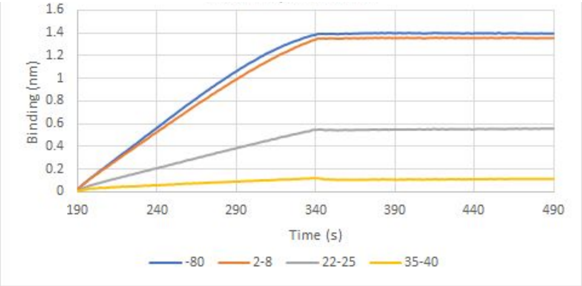

cWu-1β-RBD-NP

nsEM for cWu-1β-RBD-NP

Day 0

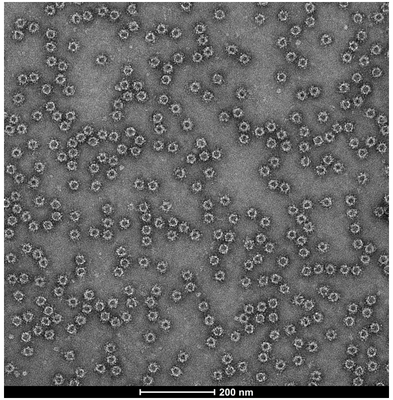

Day 28 -80 C

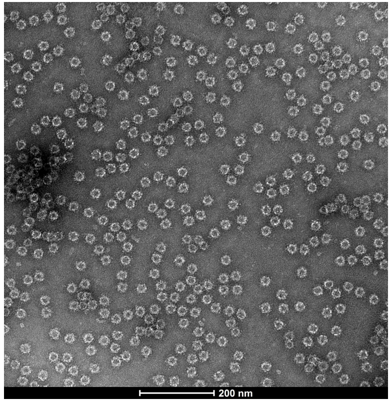

Day 28 2-8 C

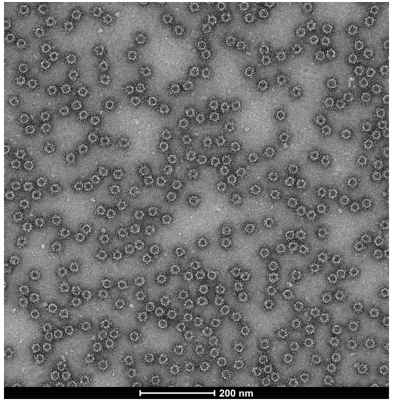

Day 28 22-25 C

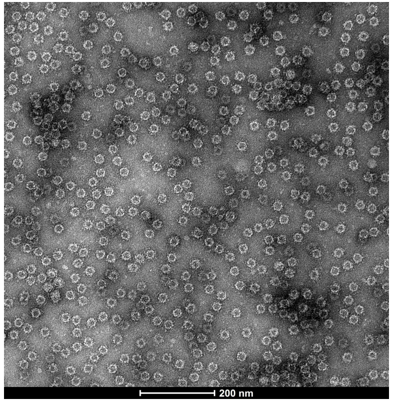

Day 28 35-40 C

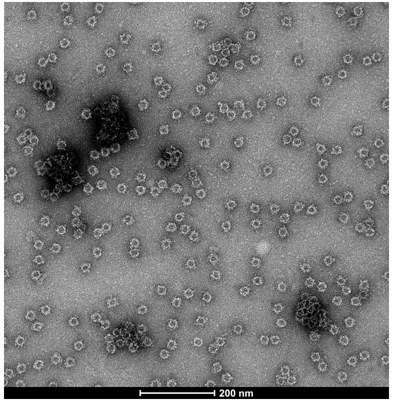

SDS-PAGE for cWu-1β-RBD-NP

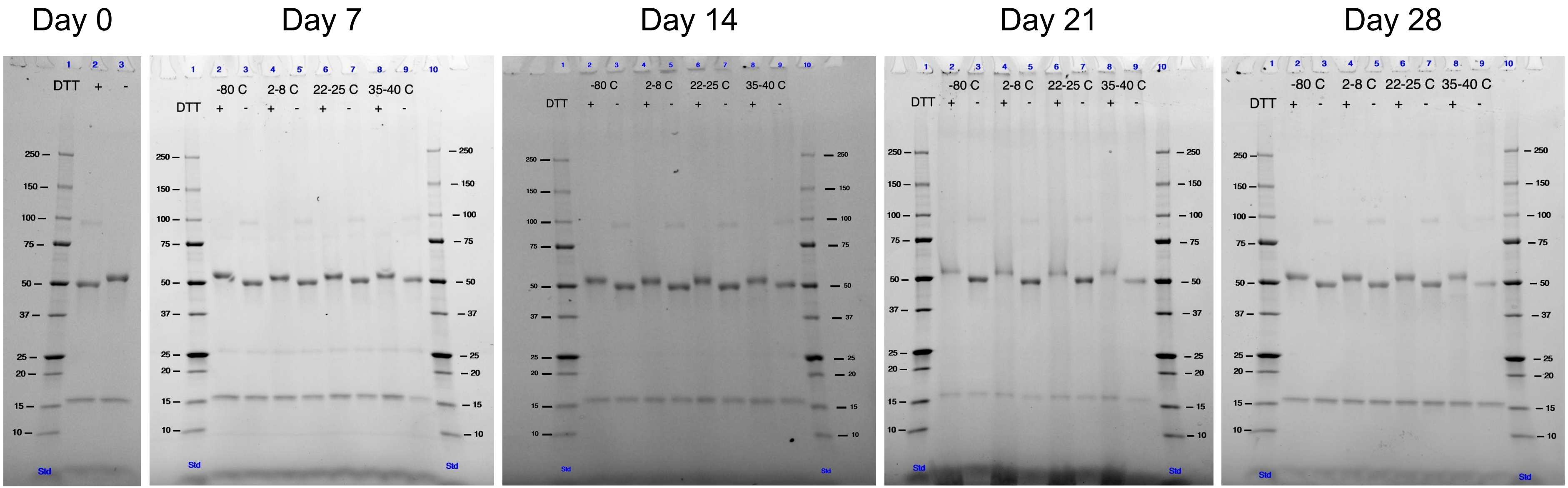

Dynamic Light Scattering for cWu-1β-RBD-NP

Day 0

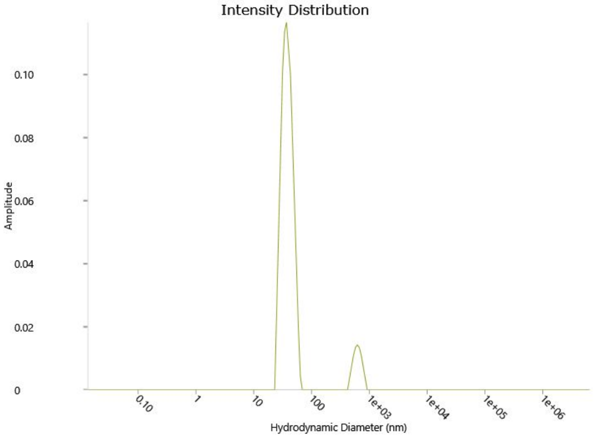

Day 7

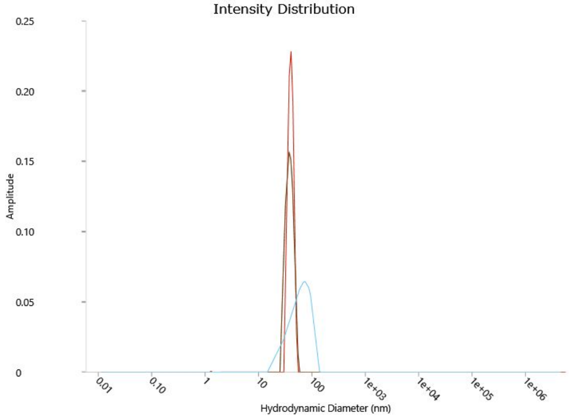

Day 14

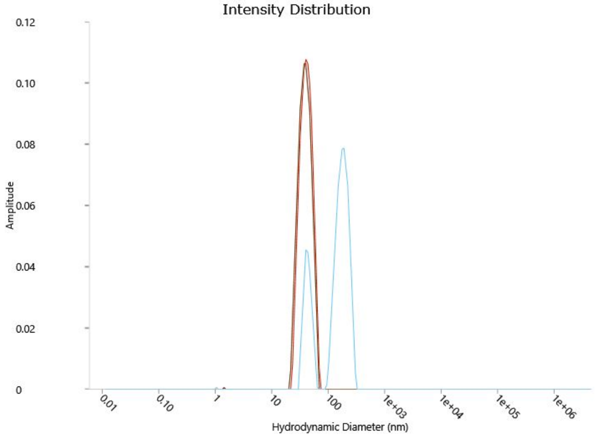

Day 21

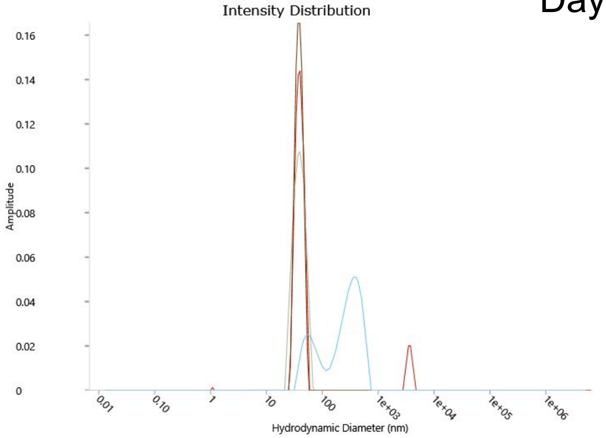

Day 28

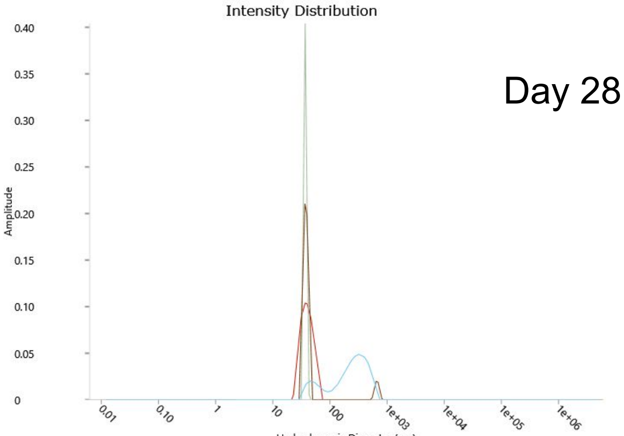

Absorbance at 320/280 for cWu-1β-RBD-NP

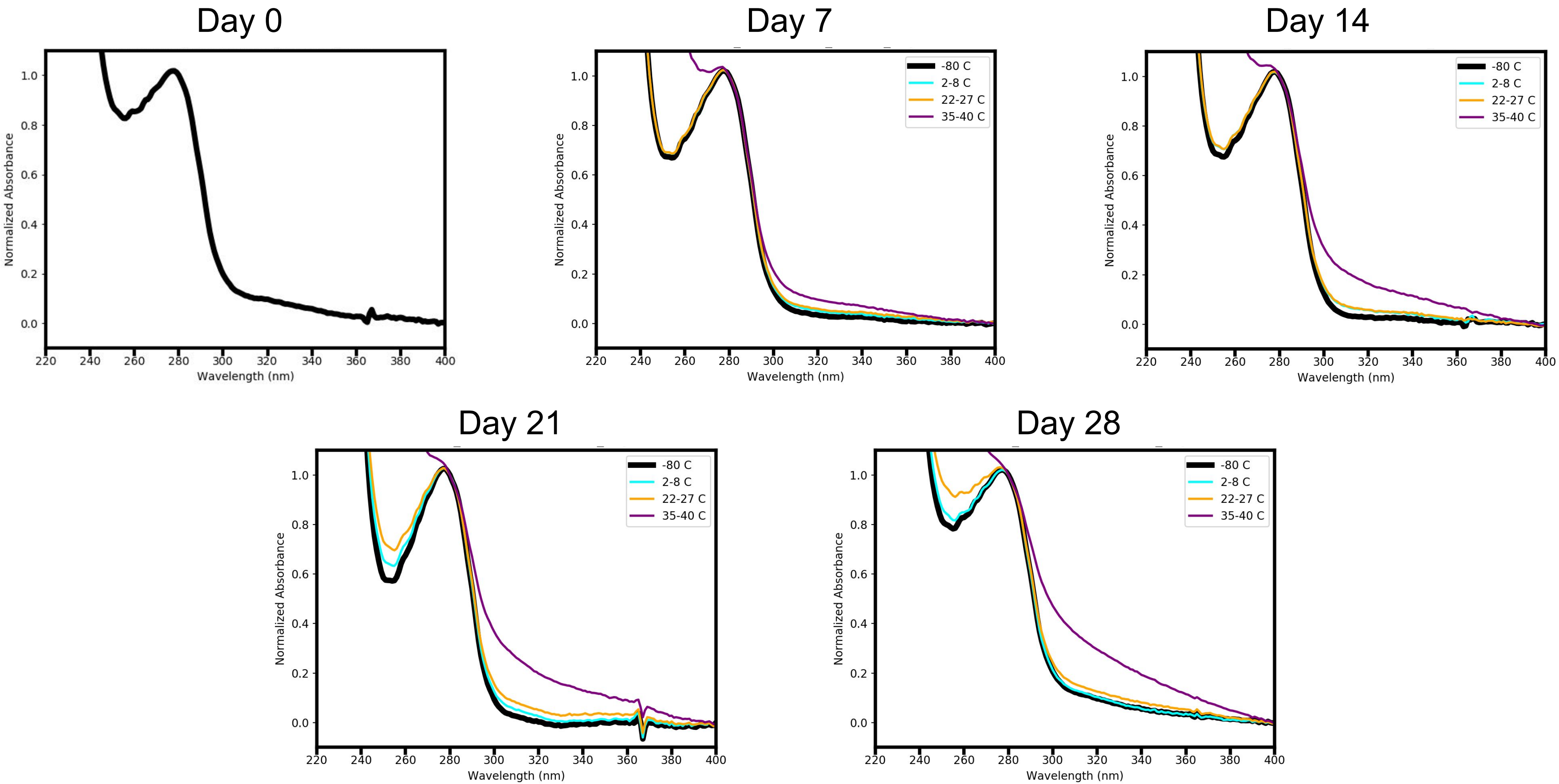

ACE2-Fc Binding Relative to <-70°C Reference for cWu-1β-RBD-NP

Day 0

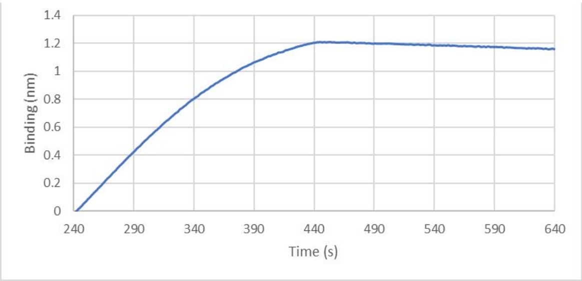

Day 7

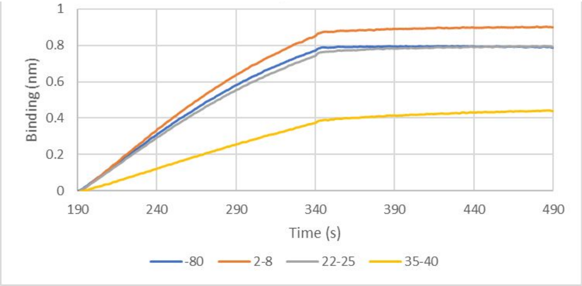

Day 14

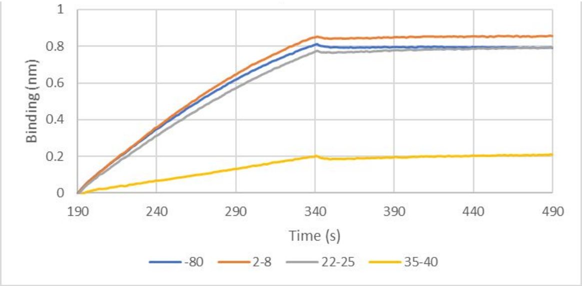

Day 21

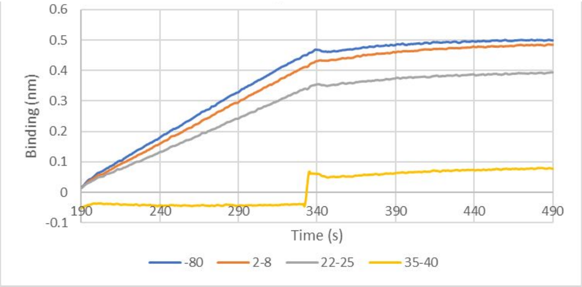

Day 28

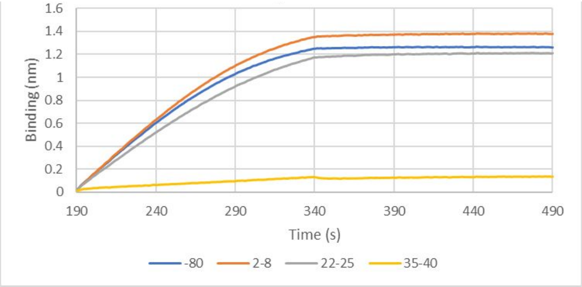

cWu-1β9-RBD-NP

nsEM for cWu-1β9-RBD-NP

Day 0

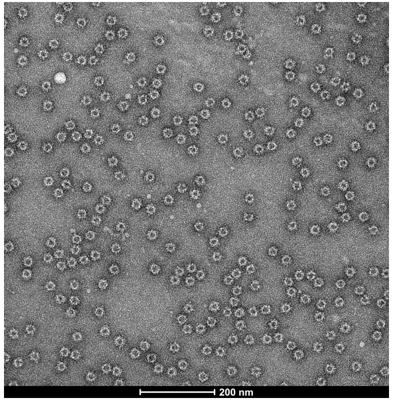

Day 28 -80 C

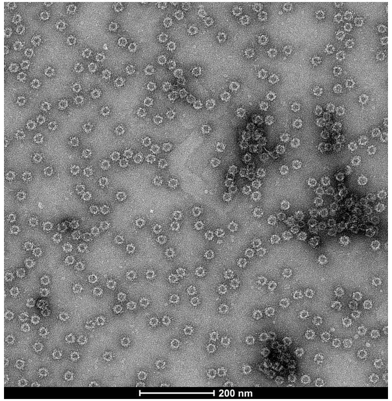

Day 28 2-8 C

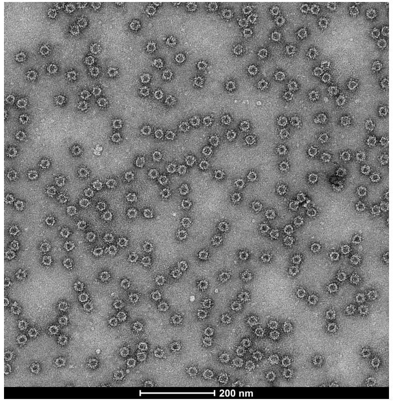

Day 28 22-25 C

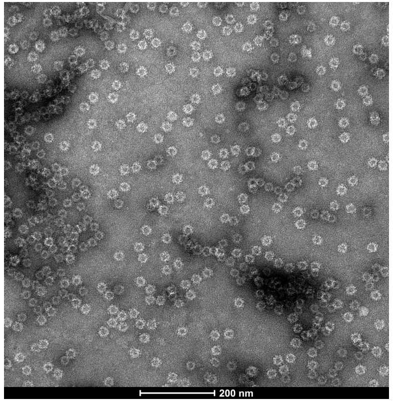

Day 28 35-40 C

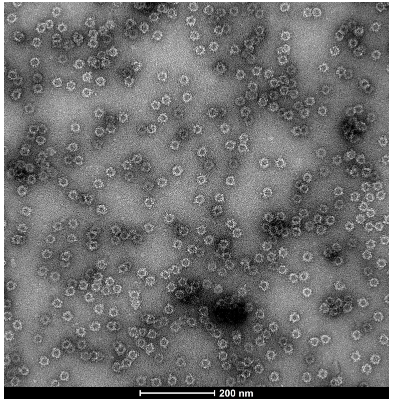

SDS-PAGE for cWu-1β9-RBD-NP

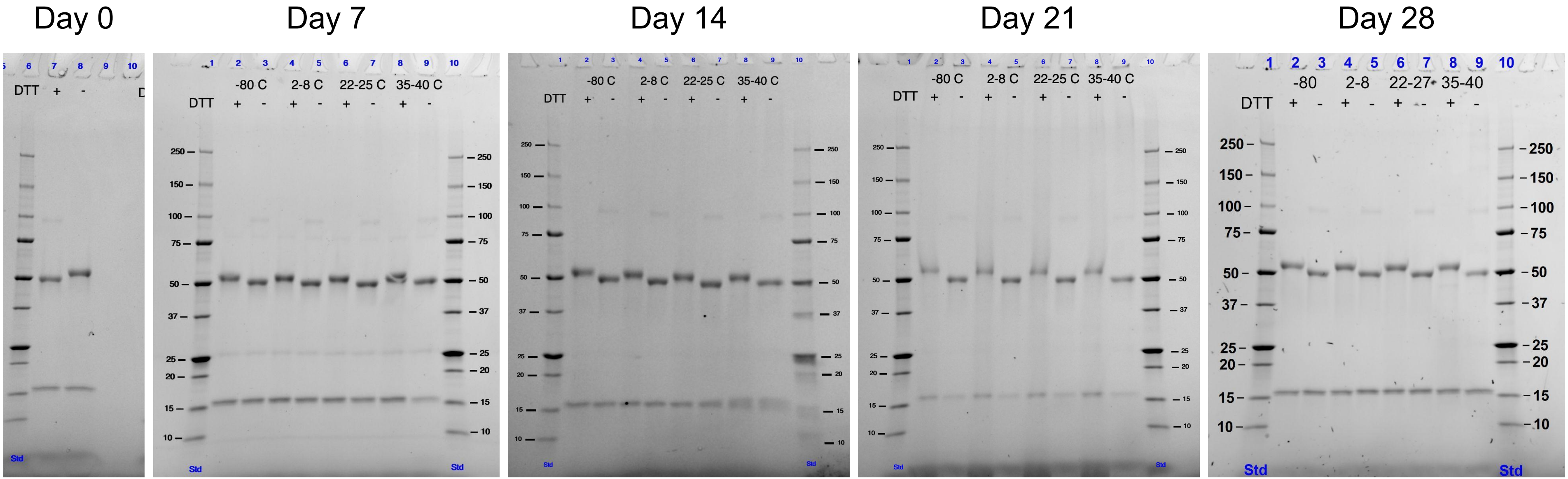

Dynamic Light Scattering for cWu-1β9-RBD-NP

Day 0

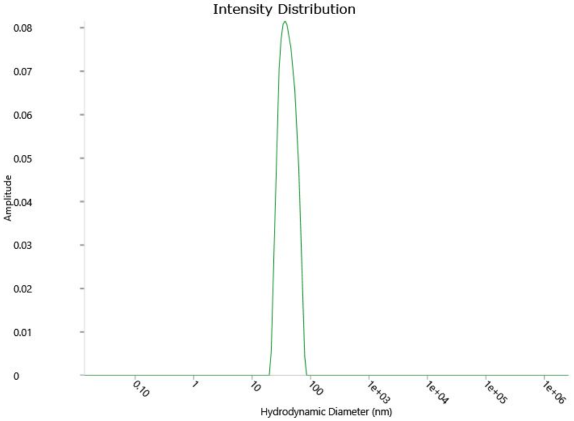

Day 7

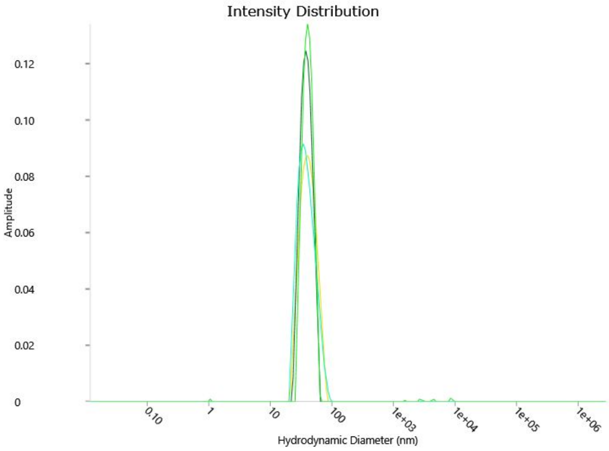

Day 14

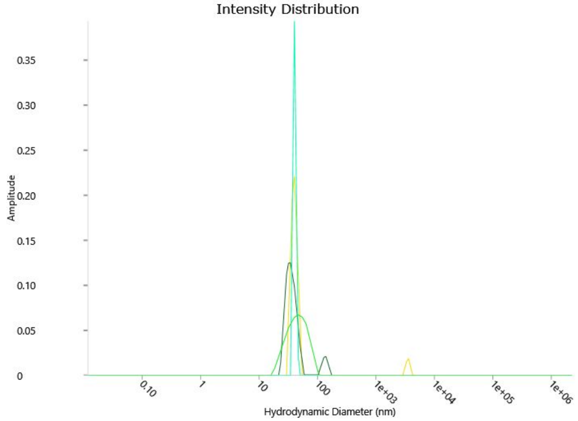

Day 21

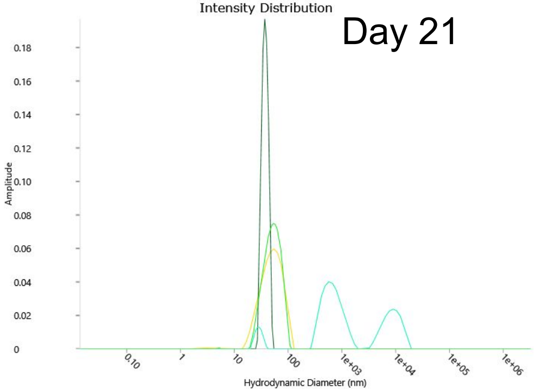

Day 28

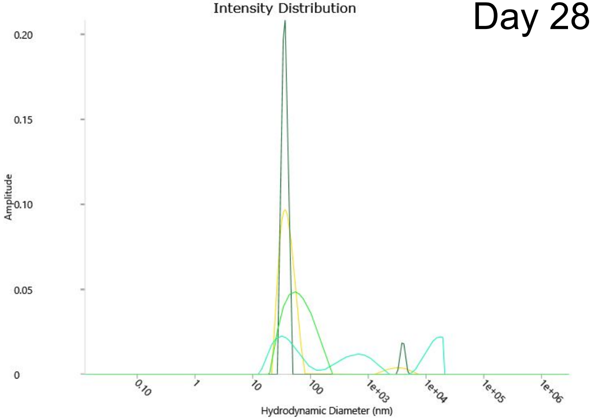

Absorbance at 320/280 for cWu-1β9-RBD-NP

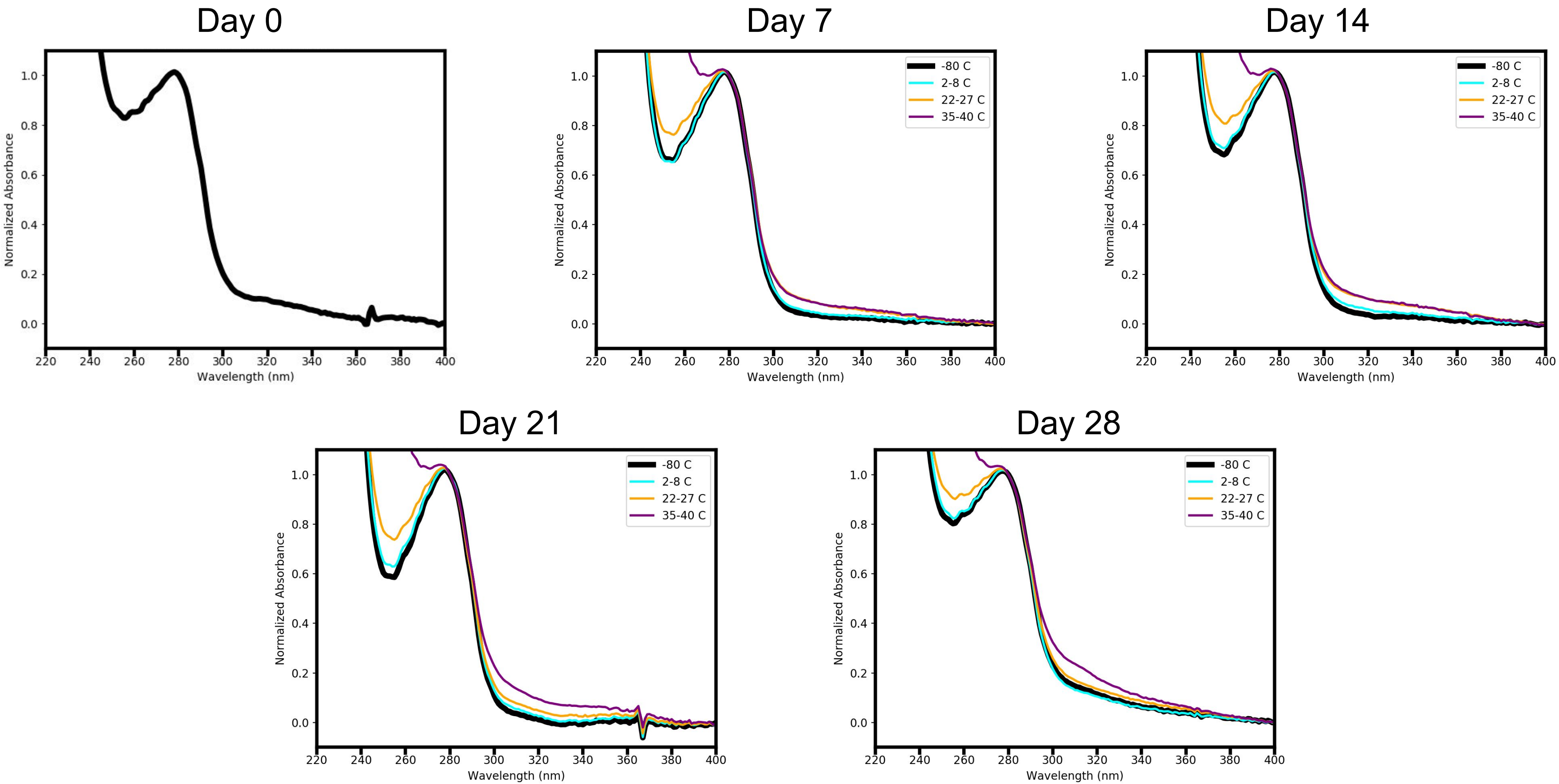

ACE2-Fc Binding Relative to <-70°C Reference for cWu-1β9-RBD-NP

Day 0

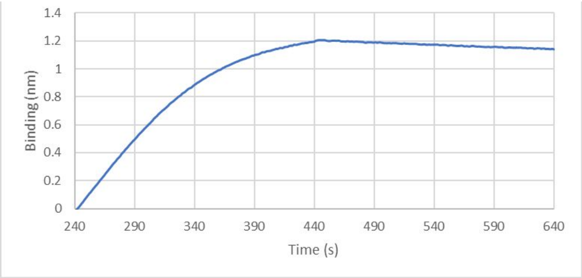

Day 7

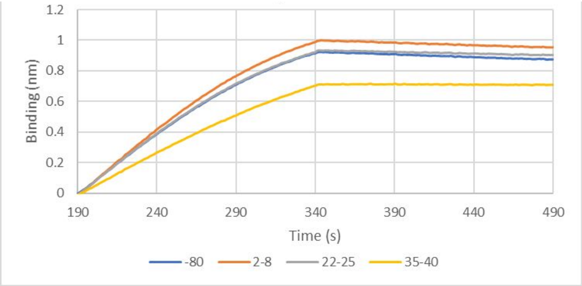

Day 14

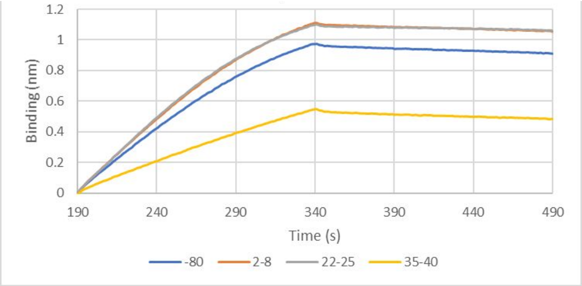

Day 21

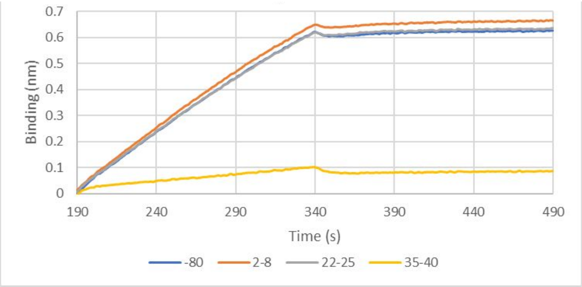

Day 28

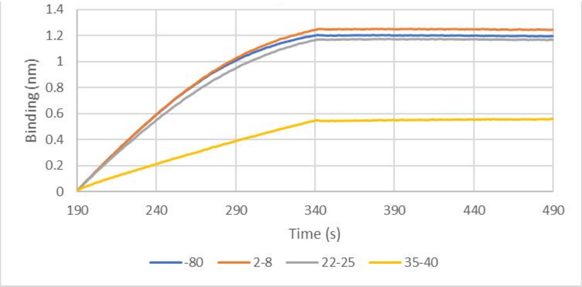

cWu-1γ-RBD-NP

nsEM for cWu-1γ-RBD-NP

Day 0

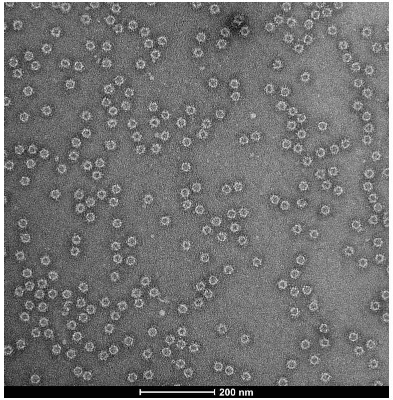

Day 28 -80 C

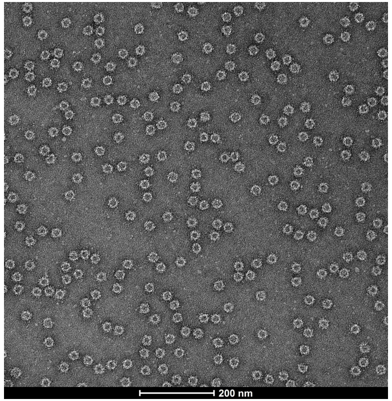

Day 28 2-8 C

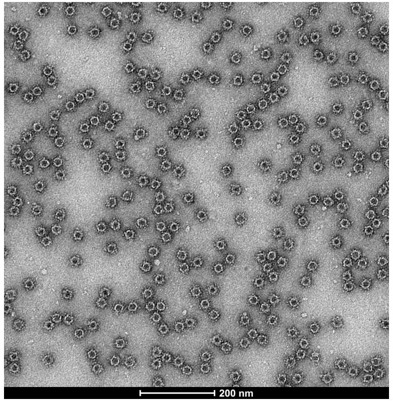

Day 28 22-25 C

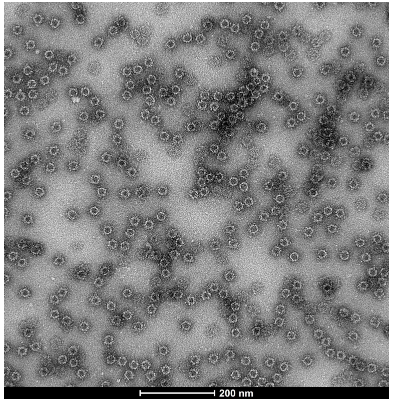

Day 28 35-40 C

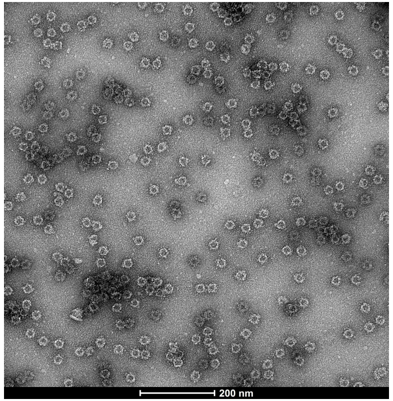

SDS-PAGE for cWu-1γ-RBD-NP

Day 0 Day 7 Day 14 Day 21 Day 28

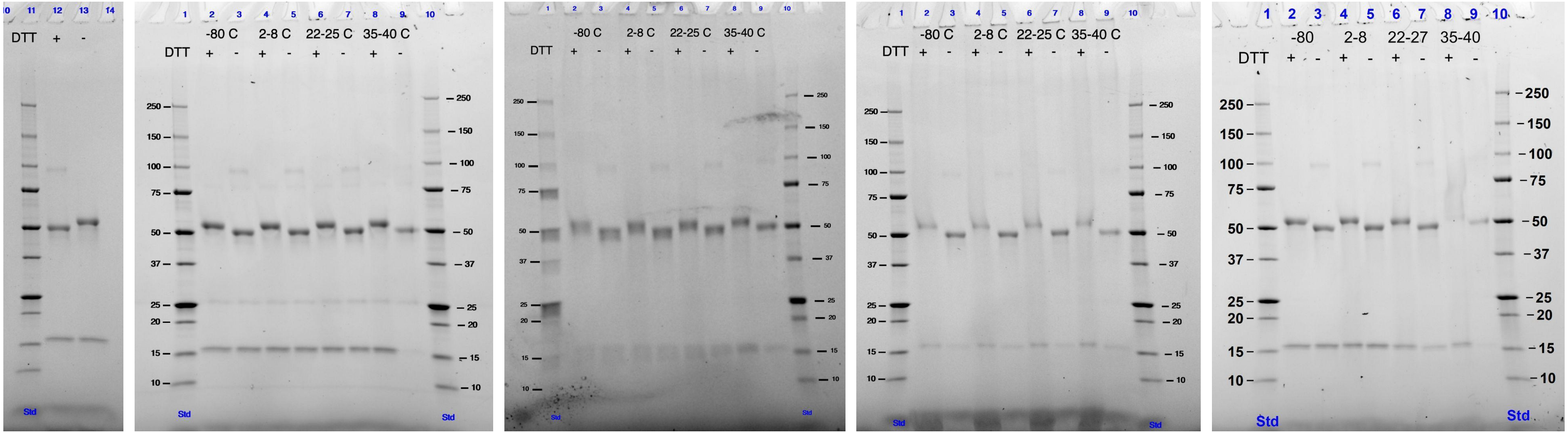

Dynamic Light Scattering for cWu-1γ-RBD-NP

Day 0

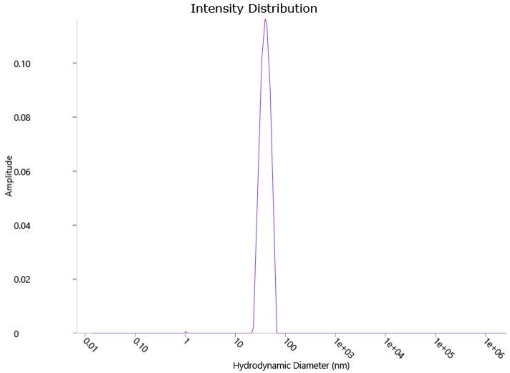

Day 7

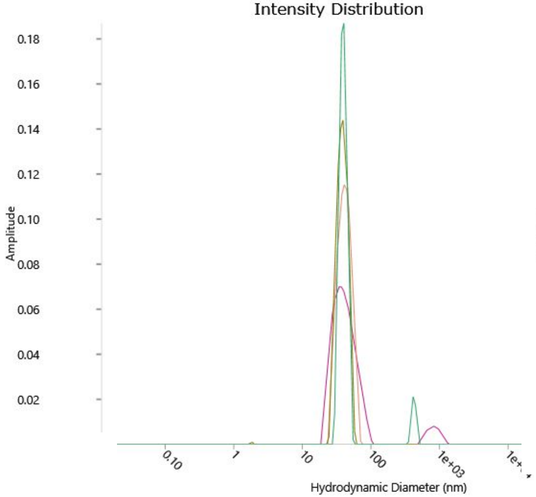

Day 14

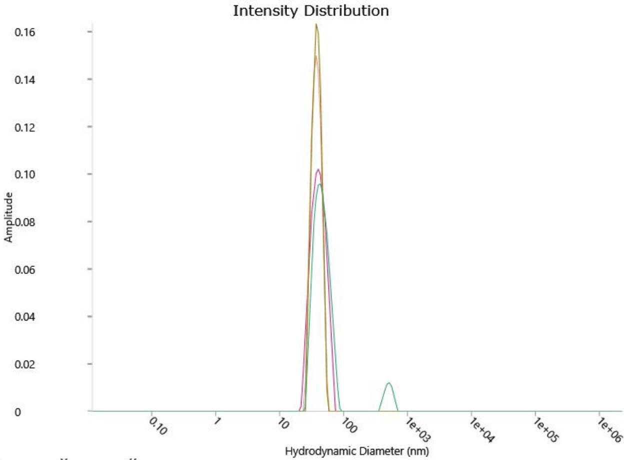

Day 21

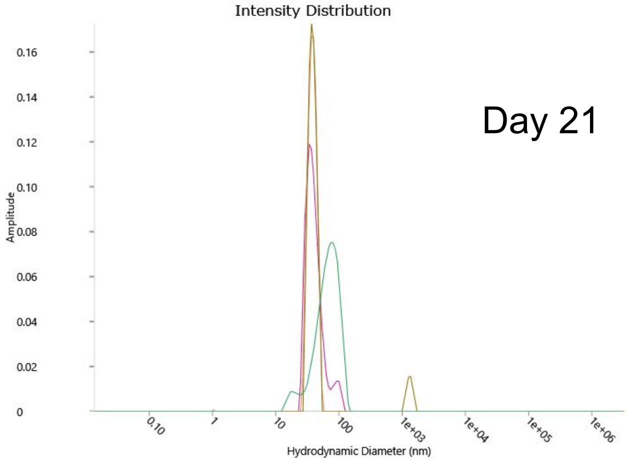

Day 28

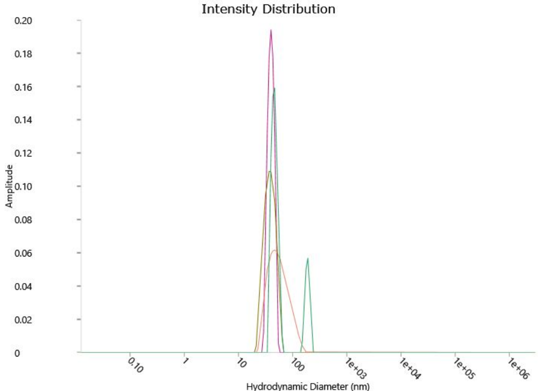

Absorbance at 320/280 for cWu-1γ-RBD-NP

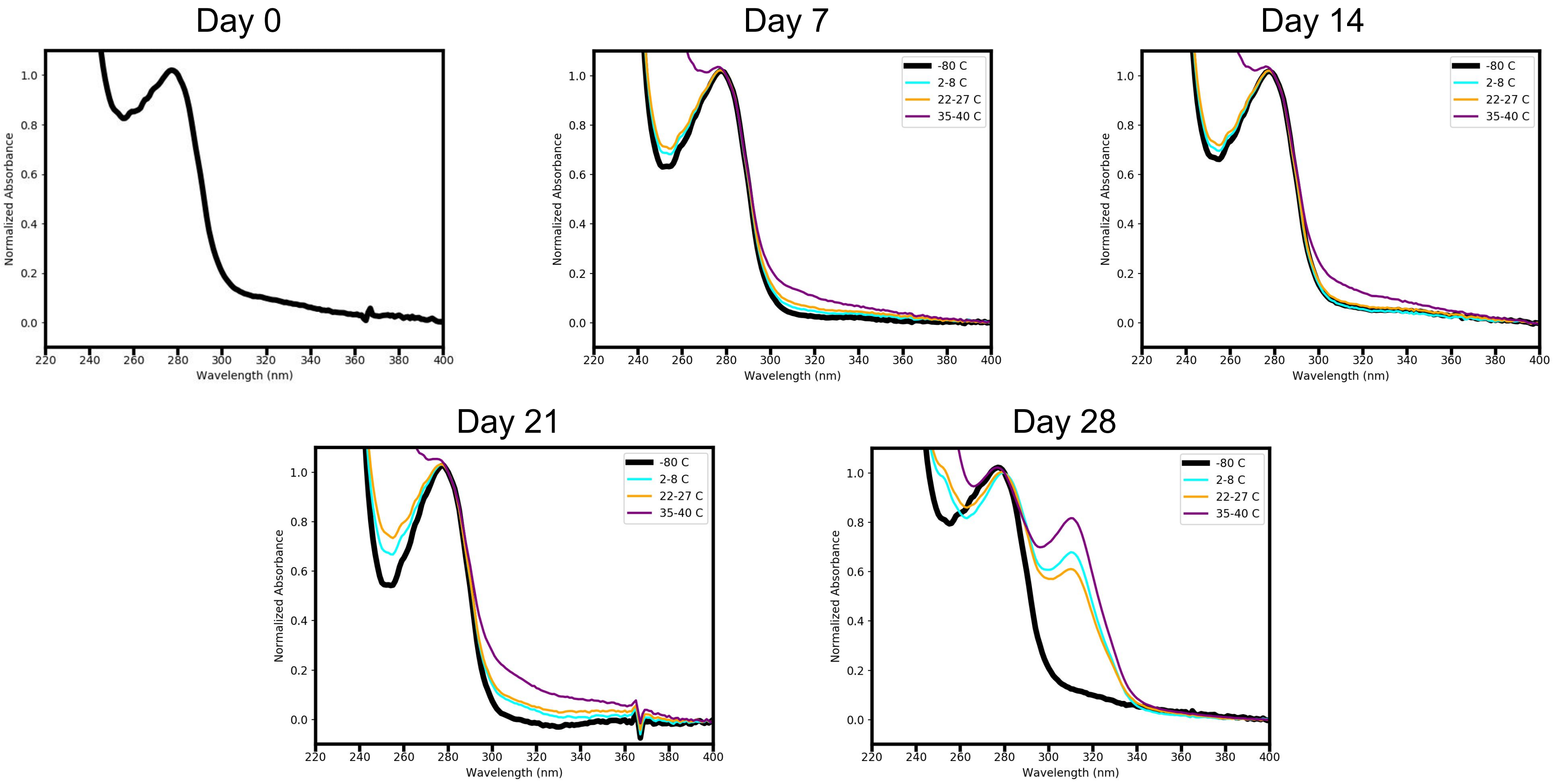

ACE2-Fc binding relative to <-70°C reference for cWu-1γ-RBD-NP

Day 0

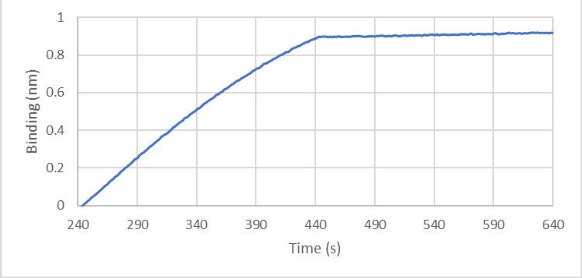

Day 7

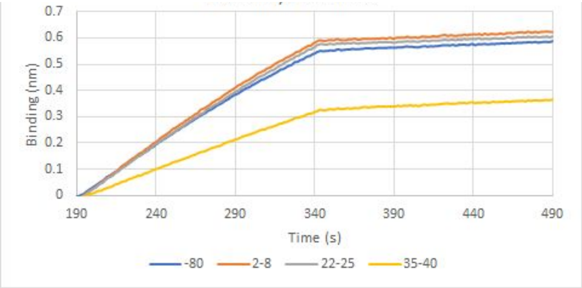

Day 14

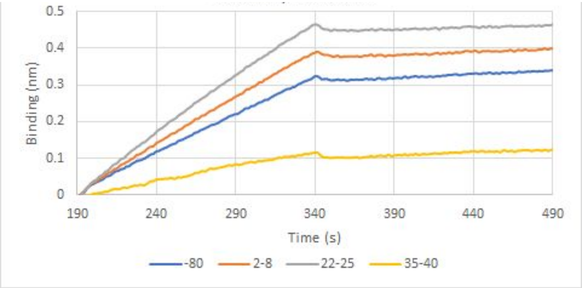

Day 21

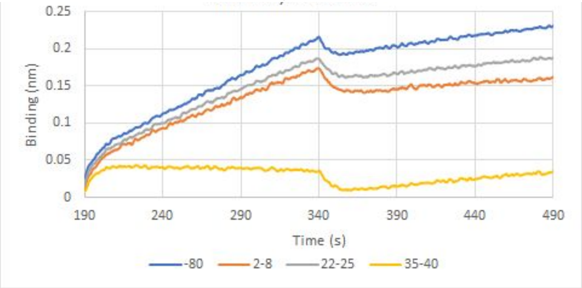

Day 28

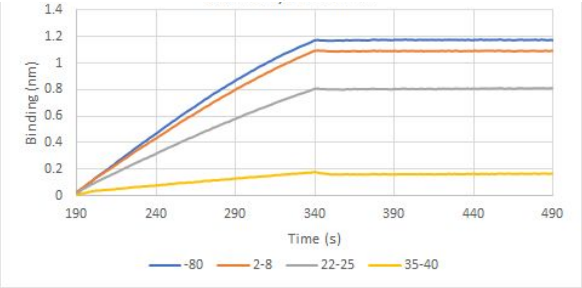

cWu-1γ9-RBD-NP

nsEM for cWu-1γ9-RBD-NP

Day 0

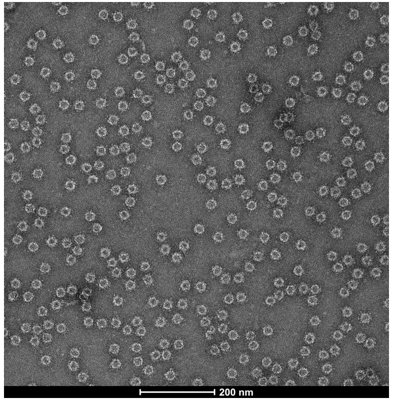

Day 28 -80 C

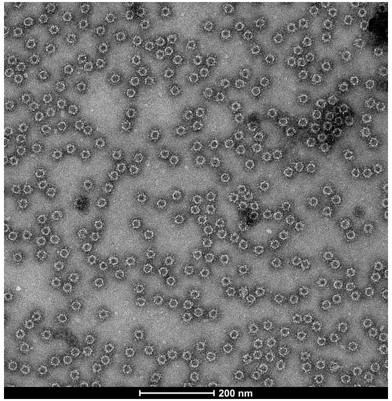

Day 28 2-8 C

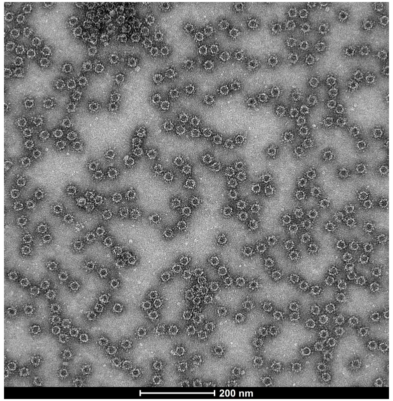

Day 28 22-25 C

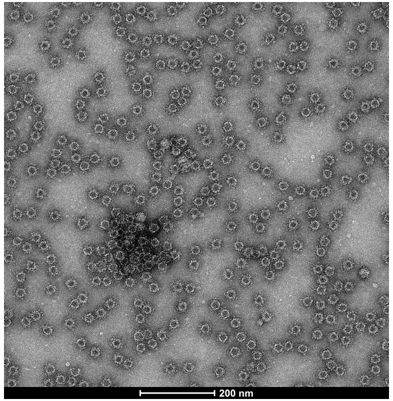

Day 28 35-40 C

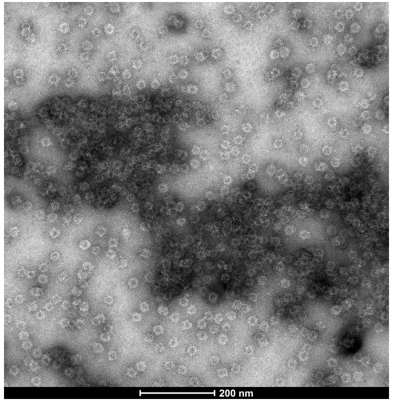

SDS-PAGE for cWu-1γ9-RBD-NP

Day 0 Day 7 Day 14 Day 21 Day 28

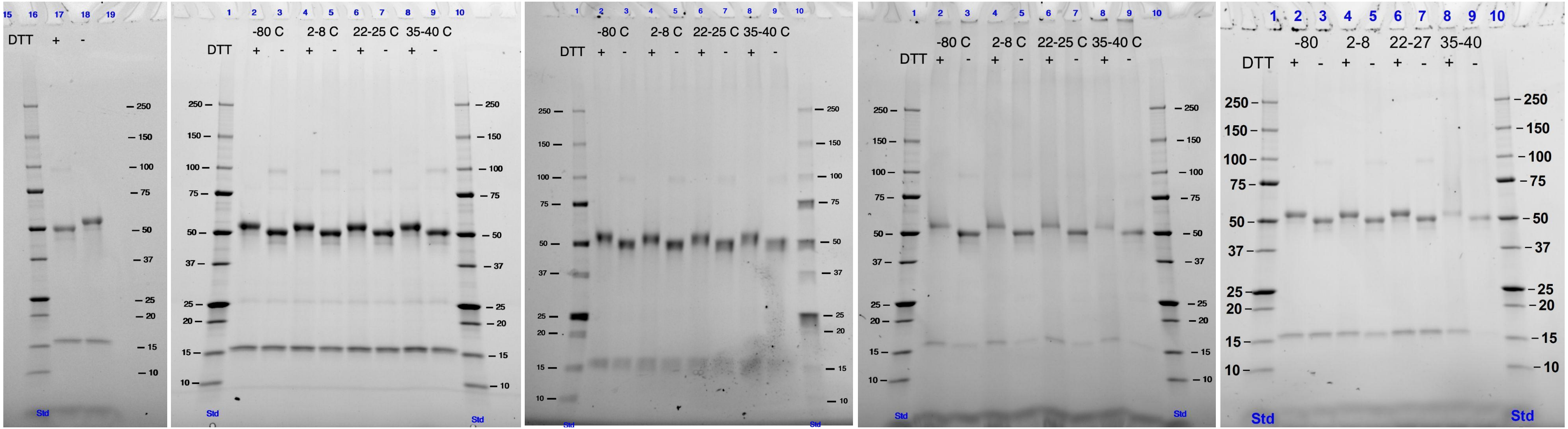

Dynamic Light Scattering for cWu-1γ9-RBD-NP

Day 0

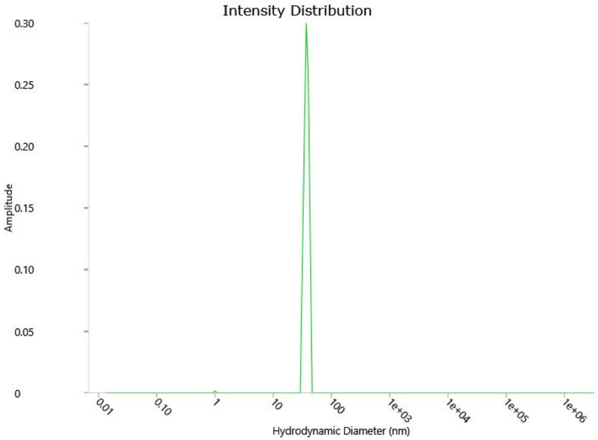

Day 7

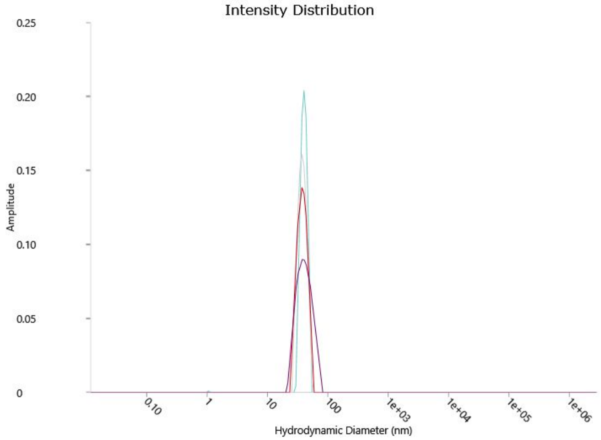

Day 14

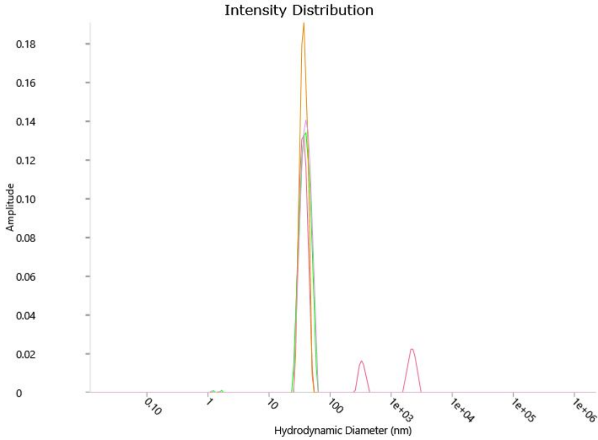

Day 21

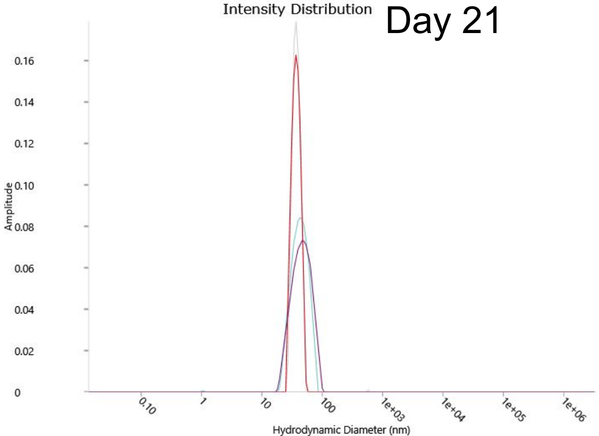

Day 28

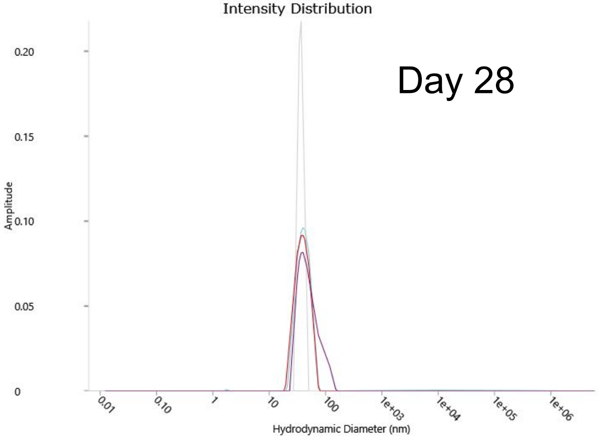

Absorbance at 320/280 for cWu-1γ9-RBD-NP

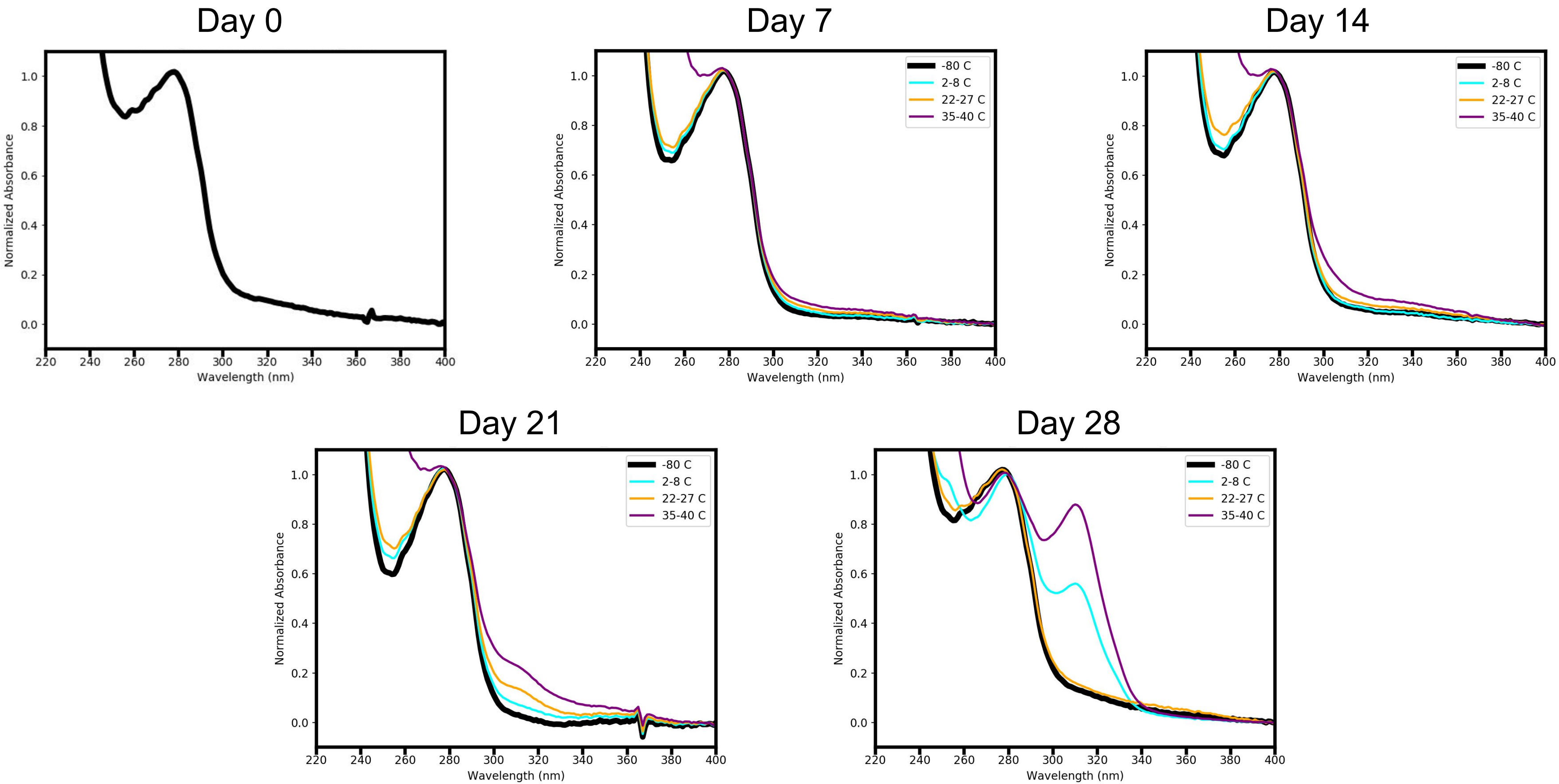

ACE2-Fc binding relative to <-70°C reference for cWu-1γ9-RBD-NP

Day 0

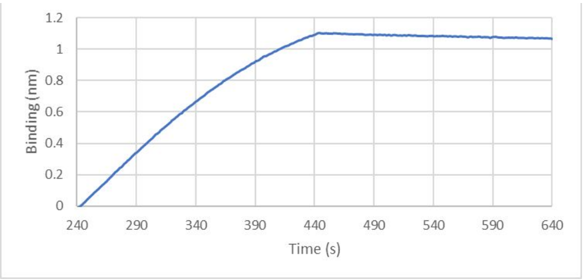

Day 7

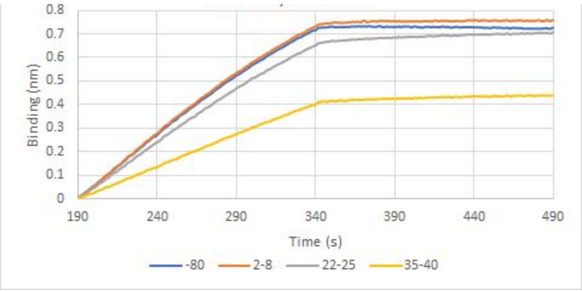

Day 14

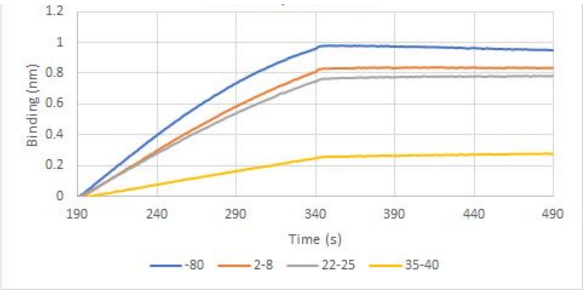

Day 21

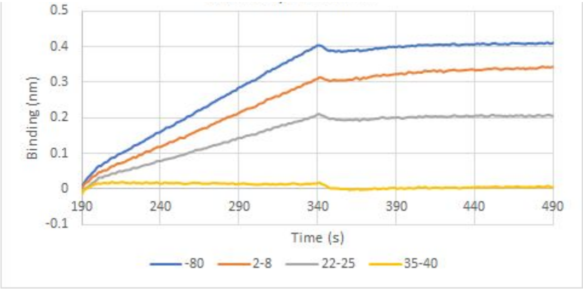

Day 28

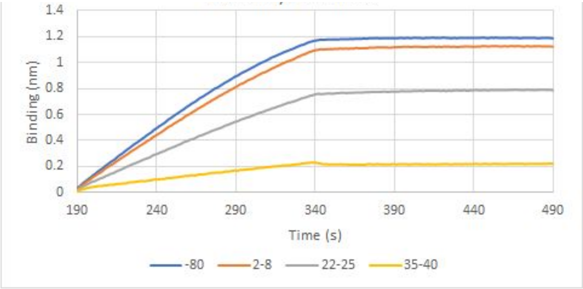

Biolayer Interferometry (BLI)

Week 1 BLI 20210907

Wu-1-RBD-NP

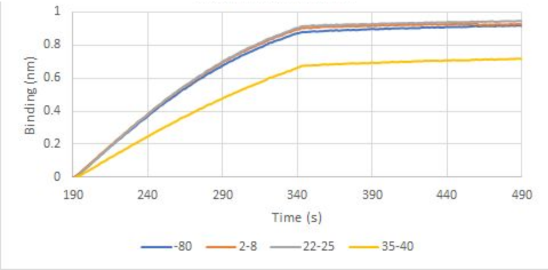

β-RBD-NP

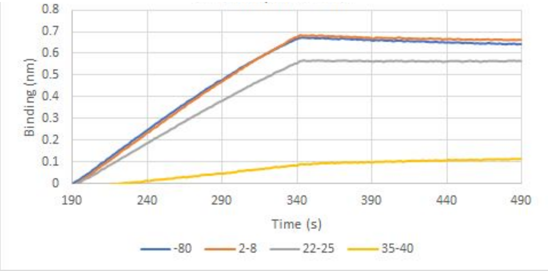

β9-RBD-NP

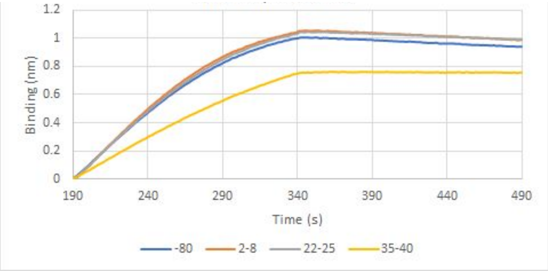

γ-RBD-NP

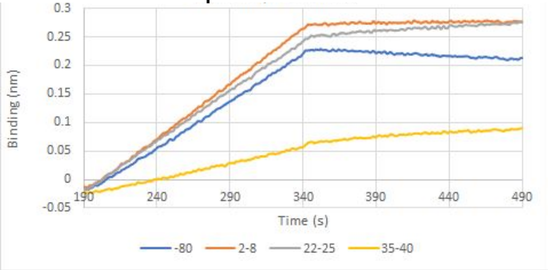

γ9-RBD-NP

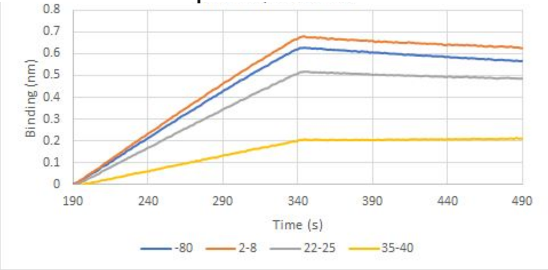

cWu-1β-RBD-NP

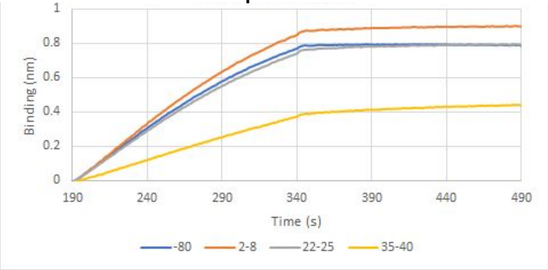

cWu-1β9-RBD-NP

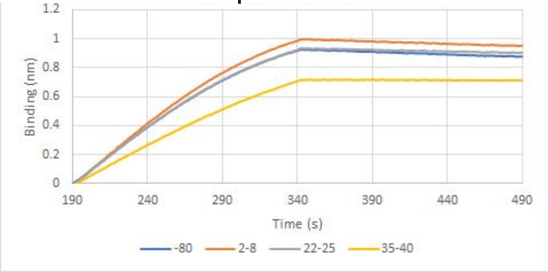

cWu-1γ-RBD-NP

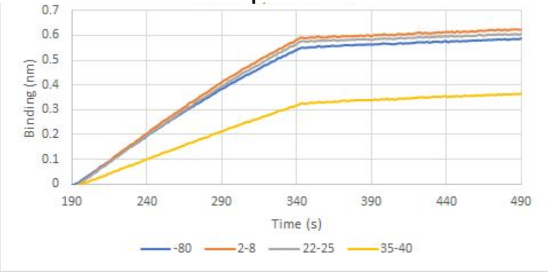

cWu-1γ9-RBD-NP

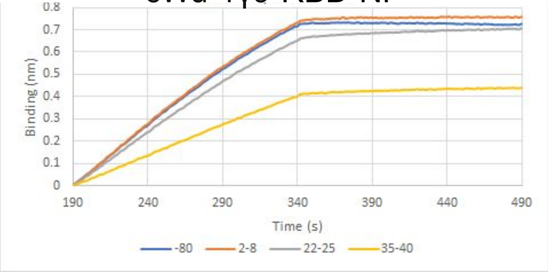

Week 2 BLI 20210915

Wu-1-RBD-NP

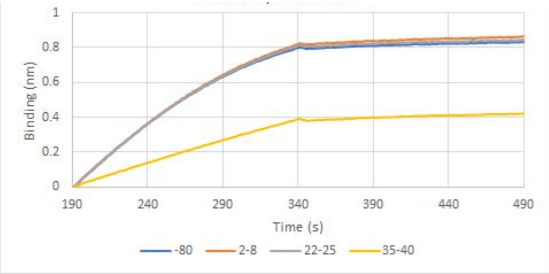

β-RBD-NP

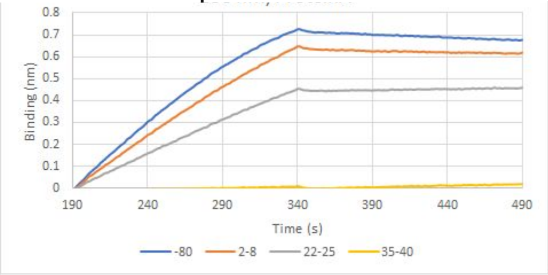

β9-RBD-NP

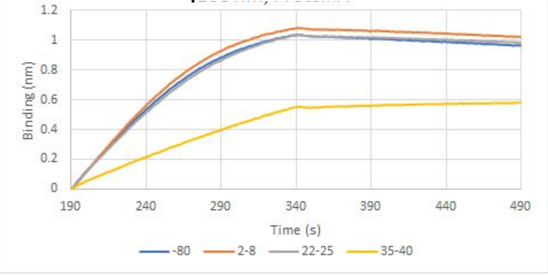

γ-RBD-NP

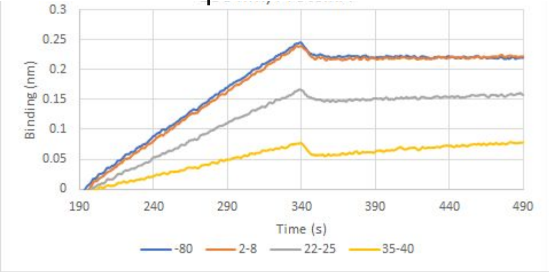

γ9-RBD-NP

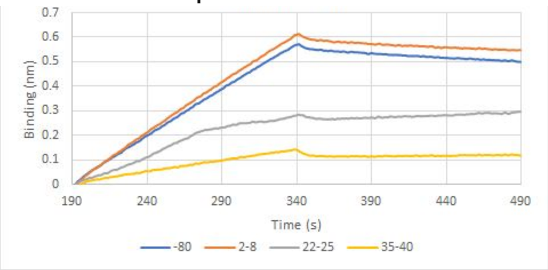

cWu-1β-RBD-NP

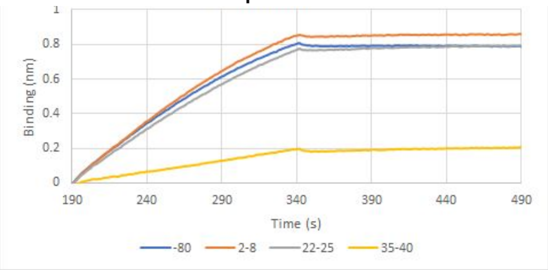

cWu-1β9-RBD-NP

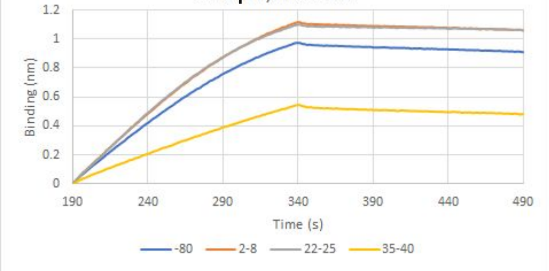

cWu-1γ-RBD-NP

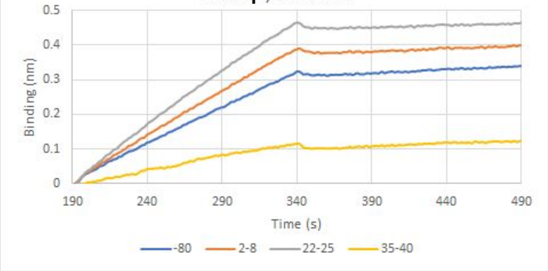

cWu-1γ9-RBD-NP

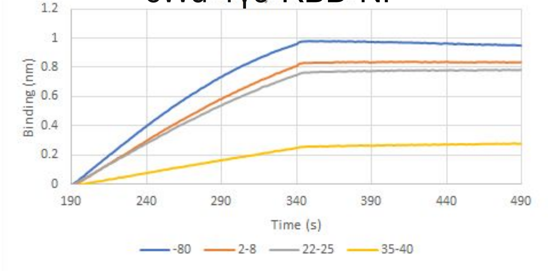

Week 3 BLI 20210921&23

Wu-1-RBD-NP

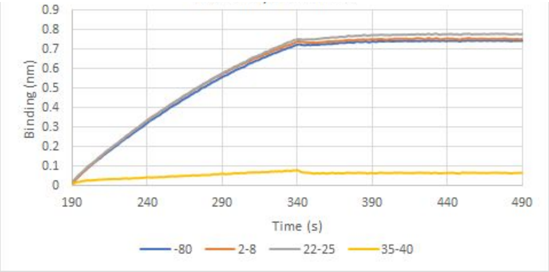

β-RBD-NP

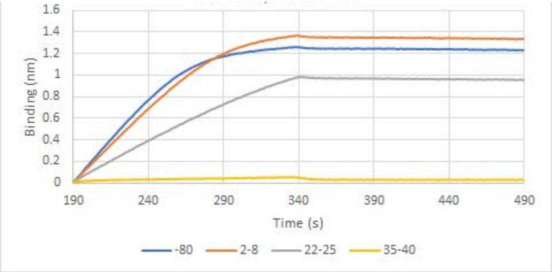

β9-RBD-NP

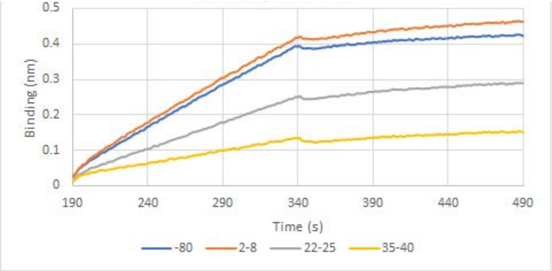

γ-RBD-NP

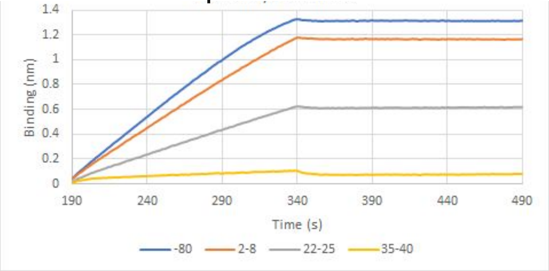

γ9-RBD-NP

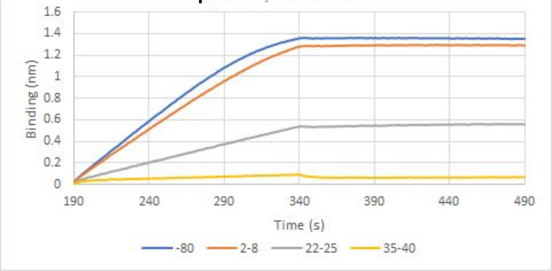

cWu-1β-RBD-NP

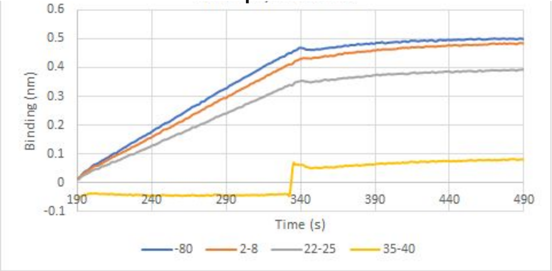

cWu-1β9-RBD-NP

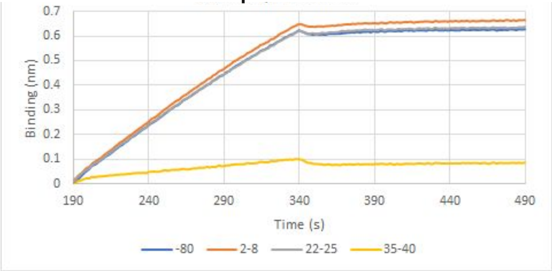

cWu-1γ-RBD-NP

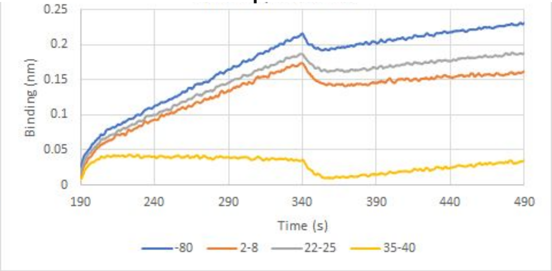

cWu-1γ9-RBD-NP

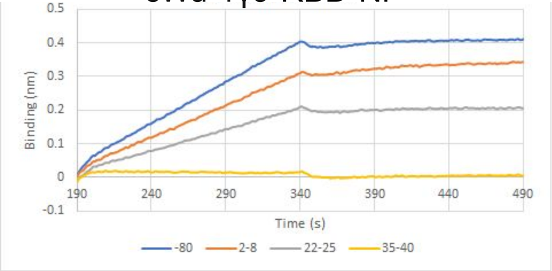

Week 4 BLI 20210928

Wu-1-RBD-NP

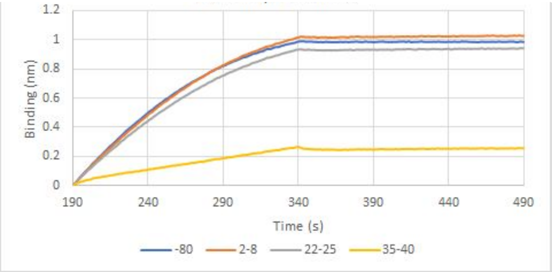

β-RBD-NP

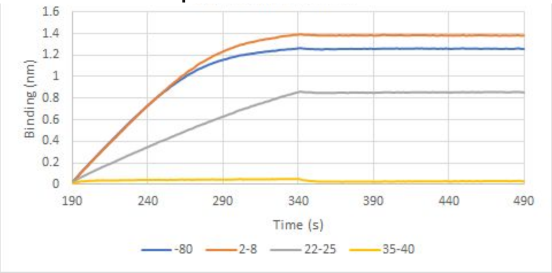

β9-RBD-NP

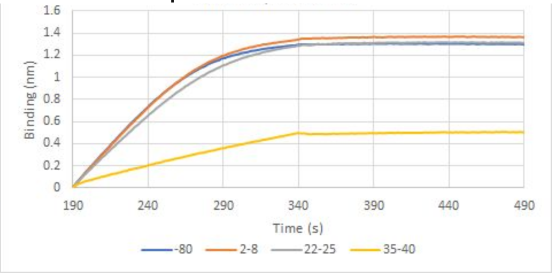

γ-RBD-NP

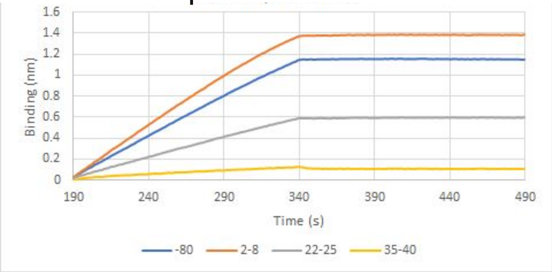

γ9-RBD-NP

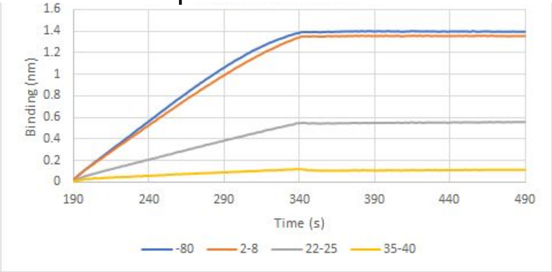

cWu-1β-RBD-NP

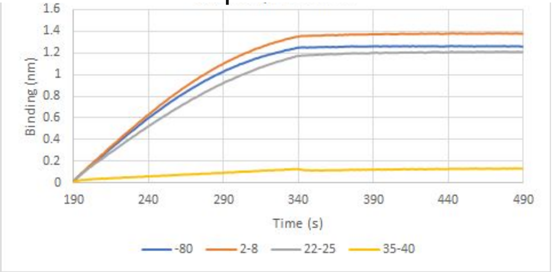

cWu-1β9-RBD-NP

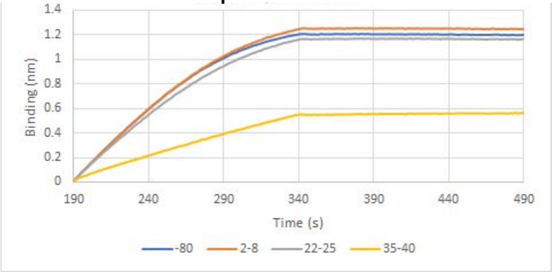

cWu-1γ-RBD-NP

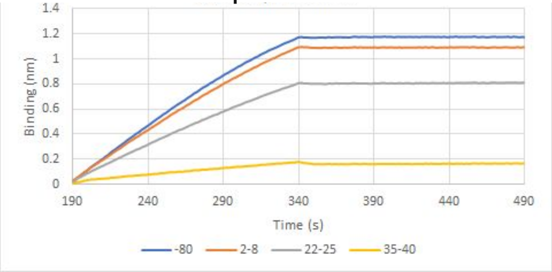

cWu-1γ9-RBD-NP

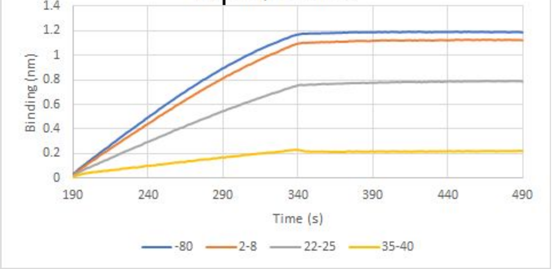

Supplement: Supplementary file 2 — Supplementary File 1 [file 41541_2024_982_MOESM2_ESM.pdf]
